# Reductive Dimerization of CO by a Na/Mg(I) Diamide

**DOI:** 10.1021/jacs.1c09467

**Published:** 2021-10-15

**Authors:** Han-Ying Liu, Ryan J. Schwamm, Samuel E. Neale, Michael S. Hill, Claire L. McMullin, Mary F. Mahon

**Affiliations:** Department of Chemistry, University of Bath, Claverton Down, Bath, BA2 7AY, U.K.

## Abstract

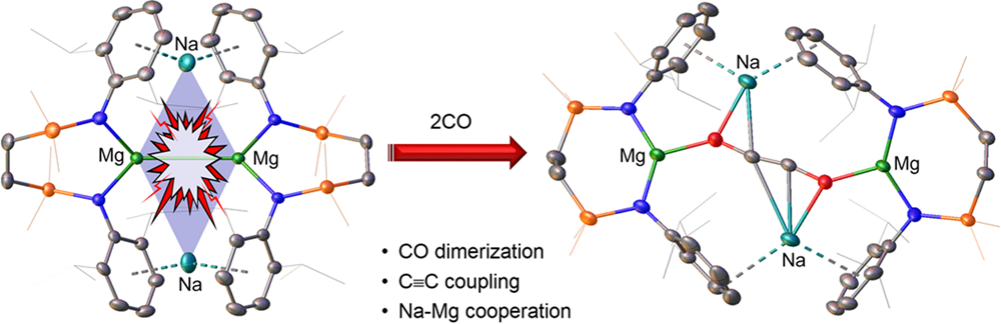

Sodium reduction of [{SiN^Dipp^}Mg] [{SiN^Dipp^} = {CH_2_SiMe_2_N(Dipp)}_2_; Dipp = 2,6-*i-*Pr_2_C_6_H_3_] provides the Mg(I) species, [{SiN^Dipp^}MgNa]_2_, in which the long Mg–Mg bond (>3.2 Å) is augmented by persistent Na–aryl interactions. Computational assessment indicates that this molecule is best considered to comprise a contiguous tetrametallic core, a viewpoint borne out by its reaction with CO, which results in ethynediolate formation mediated by the dissimilar metal centers.

## Introduction

**Chart 1:**
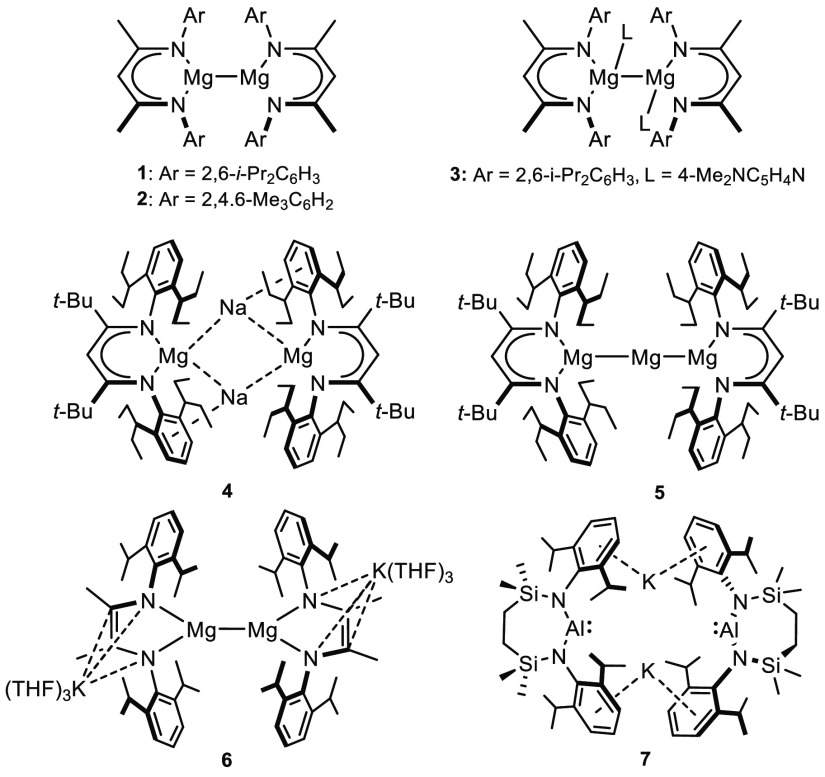
Structures of Compounds **1**–**7**

Prior to Jones and co-workers’ isolation of stable guanidinate and β-diketiminate (BDI) derivatives such as compound **1** in 2007 (Chart 1),^1−5^ experimental studies of low oxidation state Mg(I) derivatives were limited to transient species under low temperature matrix isolation conditions.^6^ Although more than 25 examples of such compounds have now been described, like **1**, a majority conform to the general structural requirement of a bulky monoanionic spectator ligand to provide the requisite kinetic stabilization of the [Mg–Mg]^2+^ unit against disproportionation to Mg(II) and Mg(0).

The implementation of an ever more bulky suite of amide and β-diketiminate anions, in harness with a variety of neutral mondentate bases, has, thus, resulted in the isolation of a variety of species comprising four-,^7,8^ three-,^9−15^ two-,^16^ or even asymmetric combinations of four- and three-,^17^ or three- and two-coordinate magnesium centers.^18^ The intermetallic distance in these compounds has proved remarkably malleable such that the Mg–Mg bond lengths encompass an unusually wide range, with extremes provided by an *N*-mesityl BDI variant of **1** [**2**, Mg–Mg 2.808(1) Å]^8^ and compound **3** [3.1962(14) Å] in which the coordination number of both magnesium atoms in **1** is raised to four through the introduction of two molecules of strongly basic 4-dimethylaminopyridine (DMAP).^7^ While this deformability is consistent with a very shallow bond potential energy surface, experimental charge density studies and quantum theory of atoms-in-molecules (QTAIM) calculations revealed that the Mg–Mg bond of compound **1** is not a classical covalent interaction. Rather, a large region of negative Laplacian in the Mg–Mg internuclear region comprises a non-nuclear attractor (NNA) containing 0.8 electrons, in effect electron density that is not associated with a particular nucleus and, hence, distinct from the positions of the magnesium atoms.^9,18−20^

While a pre-existing [Mg–Mg]^2+^ unit has also been manipulated to provide further Mg(I) derivatives,^21,22^ the synthesis of these compounds invariably involves the generation of a salt by reduction of a magnesium(II) halide starting material with either sodium or potassium. The current apotheosis of this approach has very recently been provided by Harder and co-workers’ report that sodium reduction of a magnesium(II) iodide bearing the extraordinarily bulky BDI* [BDI* = HC{C(*t*-Bu)N(DiPeP)}_2_; DiPeP = 2,6-(3-pentyl)-phenyl] ligand provides the remarkable magnesium(0) compound, [(BDI*)MgNa]_2_ (**4**).^23^ Whereas the Mg···Mg separation [5.7792(5) Å] in **4** is too long to represent a bonding interaction, the shorter of the Mg–Na distances [3.1216(7) Å] approaches the sum of the ionic radii of the respective group 1 and group 2 metal centers (3.19 Å).^24^ Compound **4** also disproportionates in benzene to provide a further Mg(0)-containing species, [(BDI*)MgMgMg(BDI*)] (**5**), in which the magnesium atoms were shown by QTAIM to be connected via two NNAs, each with a basin of 0.64 electrons.

An exception to this synthetic approach has been provided by Yang and co-workers’ utilization of doubly reduced α-diimine, phenanthrene-9,10-diimine, and *o*-phenylene diamide ligand sets.^10,14^ In these cases the redox noninnocent pro-ligands were reduced with excess potassium metal, albeit the reactions were performed in the presence of MgCl_2_ and so are again driven by the thermodynamically favorable production of an ionic group 1 salt. Charge balance in the resultant Mg(I) species, such as **6**,^10^ is necessarily maintained by the incorporation of potassium cations. Although these cations interact strongly with the delocalized π system of the planar dianionic spectator ligands, they impose a negligible impact on the bonds between the three-coordinate Mg centers, which are <3 Å in all cases.

In contrast to the unsupported Mg–Mg bonds of compounds **1**–**3** and **6**, a notable feature of compound **4** is the presence of bridging Na–aryl contacts, which presumably play a significant role in the stability of the dimeric molecule.^23^ We, and others, have recently observed that similarly persistent Na– and K–aryl interactions provide a defining feature in a number of dimeric potassium diamidoalumanyl derivatives.^25−28^ In these cases, the integrity of the formal Al(I) centers is maintained by a sterically demanding diamide ligand, such as in the seven-membered cyclic species, [{SiN^Dipp^}AlK]_2_[{SiN^Dipp^} = {CH_2_SiMe_2_N(Dipp)}_2_; Dipp = 2,6-*i-*Pr_2_C_6_H_3_] (**7**).^28^ Prompted by the robust nature of compounds **4** and **7**, in this contribution we show that the thermodynamic preference for heavier group 1 element–aryl interactions^29,30^ can provide a strategy to access dinuclear low oxidation state alkaline earth species that circumvents conventional salt elimination.

## Results and Discussion

**Scheme 1:**
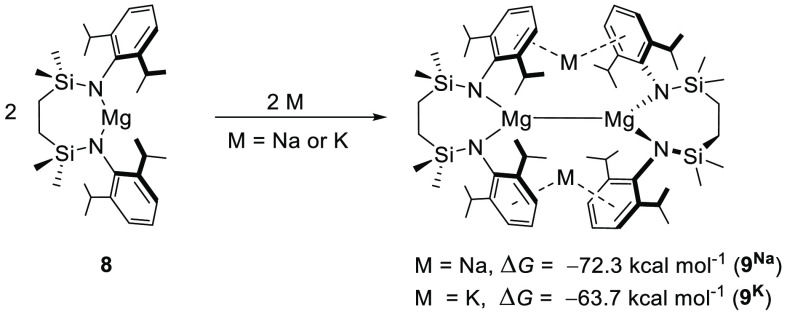
DFT-Computed (BP86-D3BJ/BS2(benzene)//BP86/BS1) Free Energy Changes for the Sodium and Potassium Reduction of Compound **8**

Initial DFT calculations were performed to model the sodium and potassium reduction of a neutral magnesium derivative, [{SiN^Dipp^}Mg](**8**), supported by the amide ligand utilized in compound **7**. While these results supported the thermodynamic viability of reduction with both alkali metals to the putative dinuclear Mg–Mg bonded species (**9**^**Na/K**^, Scheme 1), the sodium-based process was calculated to be more exergonic (M = Na, Δ*G* = −72.3; M = K, Δ*G* = −63.7 kcal mol^–1^).

Encouraged by these results, compound **8** was synthesized by reaction of the aniline pro-ligand, {SiN^Dipp^}H_2_, with dibutyl magnesium in hexane at room temperature. Although **8** was isolated in analytically pure base-free form by removal of volatiles, it was observed to quickly sequester aromatic solvents. Compound **8** was, thus, characterized as its Mg-η^1^-benzene and Mg-η^1^-toluene adducts by X-ray diffraction analysis performed on colorless single crystals isolated by slow evaporation of the respective arene solutions (Figure S14). Guided by the greater theoretical viability of the sodium reduction, a benzene solution of **8** was reacted with an excess of 5 wt % Na/NaCl at room temperature for 12 h,^31^ whereupon removal of volatiles and crystallization from toluene solution provided compound **9** as highly air-sensitive bright yellow crystals. The ^1^H NMR spectrum of compound **9** was characterized by a significant asymmetry across the various {SiN^Dipp^} ligand environments. This was particularly apparent in the silylmethyl signals, which appeared as two (6H) singlets at δ 0.52 and −0.21 ppm, and which may be attributed to a loss of the mirror plane of symmetry through the seven-membered magnesium chelate of **8**. Notably, these resonances did not display any level of coherence transfer consistent with chemical or conformational exchange in the corresponding EXSY NMR experiment.

**Figure 1:**
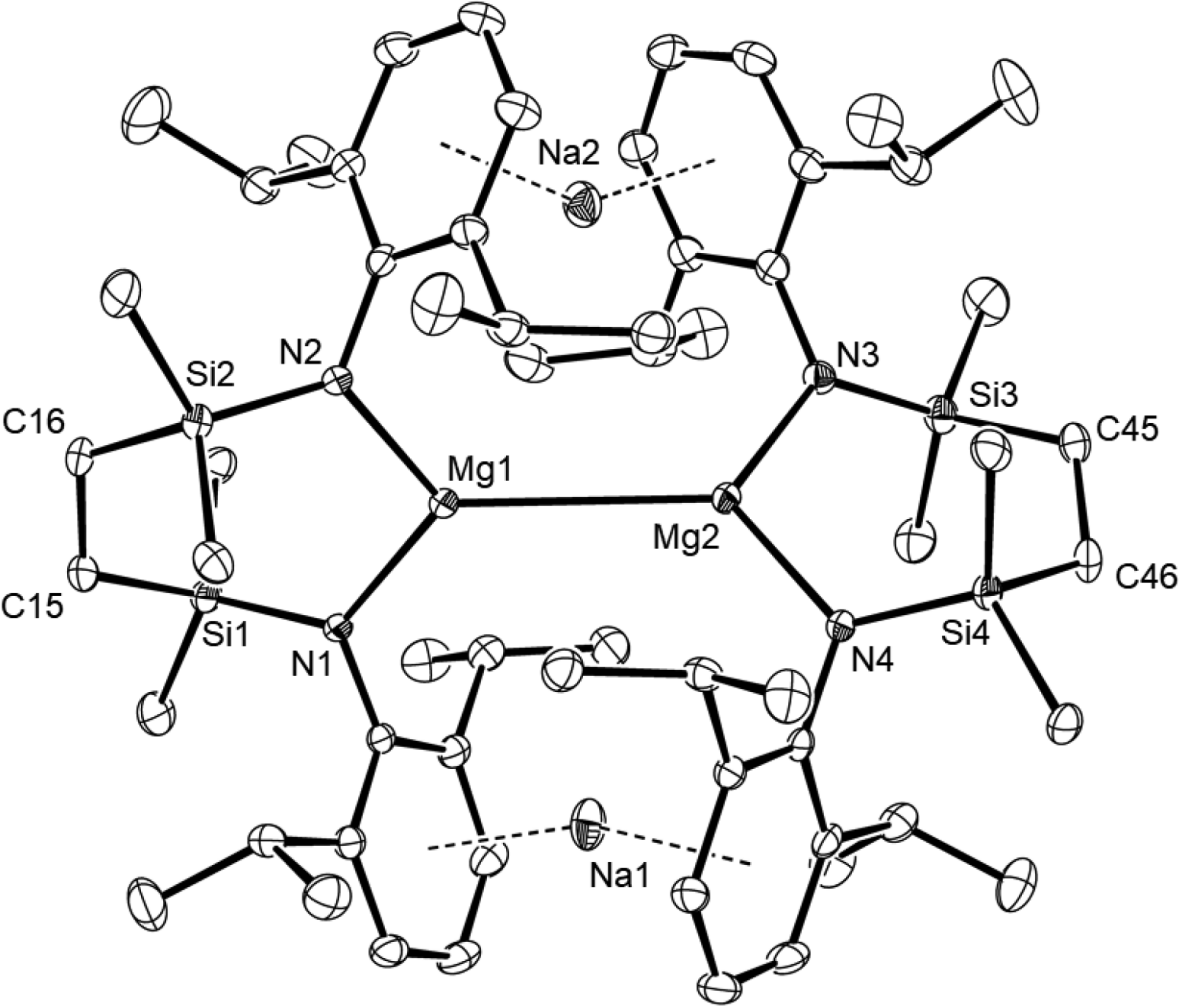
ORTEP (30% probability) of the Mg1/Mg2-containing molecule in compound **9**. Hydrogen atoms and disordered molecules of solvent are omitted for clarity. Selected bond lengths (Å) and angles (deg): Mg1–Mg2 3.2077(10), Mg3–Mg4 3.2124(11), Mg1–Na2 3.7229(13), Mg1–N1 2.0843(18), Mg1–N2 2.0786(19), Mg2–Na2 3.7014(13), Mg2–N3 2.090(2), Mg2–N4 2.0794(19), Mg3–Na3 3.6290(12), Mg4–Na3 3.6691(12), N2–Mg1–N1 109.76(7), N3–Mg2–N4 110.77(8), N5–Mg3–N6 110.03(8), N8–Mg4–N7 110.41(8).

The origin of these observations became apparent in a subsequent single crystal X-ray diffraction analysis of compound **9**, which confirmed it as a tetranuclear heterobimetallic species in which a pair of {SiN^Dipp^}Mg units are bridged by twofold η^6^-Na-Dipp interactions (Figure 1). Intrinsic to the crystallographic symmetry (*P*1), the asymmetric unit comprises a racemate, such that the Mg1/Mg2- and Mg3/Mg4-containing molecules describe right- and left-handed helices, respectively. While there are some significant variations across the comparable metric data of both molecules, their gross constitutions are effectively identical. The most notable features of the structures are the exceptionally long magnesium–magnesium separations [Mg1–Mg2 3.2077(10), Mg3–Mg4 3.2124(11) Å], which exceed even that reported for compound **3** [3.1962(14) Å]. Consistent with the attribution of a Mg(I) oxidation level to magnesium, the N–Mg bonds observed in **9** [avg. 2.0831 Å] are more closely commensurate with the N–Mg distances in compound **1** [avg. 2.0604 Å] than the longer formal nitrogen-to-Mg(0) interactions [avg. 2.117 Å] displayed by compound **4**. The closest Na–Mg separation in **9** [Mg3–Na3 3.6290(12) Å] is also elongated in comparison to even the longer of the Na–Mg contacts reported for compound **4** [3.4529(7) Å], in which a modicum of Mg–Na bonding was supported by bond paths located in the QTAIM analysis. In contrast, the Na–C_centroid_ distances to the Dipp substituents [avg. 2.414 Å] of **9** are significantly contracted in comparison to the Na–C_centroid_ separations observed in compound **4** [2.604 Å]. In this latter case, the Na-coordinated and uncoordinated *N*-aryl substituents observed in the solid-state structure could not be discriminated by NMR spectroscopy, indicating rapid solution exchange of both sites.^23^ This observation contrasts significantly with the behavior of **9**, wherein a mutually gauche orientation of each dimer half in the molecule is evidently “locked” into the solid-state conformation by a combination of persistent Mg–Mg and Na-Dipp interactions. As a result, the enantiomers observed in the solid-state structure cannot interconvert, imposing the loss of symmetry across the various {SiN^Dipp^} ligand environments implied by NMR spectroscopy.

**Figure 2:**
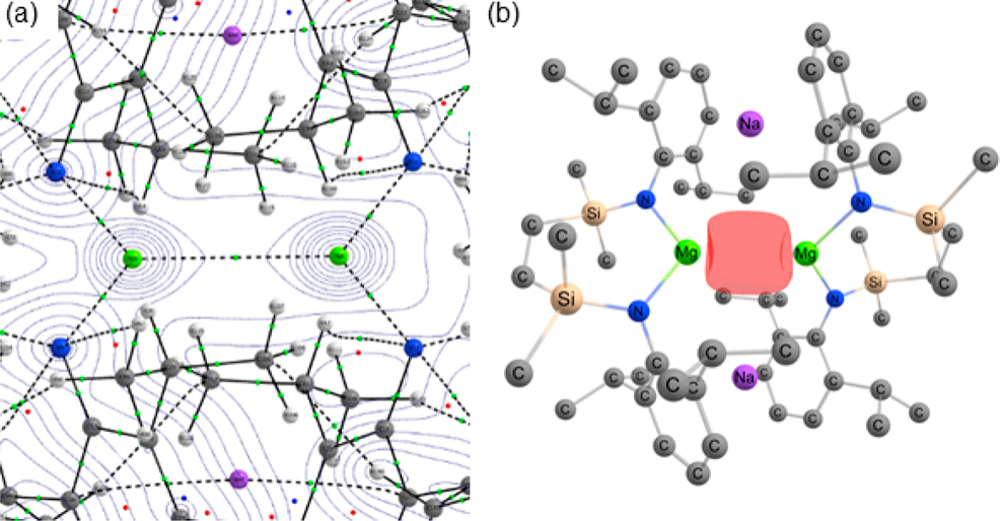
(a) QTAIM molecular graph of the BP86-optimized geometry of **9**. The electron density contours are computed in the {Mg–N} planes with bond critical points (BCPs) shown as small green spheres. (b) Natural Localized Molecular Orbital of the Mg–Mg bond in **9**.

Insight into the electron densities and, in turn, the nature of the interatomic interactions within compound **9**, was obtained with QTAIM topological analysis. The Mg–Mg bond critical point (ρ = 0.0194) has a negative energy density (*H*(*r*) = −0.00362) and Laplacian (∇^2^ρ(r) = −0.0136) which are indicative of a stabilizing covalent bond (Figure 2a). This is further supported by NBO analysis through the identification of a natural localized molecular orbital of a Mg–Mg σ-bond with a roughly equal contribution from the 3s orbitals of each Mg center (Figure 2b). In contrast to comparable calculations performed on monometallic species such as **1** and **5**,^19,20^ no persuasive evidence of a NNA could be identified. Rather, QTAIM analysis revealed two further weak bond critical points (where ρ = 0.0034) between the Na cations and the Mg–Mg bond critical point itself. Perturbation energy analysis of this unusual interaction with both Na^+^ cations estimates an overall σ-donation strength between the Mg–Mg bond and each Na^+^ cation of Δ*E*^(2)^ ≈ 25 kcal mol^–1^.

Magnesium(I) complexes comprising an isolated Mg–Mg interaction have displayed broad applicability as soluble molecular reducing agents.^4^ The QTAIM and NBO results suggest, however, that the chemistry of **9** is better considered in terms of its [Na_2_Mg_2_]^4+^ core rather than as an isolated [Mg–Mg]^2+^ unit. This raises the possibility that new patterns of cooperative reactivity may arise between the dissimilar metal centers. Treatment of molten alkali metals or their solutions in liquid ammonia with gaseous CO have long been known to give rise to ill-defined and shock-sensitive mixtures of oxocarbon anions, [C_*n*_O_*n*_]^2–^ (*n* = 2–6).^32,33^ Although molecules such as **1**–**3** are unreactive toward CO, Jones, Maron and co-workers have shown that addition of monodentate bases yields unsymmetrical Mg–Mg bonded species, [{(BDI)(D)Mg–Mg(BDI)}_2_] (D = DMAP or *N*-heterocyclic carbene), which do effect CO oligomerization.^17,34^ Although these reactions have provided derivatives of the deltate dianion, [C_3_O_3_]^2–^, DFT calculations indicated a trimerization mechanism that proceeds through the initial generation of reactive intermediates containing *trans-*bent ethynediolate, [O–C≡C–O]^2–^, dianions. In support of this hypothesis, DMAP adducts of the less encumbered *N-*mesityl (**2**) and *N*-*o*-xylyl variants were found to react with only two molecules of CO. Although the structures of the resultant bimetallic compounds comprised an unusual bridging *cis-*ethenediolate dianion, [μ-O(H)C=C(C_5_H_3_N-4-NMe_2_)O]^2–^, its formation was reasoned to be a result of intramolecular C–H activation of Mg-ligated DMAP by an initially formed [O–C≡C–O]^2–^ intermediate.^34^ In a very recent related advance, compound **2** and its *N*-*o*-xylyl substituted analogue have also been shown to mediate the cooperative hexamerization of CO in the presence of [Mo(CO)_6_] with formation of magnesium benzenehexolate complexes, [{(BDI)Mg}_6_(C_6_O_6_)].^35^

**Scheme 2:**
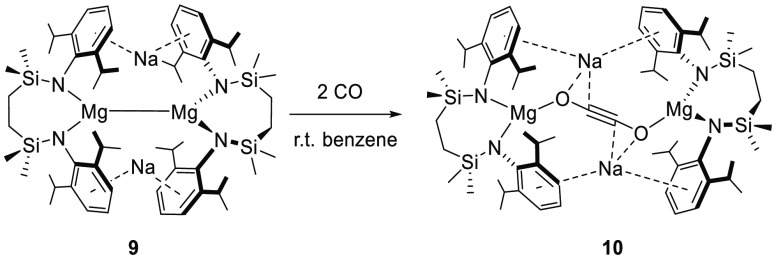
Synthesis of Compound **10**

As an initial assay of its reactivity, therefore, a solution of compound **9** in benzene was treated with 2 atm of ^13^CO at room temperature. The conversion of **9** was complete after 3 days to provide the quantitative generation of a single new species (**10**). The formation of compound **10** was characterized by the loss of asymmetry associated with the {SiN^Dipp^} ligand environments of **9** in the resultant ^1^H NMR spectrum and, most characteristically, the emergence of a single new ^13^C-enriched resonance at δ 50.2 ppm in the corresponding ^13^C{^1^H} NMR spectrum. Although the carbon centers of Jones’ [C_3_O_3_]^2–^ dianions were not observed spectroscopically for comparison,^17^ this latter chemical shift is redolent of Evans’ and co-workers’ observation of the ethynediolate ^13^C nuclei of [{(Me_3_Si)_2_N}_3_Y(μ-OC≡CO)Y{N(SiMe_3_)_2_}_3_][K(18-cr-6)(THF)_2_]_2_ (**11**, δ 55.5 ppm), in which two molecules of CO were reductively dimerized by the Y^2+^ centers produced *in situ* by addition of excess potassium to [Y{N(SiMe_3_)_2_}_3_].^36^ Although analogous behavior has also been observed for similarly generated divalent Lu,^36^ Dy, Ho, Gd, and Tm^37^ systems, and is precedented in uranium(III) reactivity,^38−42^ compound **11** provides the sole diamagnetic example of an ethynediolate anion for comparison and strongly advocates the operation of a similar process during the reaction of CO with **9** (Scheme 2).

**Figure 3:**
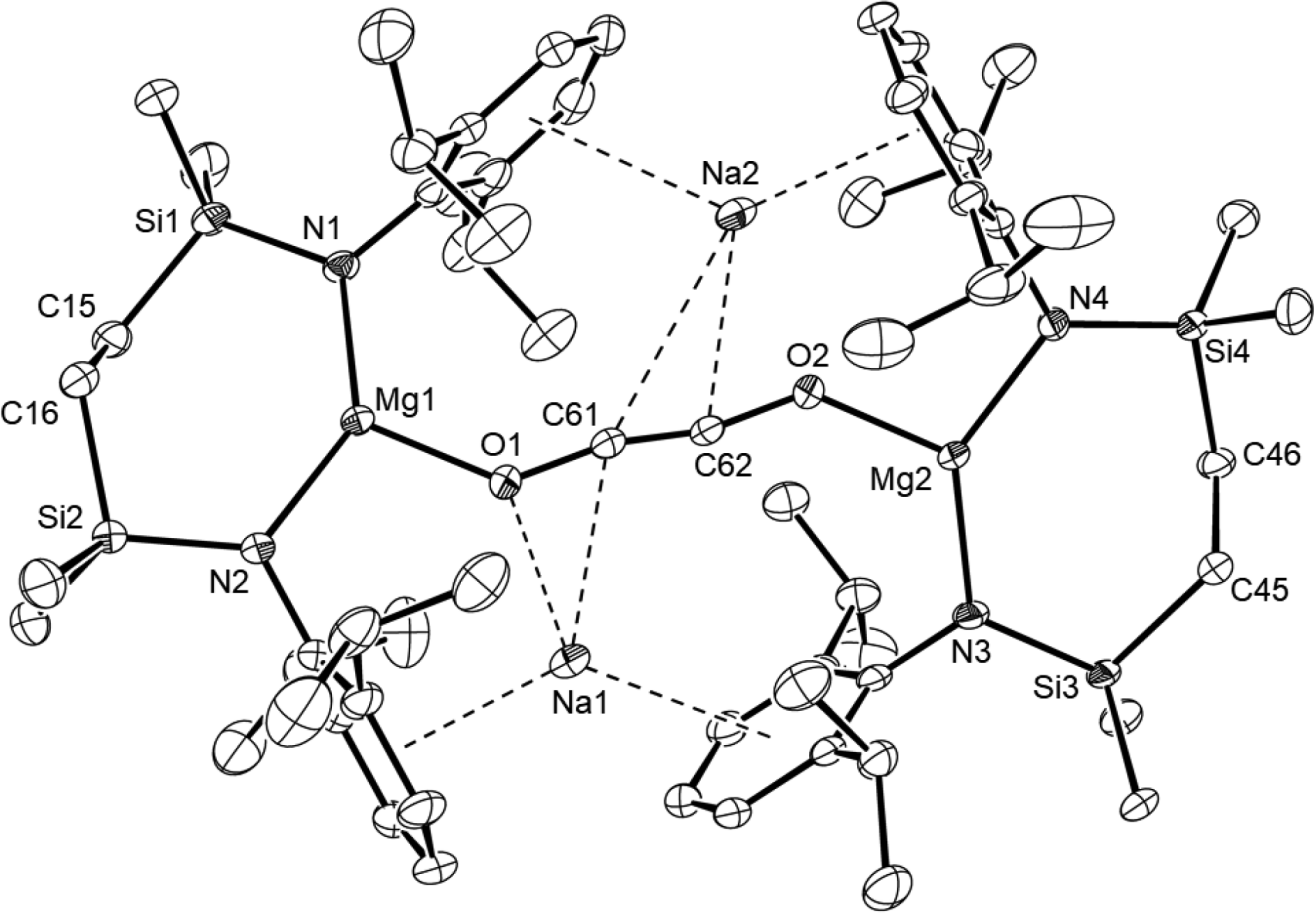
ORTEP (30% probability) of compound **10**. Hydrogen atoms, disordered atoms and disordered molecules of solvent are omitted for clarity. Selected bond lengths (Å) and angles (deg): Mg1–N1 1.9684(11), Mg1–N2 1.9739(12), Mg2–N3 1.9694(12), Mg2–N4 1.9739(13), Mg1–O1 1.8904(11), Mg2–O2 1.8959(10), Na1–O1 2.2477(12), Na1–C61 2.6156(15), Na2–O2 2.3019(11), Na2–C61 3.1107(15), Na2–C62 2.5930(14), C61–C62 1.196(2), N1–Mg1–N2 138.34(5), N3–Mg2–N4 137.29(5), C62–C61–O1 166.82(14), C61–C62–O2 166.62(14).

This thesis was confirmed by the X-ray diffraction analysis of **10**, single crystals of which were obtained by slow evaporation of a hexane solution at room temperature. Although *cis-*enediolate complexes of magnesium have previously been obtained from reactions of β-diketiminato magnesium hydride and magnesium anthracene complexes with CO,^11,43−45^ and Aldridge and co-workers have inferred the intermediacy of ethynediolate species during treatment of a boryl-substituted acyclic silylene with CO,^46,47^ compound **10** (Figure 3) is unique among molecular main group complexes in containing an unperturbed ethynediolate dianion. The structure of **10** reveals that the (μ-OC≡CO) ligand bridges two {SiN^Dipp^}MgNa units in which the magnesium and sodium centers are, respectively, coordinated by the diamide chelate and a series of polyhapto- interactions with the *N-*Dipp substituents. The ethynediolate dianion is bound via terminal Mg–O [Mg1–O1 1.8904(11); Mg2–O2 1.8959(10) Å] and η^3^-C–C–O contacts with the Na1 [Na1–O1 2.2477(12); Na1–C61 2.156(15) Å] and Na2 [Na2–O2 2.3019(11), Na2–C62 2.5930(14) Å] atoms. The extremely short C61–C62 distance of 1.196(2) Å in the ethynediolate moiety is commensurate with that determined by powder X-ray diffraction in NaOC≡CONa [1.19 (0.3) Å].^33^ The C–C triple bond is, thus, unaffected by the proximity of the sodium atoms, albeit the O1–C61–C62 [166.82(14)°] and O2–C62–C61 [166.62(14)°] angles highlight a notable deviation of the {O–C≡C–O} moiety from linearity. Although the Na–C_centroid_ interactions with the Dipp substituents [avg. 2.803 Å] are elongated in comparison to those observed in **9**, more significant is the shortening of the various Mg–N bonds [avg. 1.9714 Å], which supports a change in formal Mg oxidation state from +1 to +2.

This latter deduction was further supported by QTAIM and NBO analyses of the electronic structure and bonding interactions within **10**. QTAIM analysis revealed strong BCPs between each Mg center and the O termini of the {O–C≡C–O}^2–^ moiety (ρ_Mg3–O10_ = 0.0506, ρ_Mg4–O9_ = 0.05182) as well as two between the Na^+^ ions and the O termini (ρ_Na7–O10_ = 0.0232, ρ_Na6–O9_ = 0.0238). Donor-acceptor NBO energy analysis also afforded further support to the η^3^-C–C–O binding mode between the Na^+^ ions and the {O–C≡C–O}^2–^ moiety.

**Figure 4:**
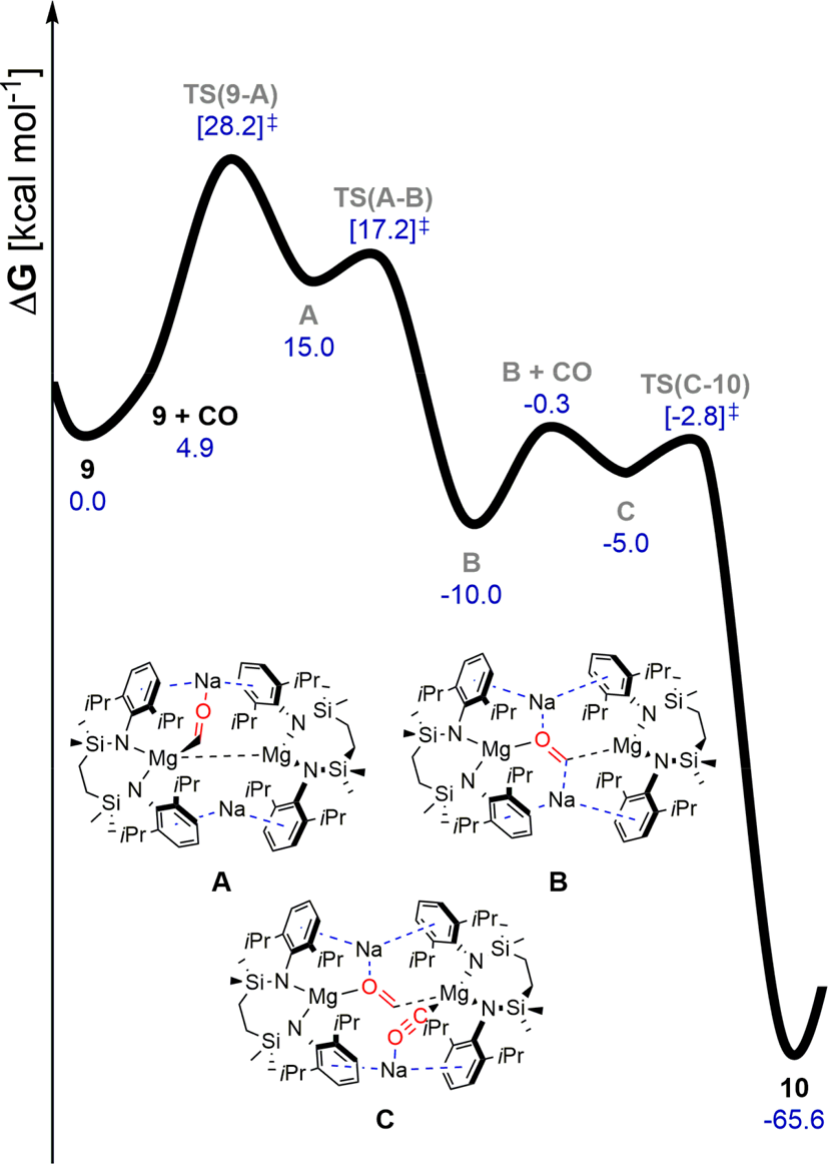
DFT-calculated free energy profile (BP86-D3BJ/BS2(benzene)//BP86/BS1), in kcal mol^–1^ for the reaction of **9** with CO.

The mechanism of formation of **10** was also interrogated by DFT calculations (Figure 4). In a manner reminiscent of that computed for the generation of Jones and Maron’s deltate ([C_3_O_3_]^2–^) derivatives, the addition of CO occurs sequentially.^17,34^ The transformation of **9** to **10** is significantly exergonic (Δ*G* = −65.2 kcal mol^–1^) and each stage of the reaction profile invokes cooperative interactions between CO and both dissimilar metals of the [Na_2_Mg_2_] unit. Consistent with the slow formation of **10**, the kinetic barrier associated with the initial CO insertion [TS(**9**-**A**)], involving a single Mg and a single Na of **9** and the generation of a Mg−μ-C–O−μ-Na bridging interaction, is quite high and rate limiting (28.2 kcal mol^–1^). Although the formation of the resultant species (**A**) is endergonic (Δ*G* = +15.0 kcal mol^–1^), the overall exergonicity of CO insertion (Δ*G* = −10.0 kcal mol^–1^) is ensured by its rapid isomerization to species **B**, in which a doubly reduced molecule of CO interacts with all four alkaline metal centers. Sequestration of a further molecule of CO by **B** to provide **C** and subsequent C–C coupling are then facile to provide **10**, in which the (μ-OC≡CO) unit is encapsulated and protected toward further oligomerization by the [Na_2_Mg_2_]^2+^ unit.

In summary, sodium reduction of a neutral diamidomagnesium provides a Mg(I) species in which the long Mg–Mg interaction is augmented by persistent Na–aryl interactions. Computational assessment indicates that the molecule is best considered to comprise a contiguous tetrametallic core, a viewpoint borne out by its reaction with CO, which results in ethynediolate formation mediated by the dissimilar metal centers. We are continuing to study this reactivity, particularly as a strategy to allow the isolation of low oxidation state derivatives of magnesium’s heavier group 2 congeners.

## Supporting Information Available

General synthetic experimental details; NMR spectra; X-ray analysis of compounds **8**·benzene, **8**·toluene, **9**, and **10** (CCDC 2107893–2107896); details of the computational analysis and atomic coordinates (PDF)

## Accession Codes

CCDC 2107893–2107896 contain the supplementary crystallographic data for this paper. These data can be obtained free of charge via www.ccdc.cam.ac.uk/data_request/cif, or by emailing data_request@ccdc.cam.ac.uk, or by contacting The Cambridge Crystallographic Data Centre, 12 Union Road, Cambridge CB2 1EZ, UK; fax: +44 1223 336033.

## References

[ref1] Green S. P.; Jones C.; Stasch A. Stable magnesium(I) compounds with Mg-Mg bonds. Science 2007, 318 (5857), 1754–1757. 10.1126/science.1150856. 17991827

[ref2] Stasch A.; Jones C. Stable dimeric magnesium(I) compounds: from chemical landmarks to versatile reagents. Dalton Trans. 2011, 40 (21), 5659–5672. 10.1039/c0dt01831g. 21390353

[ref3] Jones C.; Stasch A. Stable Molecular Magnesium(I) Dimers: A Fundamentally Appealing Yet Synthetically Versatile Compound Class. Top. Organomet. Chem. 2013, 45, 73–101. 10.1007/978-3-642-36270-5_3.

[ref4] Jones C. Dimeric magnesium(I) beta-diketiminates: a new class of quasi-universal reducing agent. Nat. Rev. Chem. 2017, 1 (8), 0059 10.1038/s41570-017-0059.

[ref5] aJones C. Open questions in low oxidation state group 2 chemistry. Commun. Chem. 2020, 3 (1), 159 10.1038/s42004-020-00408-8. PMC9814366 36703461

[ref6] Wang X. F.; Andrews L. Infrared spectra of magnesium hydride molecules, complexes, and solid magnesium dihydride. J. Phys. Chem. A 2004, 108 (52), 11511–11520. 10.1021/jp046410h.

[ref7] Green S. P.; Jones C.; Stasch A. Stable Adducts of a Dimeric Magnesium(I) Compound. Angew. Chem., Int. Ed. 2008, 47 (47), 9079–9083. 10.1002/anie.200803960. 18925599

[ref8] Bonyhady S. J.; Jones C.; Nembenna S.; Stasch A.; Edwards A. J.; McIntyre G. J. β-Diketiminate-Stabilized Magnesium(I) Dimers and Magnesium(II) Hydride Complexes: Synthesis, Characterization, Adduct Formation, and Reactivity Studies. Chem. - Eur. J. 2010, 16 (3), 938–955. 10.1002/chem.200902425. 19950340

[ref9] Overgaard J.; Jones C.; Stasch A.; Iversen B. B. Experimental Electron Density Study of the Mg-Mg Bonding Character in a Magnesium(I) Dimer. J. Am. Chem. Soc. 2009, 131 (12), 4208 10.1021/ja900385u. 19278225

[ref10] Liu Y.; Li S.; Yang X.-J.; Yang P.; Wu B. Magnesium-Magnesium Bond Stabilized by a Doubly Reduced alpha-Diimine: Synthesis and Structure of {K(THF)_3_}_2_LMg-MgL (L = (2,6-{(iPr_2_C_6_H_3_NC(Me)}_2_^2-^. J. Am. Chem. Soc. 2009, 131 (12), 4210–4211. 10.1021/ja900568c. 19271703

[ref11] Lalrempuia R.; Kefalidis C. E.; Bonyhady S. J.; Schwarze B.; Maron L.; Stasch A.; Jones C. Activation of CO by Hydrogenated Magnesium(I) Dimers: Sterically Controlled Formation of Ethenediolate and Cyclopropanetriolate Complexes. J. Am. Chem. Soc. 2015, 137 (28), 8944–8947. 10.1021/jacs.5b06439. 26135846

[ref12] Li J.; Luo M.; Sheng X. C.; Hua H. M.; Yao W. W.; Pullarkat S. A.; Xu L.; Ma M. T. Unsymmetrical β-diketiminate magnesium(I) complexes: syntheses and application in catalytic hydroboration of alkyne, nitrile and carbonyl compounds. Org. Chem. Front. 2018, 5 (24), 3538–3547. 10.1039/C8QO00720A.

[ref13] Pernik I.; Maitland B. J.; Stasch A.; Jones C. Synthesis and attempted reductions of bulky 1,3,5-triazapentadienyl groups 2 and 13 halide complexes. Can. J. Chem. 2018, 96 (6), 513–521. 10.1139/cjc-2017-0548.

[ref14] Ma M. M.; Wang H. H.; Wang J. J.; Shen L. Y.; Zhao Y. X.; Xu W. H.; Wu B.; Yang X. J. Mg-Mg-bonded compounds with *N*,*N*-dipp-substituted phenanthrene-diamido and *o*-phenylene-diamino ligands. Dalton Trans. 2019, 48 (7), 2295–2299. 10.1039/C9DT00028C. 30681693

[ref15] Gentner T. X.; Rösch B.; Ballmann G.; Langer J.; Elsen H.; Harder S. Low Valent Magnesium Chemistry with a Super Bulky β-Diketiminate Ligand. Angew. Chem., Int. Ed. 2019, 58 (2), 607–611. 10.1002/anie.201812051. 30422354

[ref16] Boutland A. J.; Dange D.; Stasch A.; Maron L.; Jones C. Two-Coordinate Magnesium(I) Dimers Stabilized by Super Bulky Amido Ligands. Angew. Chem., Int. Ed. 2016, 55 (32), 9239–9243. 10.1002/anie.201604362. 27303934

[ref17] Yuvaraj K.; Douair I.; Paparo A.; Maron L.; Jones C. Reductive Trimerization of CO to the Deltate Dianion Using Activated Magnesium(I) Compounds. J. Am. Chem. Soc. 2019, 141 (22), 8764–8768. 10.1021/jacs.9b04085. 31096751

[ref18] Rösch B.; Gentner T. X.; Eyselein J.; Friedrich A.; Langer J.; Harder S. Mg-Mg bond polarization induced by a superbulky β-diketiminate ligand. Chem. Commun. 2020, 56 (77), 11402–11405. 10.1039/D0CC05200K. 32852001

[ref19] Platts J. A.; Overgaard J.; Jones C.; Iversen B. B.; Stasch A. First Experimental Characterization of a Non-nuclear Attractor in a Dimeric Magnesium(I) Compound. J. Phys. Chem. A 2011, 115 (2), 194–200. 10.1021/jp109547w. 21158464

[ref20] Wu L. C.; Jones C.; Stasch A.; Platts J. A.; Overgaard J. Non-Nuclear Attractor in a Molecular Compound under External Pressure. Eur. J. Inorg. Chem. 2014, 2014 (32), 5536–5540. 10.1002/ejic.201402606.

[ref21] Stasch A. Synthesis of a Dimeric Magnesium(I) Compound by an Mg-I/Mg-II Redox Reaction. Angew. Chem., Int. Ed. 2014, 53 (38), 10200–10203. 10.1002/anie.201404284. 25047459

[ref22] Bakewell C.; White A. J. P.; Crimmin M. R. Addition of Carbon-Fluorine Bonds to a Mg(I)-Mg(I) Bond: An Equivalent of Grignard Formation in Solution. J. Am. Chem. Soc. 2016, 138 (39), 12763–12766. 10.1021/jacs.6b08104. 27636244 PMC5135227

[ref23] Rosch B.; Gentner T. X.; Eyselein J.; Langer J.; Elsen H.; Harder S. Strongly reducing magnesium(0) complexes. Nature 2021, 592 (7856), 717–719. 10.1038/s41586-021-03401-w. 33911274

[ref24] Shannon R. D. Revised effective ionic radii and systematic studies of interatomic distances in halides and chalcogenides. Acta Crystallogr., Sect. A: Cryst. Phys., Diffr., Theor. Gen. Crystallogr. 1976, 32 (SEP1), 751–767. 10.1107/S0567739476001551.

[ref25] aHicks J.; Vasko P.; Goicoechea J. M.; Aldridge S. Synthesis, structure and reaction chemistry of a nucleophilic aluminyl anion. Nature 2018, 557 (7703), 92–95. 10.1038/s41586-018-0037-y. 29662211

[ref26] aSchwamm R. J.; Anker M. D.; Lein M.; Coles M. P. Reduction vs. Addition: The Reaction of an Aluminyl Anion with 1,3,5,7-Cyclooctatetraene. Angew. Chem., Int. Ed. 2019, 58 (5), 1489–1493. 10.1002/anie.201811675. 30548141

[ref27] Grams S.; Eyselein J.; Langer J.; Farber C.; Harder S. Boosting Low-Valent Aluminum(I) Reactivity with a Potassium Reagent. Angew. Chem., Int. Ed. 2020, 59 (37), 15982–15986. 10.1002/anie.202006693. PMC7540686 32449816

[ref28] Schwamm R. J.; Coles M. P.; Hill M. S.; Mahon M. F.; McMullin C. L.; Rajabi N. A.; Wilson A. S. S. A Stable Calcium Alumanyl. Angew. Chem., Int. Ed. 2020, 59 (10), 3928–3932. 10.1002/anie.201914986. PMC7159655 31830364

[ref29] Gentner T. X.; Mulvey R. E. Alkali-Metal Mediation: Diversity of Applications in Main-Group Organometallic Chemistry. Angew. Chem., Int. Ed. 2021, 60 (17), 9247–9262. 10.1002/anie.202010963. PMC8247348 33017511

[ref30] Pardue D. B.; Gustafson S. J.; Periana R. A.; Ess D. H.; Cundari T. R. Computational study of carbon-hydrogen bond deprotonation by alkali metal superbases. Comput. Theor. Chem. 2013, 1019, 85–93. 10.1016/j.comptc.2013.06.041.

[ref31] Hicks J.; Juckel M.; Paparo A.; Dange D.; Jones C. Multigram Syntheses of Magnesium(I) Compounds Using Alkali Metal Halide Supported Alkali Metals as Dispersible Reducing Agents. Organometallics 2018, 37 (24), 4810–4813. 10.1021/acs.organomet.8b00803.

[ref32] Buchner W.; Weiss E. Zur kenntnis sogenannten alkalicarbonyle 4 uber reaction von geschmolzenem kalium mit kohlenmonoxid. Helv. Chim. Acta 1964, 47, 1415 10.1002/hlca.19640470604.

[ref33] Weiss E.; Buchner W. Zure Zur kenntnis sogenannten alkalicarbonyle V. Die Kristallstrukttur des natriumacetylendiolats. Chem. Ber. 1965, 98, 126 10.1002/cber.19650980116.

[ref34] Yuvaraj K.; Douair I.; Jones D. D. L.; Maron L.; Jones C. Sterically controlled reductive oligomerisations of CO by activated magnesium(I) compounds: deltate vs. ethenediolate formation. Chem. Sci. 2020, 11 (13), 3516–3522. 10.1039/D0SC00836B. 34109023 PMC8152598

[ref35] Paparo A.; Yuvaraj K.; Matthews A. J. R.; Douair I.; Maron L.; Jones C. Reductive Hexamerization of CO Involving Cooperativity Between Magnesium(I) Reductants and Mo(CO)_6_: Synthesis of Well-Defined Magnesium Benzenehexolate Complexes. Angew. Chem., Int. Ed. 2021, 60 (2), 630–634. 10.1002/anie.202009523. 32969564

[ref36] Fang M.; Farnaby J. H.; Ziller J. W.; Bates J. E.; Furche F.; Evans W. J. Isolation of (CO)^1-^ and (CO)_2_^1-^ Radical Complexes of Rare Earths via Ln(NR_2_)_3_/K Reduction and K_2_(18-crown-6)_2_^2+^ Oligomerization. J. Am. Chem. Soc. 2012, 134 (14), 6064–6067. 10.1021/ja211220r. 22435647

[ref37] Ryan A. J. J.; Ziller J. W.; Evans W. J. The importance of the counter-cation in reductive rare-earth metal chemistry: 18-crown-6 instead of 2,2,2-cryptand allows isolation of Y-II(NR_2_)_3_^1-^ and ynediolate and enediolate complexes from CO reactions. Chemical Science 2020, 11 (7), 2006–2014. 10.1039/C9SC05794C. 34123296 PMC8150099

[ref38] Frey A. S.; Cloke F. G. N.; Hitchcock P. B.; Day I. J.; Green J. C.; Aitken G. Mechanistic studies on the reductive cyclooligomerisation of CO by U(III) mixed sandwich complexes; the molecular structure of (U(C_8_H_6_){Si-*i*-Pr_3_)-1,4}_2_)(Cp*)_2_((-C_2_O_2_). J. Am. Chem. Soc. 2008, 130 (42), 13816–13817. 10.1021/ja8059792. 18817397

[ref39] Arnold P. L.; Turner Z. R.; Bellabarba R. M.; Tooze R. P. Carbon monoxide coupling and functionalisation at a simple uranium coordination complex. Chem. Sci. 2011, 2 (1), 77–79. 10.1039/C0SC00452A.

[ref40] Mansell S. M.; Kaltsoyannis N.; Arnold P. L. Small Molecule Activation by Uranium Tris(aryloxides): Experimental and Computational Studies of Binding of N_2_, Coupling of CO, and Deoxygenation Insertion of CO_2_ under Ambient Conditions. J. Am. Chem. Soc. 2011, 133 (23), 9036–9051. 10.1021/ja2019492. 21591662

[ref41] Gardner B. M.; Stewart J. C.; Davis A. L.; McMaster J.; Lewis W.; Blake A. J.; Liddle S. T. Homologation and functionalization of carbon monoxide by a recyclable uranium complex. Proc. Natl. Acad. Sci. U. S. A. 2012, 109 (24), 9265–9270. 10.1073/pnas.1203417109. 22652572 PMC3386139

[ref42] Tsoureas N.; Summerscales O. T.; Cloke F. G. N.; Roe S. M. Steric Effects in the Reductive Coupling of CO by Mixed-Sandwich Uranium(III) Complexes. Organometallics 2013, 32 (5), 1353–1362. 10.1021/om301045k.

[ref43] Anker M. D.; Hill M. S.; Lowe J. P.; Mahon M. F. Alkaline-Earth-Promoted CO Homologation and Reductive Catalysis. Angew. Chem., Int. Ed. 2015, 54 (34), 10009–10011. 10.1002/anie.201505851. PMC4678424 26220407

[ref44] Anker M. D.; Kefalidis C. E.; Yang Y.; Fang J.; Hill M. S.; Mahon M. F.; Maron L. Alkaline Earth-Centered CO Homologation, Reduction, and Amine Carbonylation. J. Am. Chem. Soc. 2017, 139 (29), 10036–10054. 10.1021/jacs.7b04926. 28640639

[ref45] Yuvaraj K.; Jones C. Reductive coupling of CO with magnesium anthracene complexes: formation of magnesium enediolates. Chem. Commun. 2021, 57 (73), 9224–9227. 10.1039/D1CC03890G. 34519307

[ref46] Crimmin M. R.; Kong R. Y.; Phillips N., Coordination and Activation of Alkanes, CO and CO_2_ at Metal Centres. Reference Module in Chemistry, Molecular Sciences and Chemical Engineering; Elsevier: 2021; pp 1–52.

[ref47] Protchenko A. V.; Vasko P.; Do D. C. H.; Hicks J.; Fuentes M. A.; Jones C.; Aldridge S. Reduction of Carbon Oxides by an Acyclic Silylene: Reductive Coupling of CO. Angew. Chem., Int. Ed. 2019, 58 (6), 1808–1812. 10.1002/anie.201812675. 30537262

