# Supplementary material for: Reductive Dimerization of CO by a Na/Mg(I) Diamide
Source: J Am Chem Soc. 2021 Oct 15;143(42):17851–6. doi: 10.1021/jacs.1c09467 (PMC8554760; doi:10.1021/jacs.1c09467)
Supplement: Supplementary file 1 — ja1c09467_si_001.pdf [file ja1c09467_si_001.pdf]

Experimental and Supplementary Information for

Reductive Dimerization of CO by a Na/Mg(I) diamide

Han-Ying Liu, Ryan J. Schwamm, Samuel E. Neale, Michael S. Hill, Claire L. McMullin and Mary F. Mahon

Department of Chemistry, University of Bath, Claverton Down, Bath, BA2 7AY, UK

Table of Contents

General information 1

Synthetic Procedures 2

Synthesis of $\{SiN^{Dipp}\}Mg$ (**8**) 2

Synthesis of $[\{SiN^{Dipp}\}MgNa]_2$ (**9**) 4

Synthesis of $[\{SiN^{Dipp}\}Mg(CO)Na]_2$ (**10**) 8

Single Crystal X-ray Diffraction Analysis 10

Table S1: Single crystal X-ray diffraction analysis of compounds **8**·benzene**, 8**·toluene, **9** and **10**. 11

Computational Details 13

Relative Energies and Mechanism Discussion 14

QTAIM 27

NBO 20

References 25

Cartesian Coordinates and Computed Energies for Calculated Structures 26

General information

Unless stated otherwise, all the experiments were conducted using standard Schlenk line and/or glovebox techniques under an inert atmosphere of argon. NMR spectra were recorded with an Agilent ProPulse spectrometer (^{1}H at 500 MHz, ^{13}C at 126 MHz). The spectra are referenced relative to residual protio solvent resonances. Elemental analyses were performed at Elemental Microanalysis Ltd., Okehampton, Devon, UK. Solvents were dried by passage through a commercially available solvent purification system and stored under argon in ampoules over 4 Å molecular sieves. C_6D_6 was purchased from Sigma-Aldrich, dried over a potassium mirror before distilling and storage over molecular sieves. $\{SiN^{Dipp}\}H_2$ was prepared according to reported procedure,[1] all other chemicals were purchased from Merck and used without further purification.

Synthetic Procedures

Synthesis of {SiN^{Dipp}}Mg (**8**)

A heptane solution of $MgBu_2$ (1.0M, 16mL, 16mmol) was added dropwise to a solution of {SiN^{Dipp}}H_2 (8.0 g, 16 mmol) in hexane (50 mL) at room temperature. The reaction mixture was then left stirring at room temperature for 18 hours under a weak flow of argon, yielding a clear solution with a colorless precipitate. The hexane solution was then removed by filtration, and the residual colorless solid was collected, followed by removal of all volatiles under vacuum, giving the title compound as a colorless powder. Yield 7.8 g, 94%. Anal Calc'd for $C_{30}H_{50}MgN_2Si_2$ (**8**, 519.22): C, 69.40; H, 9.71; N, 5.40 %. Found: C, 69.24; H, 9.69; N, 5.25 %. Compound **8** quickly isomerizes in the presence of the aromatic solvent, forming the **8**·benzene or **8**·toluene molecules. Single crystals suitable for X-ray crystallography were obtained as colorless blocks from slow evaporation of the respective arene solution. ^{1}H NMR (500 MHz, 298K, Benzene-d_6) δ 7.12 – 7.03 (m, 4H, *m*-C_6H_3), 7.05 – 6.97 (m, 2H, , *p*-C_6H_3), 3.87 (sept, J = 6.9 Hz, 4H, C*H*Me$_2$), 1.20 (d, J = 6.9 Hz, 24H, CH*Me$_2$*), 1.13 (s, 4H, SiC*H$_2$*), 0.26 (s, 12H, Si*Me$_2$*). ^{13}C NMR (126 MHz, 298K, Benzene-d_6) δ 144.2 (*i*-C_6H_3), 130.6 (*o*-C_6H$_3$), 123.8 (*m*-C_6H$_3$), 120.9 (*p*-C_6H$_3$), 27.7 (*C*HMe$_2$), 25.0 (CH*Me$_2$*), 12.7 (Si*C*H$_2$), 1.1 (Si*Me$_2$*).

Figure S1. ^{1}H NMR Spectrum of **8**·toluene (500 MHz, C_6D_6); # toluene.

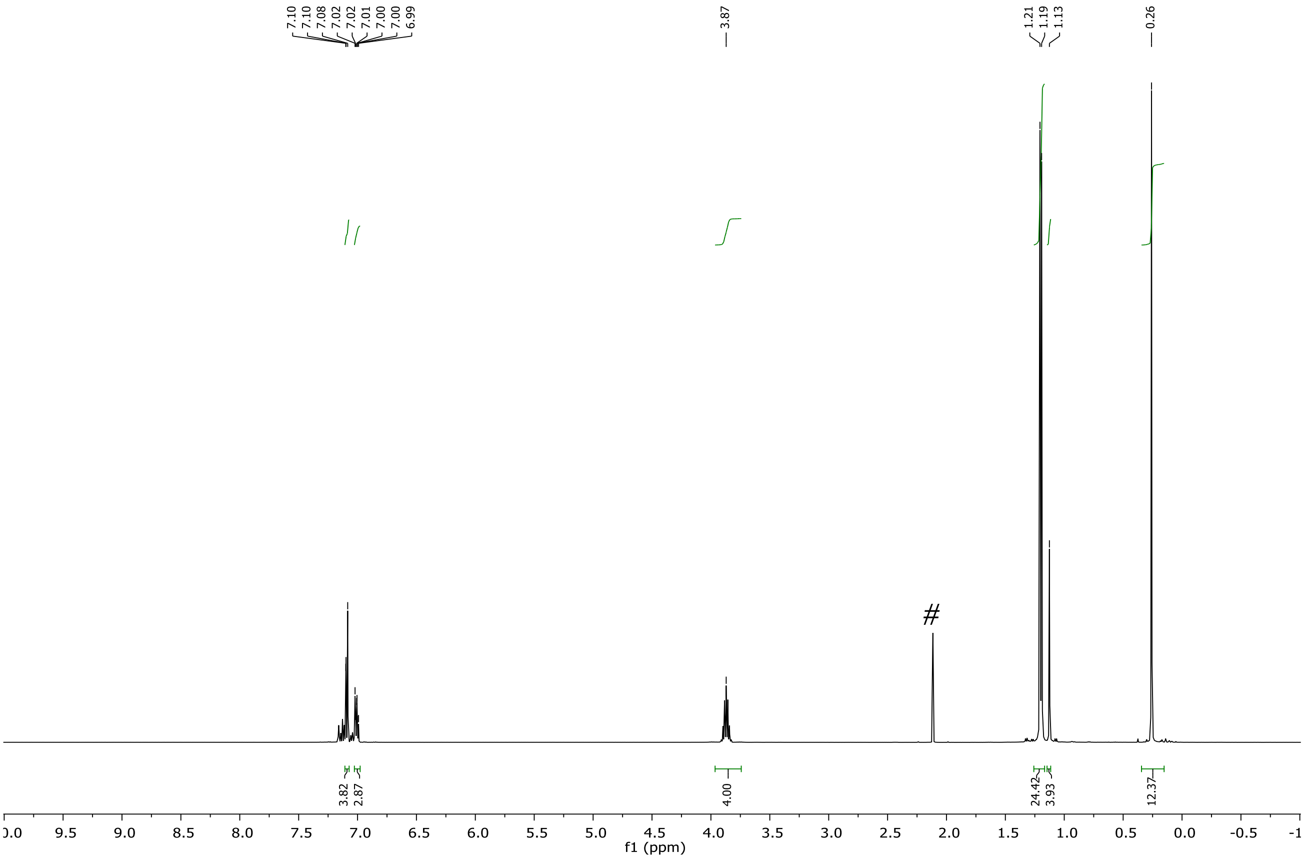

Figure S2. $^{13}C\{^{1}H\}$ NMR Spectrum of **8**·toluene (126 MHz, C_6D_6).

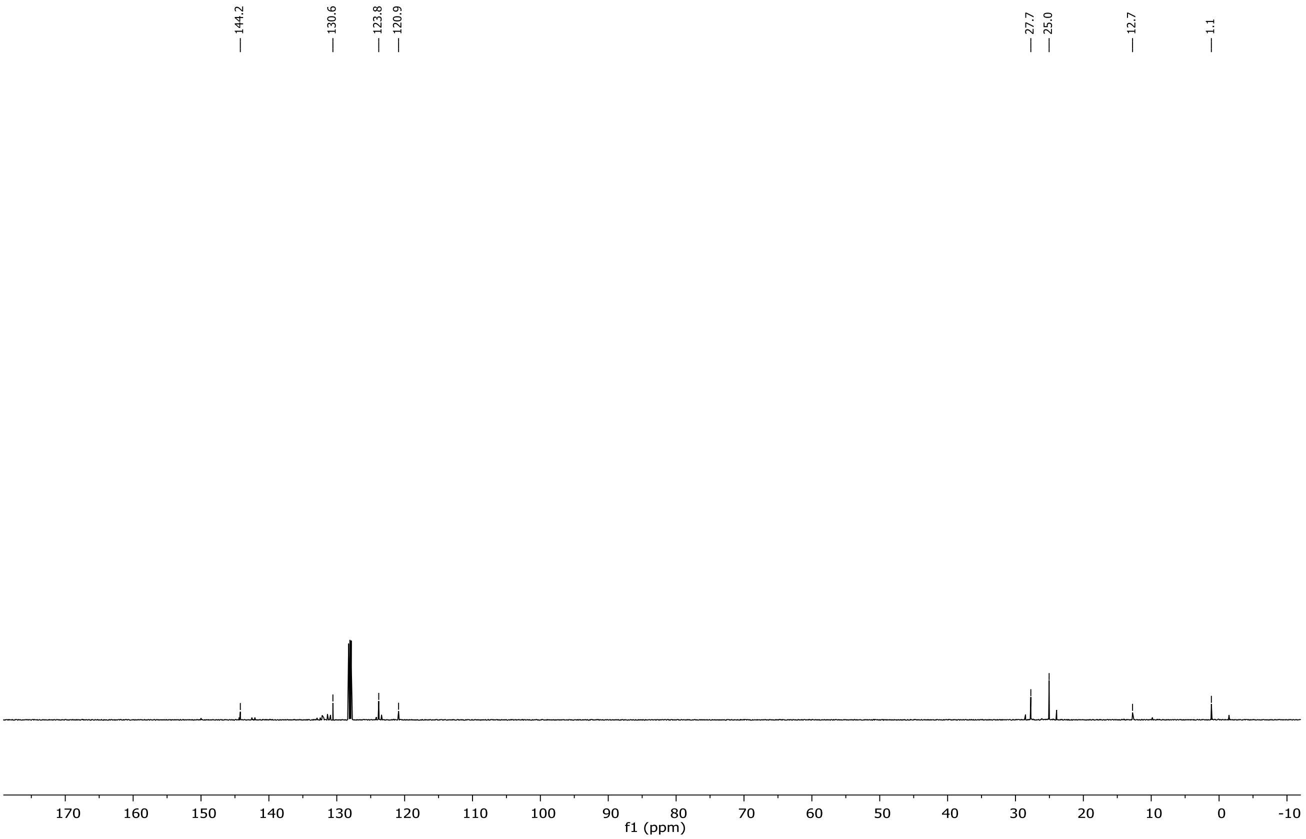

Synthesis of $[\{SiN^{Dipp}\}MgNa]_2$ (**9**)

$\{SiN^{Dipp}\}_2Mg$ (**8**) (0.5 g, 0.987 mmol) and 5 wt.% Na/NaCl (1.4 g, 3.04 mmol) was charged into a Schlenk, followed by addition of benzene (40 mL) via cannula at room temperature. The reaction mixture was then stirred for 12 hours, yielding a yellow solution with grey suspension. All the solid was then filtered, and all volatiles were then removed under vacuum. Toluene (5 mL) was then added to give a yellow solution which was kept at –30°C, yielding bright yellow crystals of **9**. Yield 0.384 g, 72%. Anal Calc'd for $C_{67}H_{108}Mg_2Na_2N_4Si_4$ (**9**.C_7H_8, 1176.56): C, 68.40; H, 9.25; N, 4.76 %. Found: C, 68.20; H, 9.03; N, 4.71 %. ^{1}H NMR (500 MHz, 298K, Benzene-d_6) δ 6.89 (d, J = 8.8 Hz, 2H, *m*-C_6H_3), 6.81 (d, J = 8.8 Hz, 2H, *m*-C_6H_3), 6.53-6.45 (m, 2H, *p*-C_6H_3), 4.41 (sept, J = 6.9 Hz, 2H, C*H*Me_2), 3.91 (sept, J = 6.9 Hz, 2H, C*H*Me_2), 1.38 (d, J = 6.9 Hz, 6H, CH*Me_2*), 1.28 (d, J = 6.9 Hz, 4H, CH*Me_2*), 1.24 (s, 2H, SiC*H_2*), 1.22 (s, 2H, SiC*H_2*), 1.17 (d, J = 6.9 Hz, 12H, CH*Me_2*, overlapping), 0.52 (s, 6H, Si*Me_2*), -0.21 (s, 6H, Si*Me_2*). ^{13}C NMR (126 MHz, 298K, Benzene-d_6) δ 156.6 (*i*-*C*$_6$H$_3$), 148.3 (*o*-*C*$_6$H$_3$), 147.06 (*o*-*C*$_6$H$_3$), 125.0 (*m*-*C*$_6$H$_3$), 123.5 (*m*-*C*$_6$H$_3$), 118.2 (*p*-*C*$_6$H$_3$), 28.4 (CH*Me_2*), 27.4 (*C*HMe_2), 27.04 (CH*Me_2*), 26.48 (*C*HMe_2), 25.1 (CH*Me_2*), 25.1 (CH*Me_2*), 23.9 (Si*C*H_2), 4.0 (Si*Me_2*), 1.9 (Si*Me_2*). *Toluene impurity observed.

Figure S3. ^{1}H NMR Spectrum of ***9*** (500 MHz, C_6D_6); #toluene.

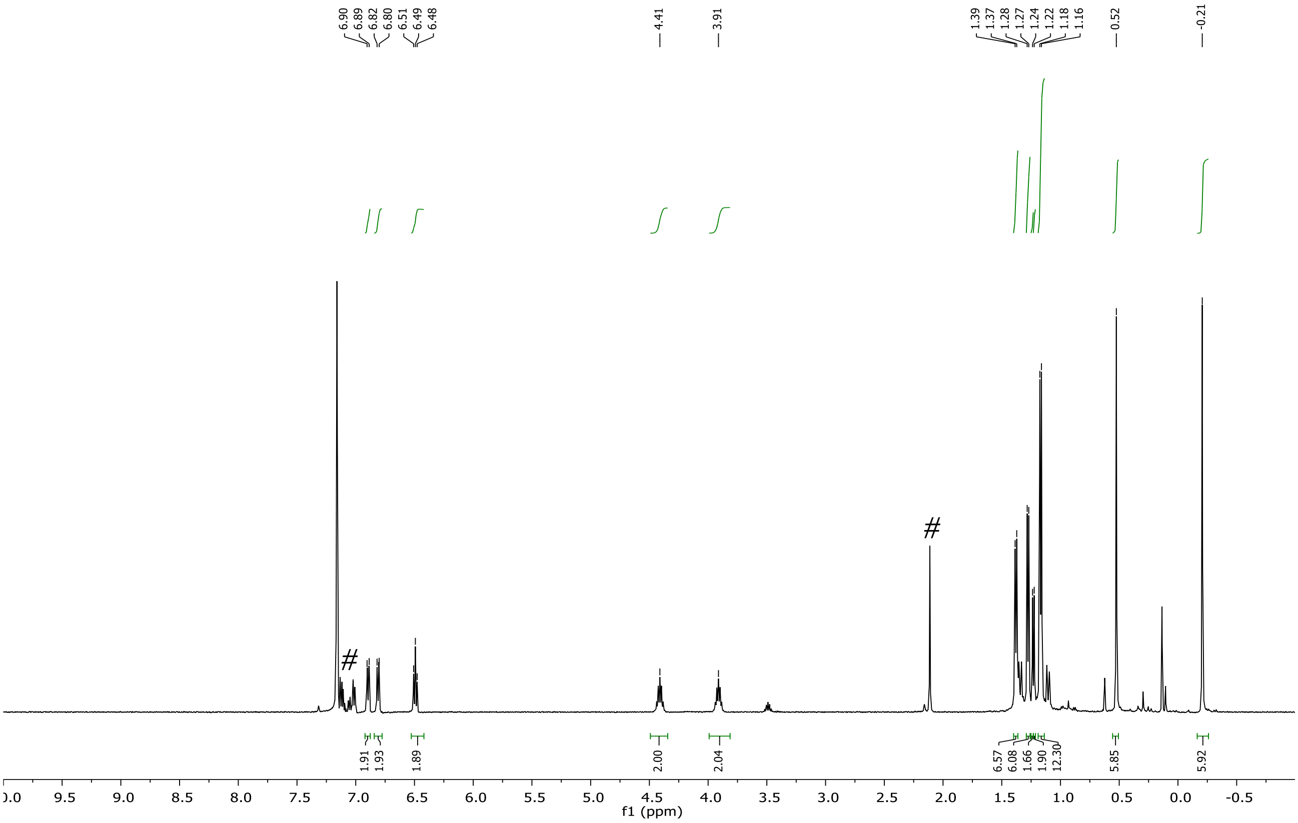

Figure S4. ^{13}C{^{1}H} NMR Spectrum of **9** (126 MHz, C_6D_6).

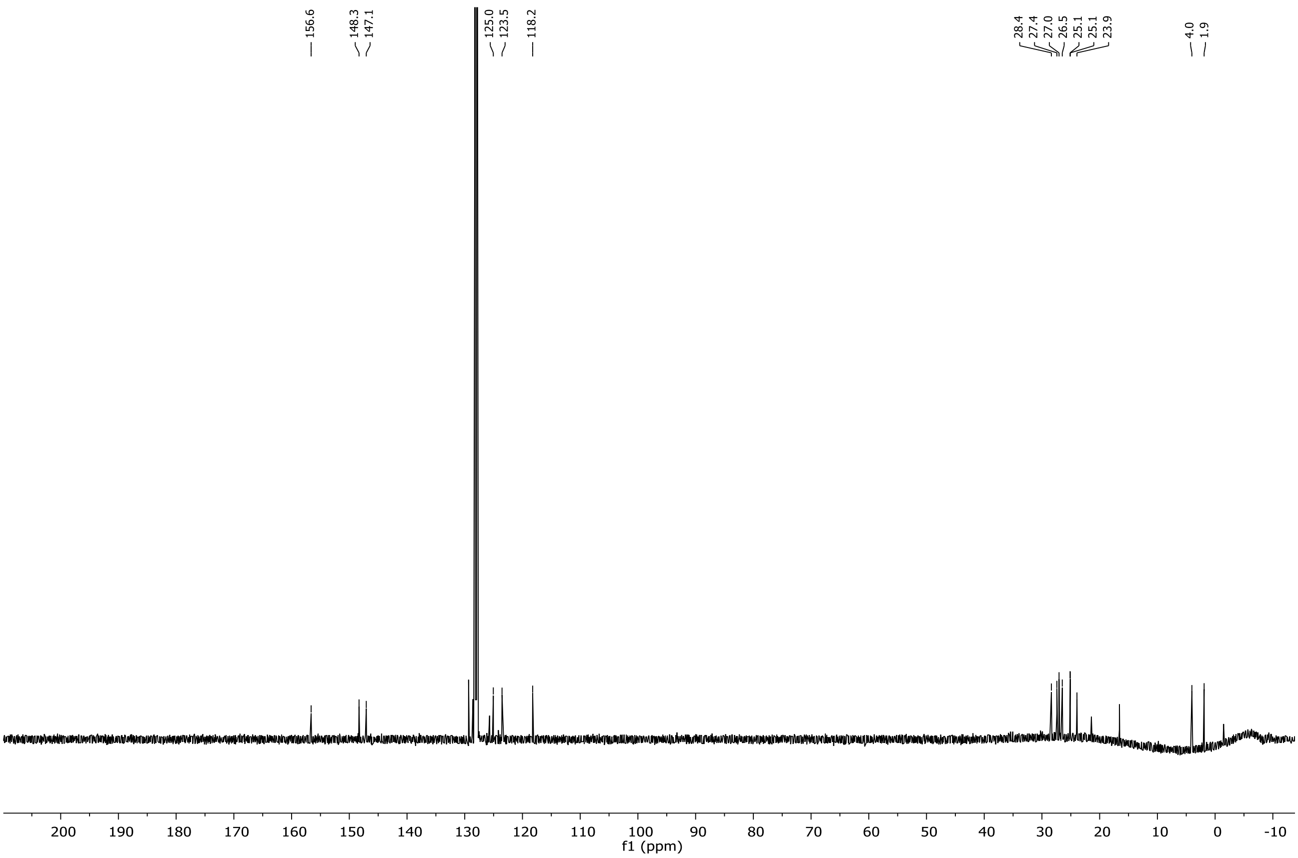

Figure S5. ^{1}H-^{1}H COSY NMR Spectrum of **9**.

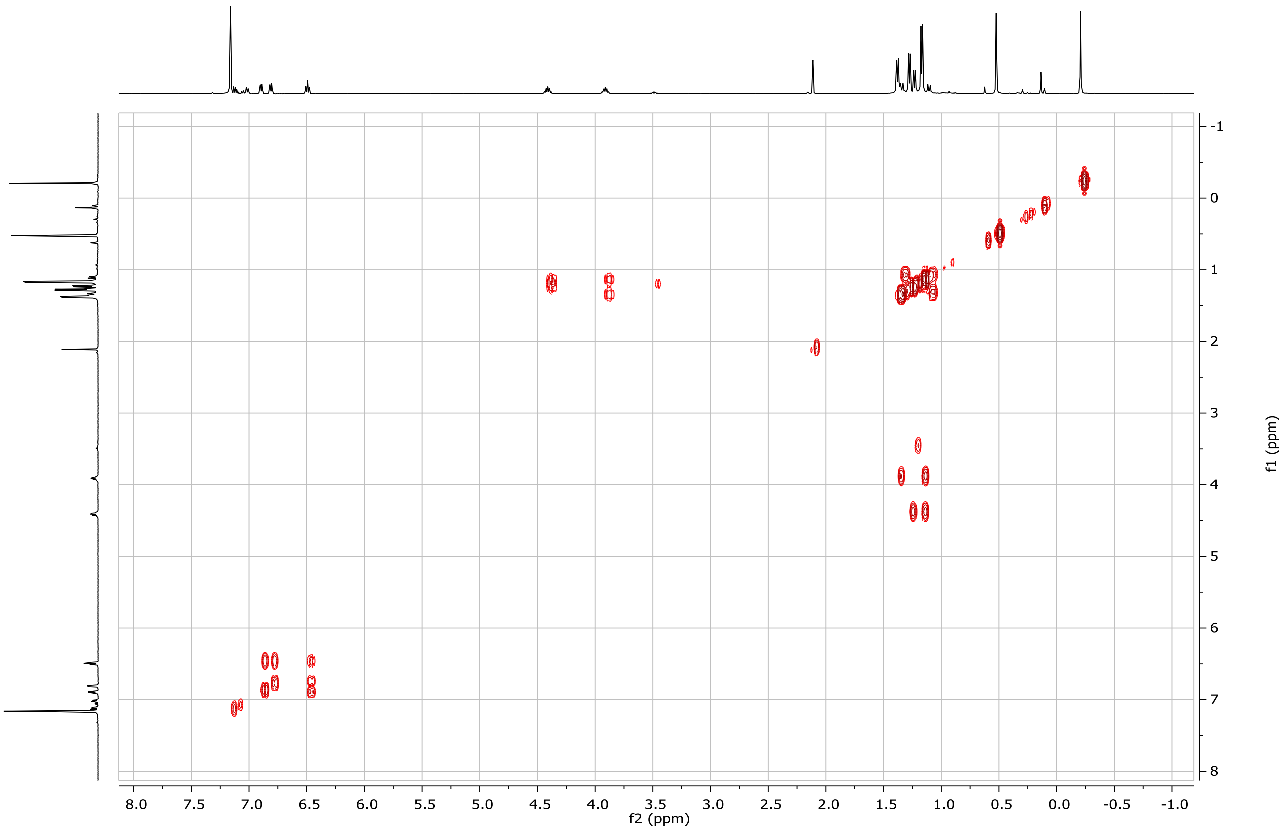

Figure S6. ^{1}H-^{1}H NOESY EXSY NMR Spectrum of **9**.

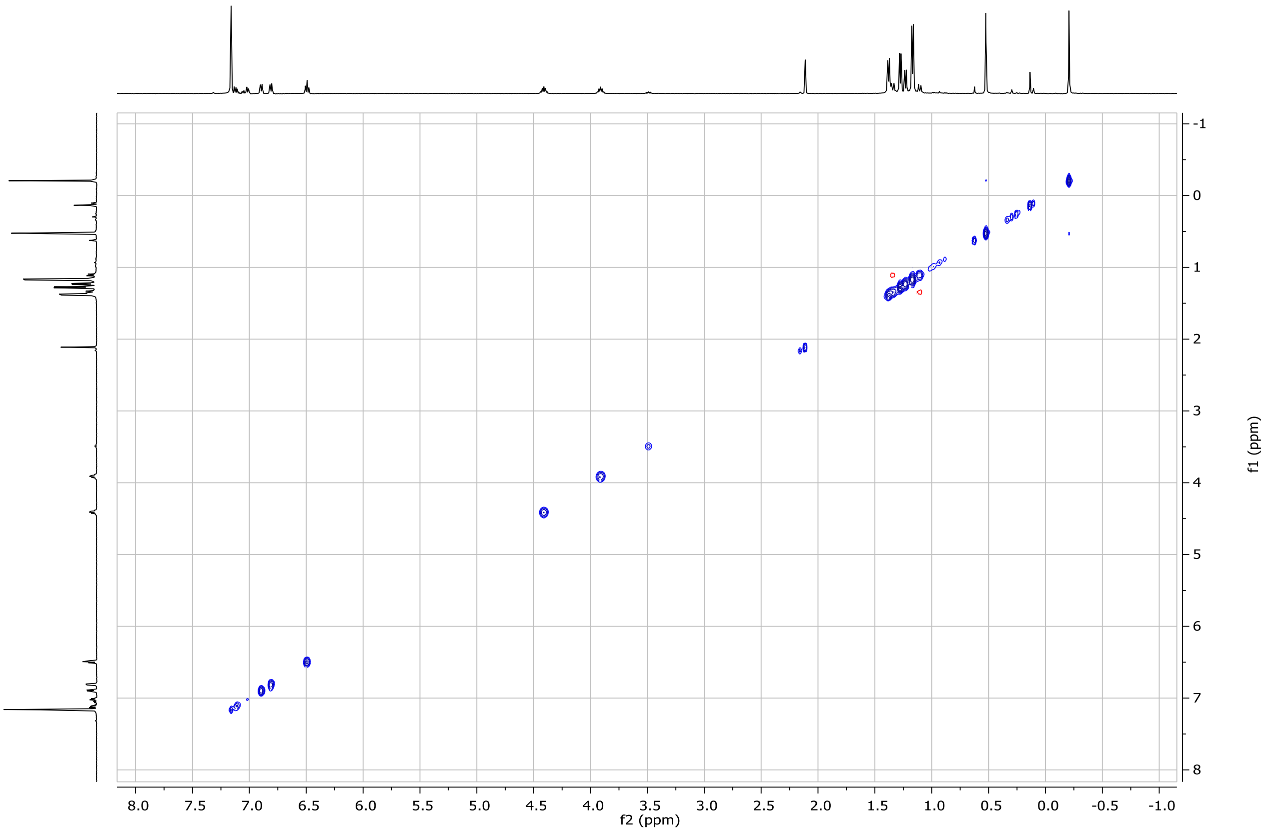

Figure S7. ¹H-¹³C HSQC NMR Spectrum of **9**.

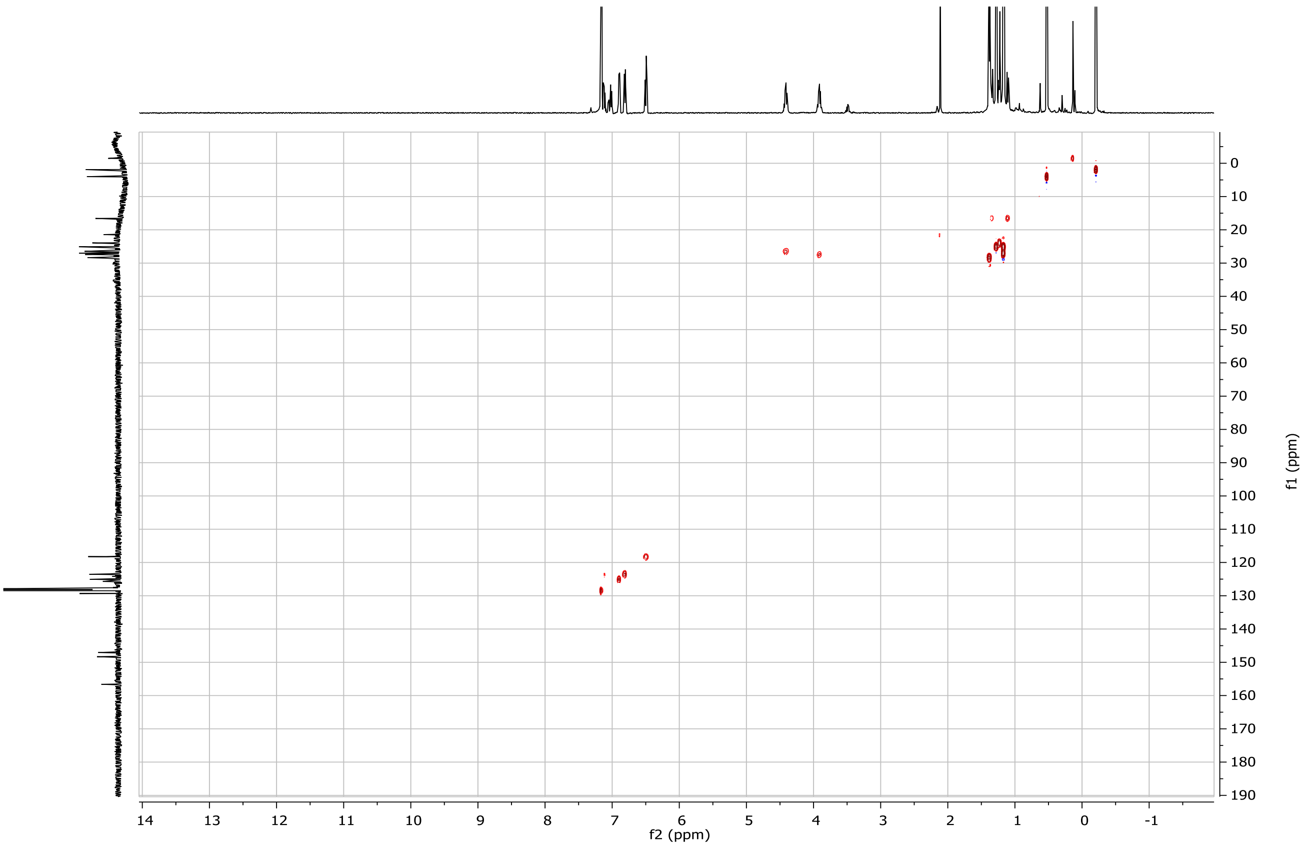

Figure S8. ¹H-¹³C HMBC NMR Spectrum of **9**.

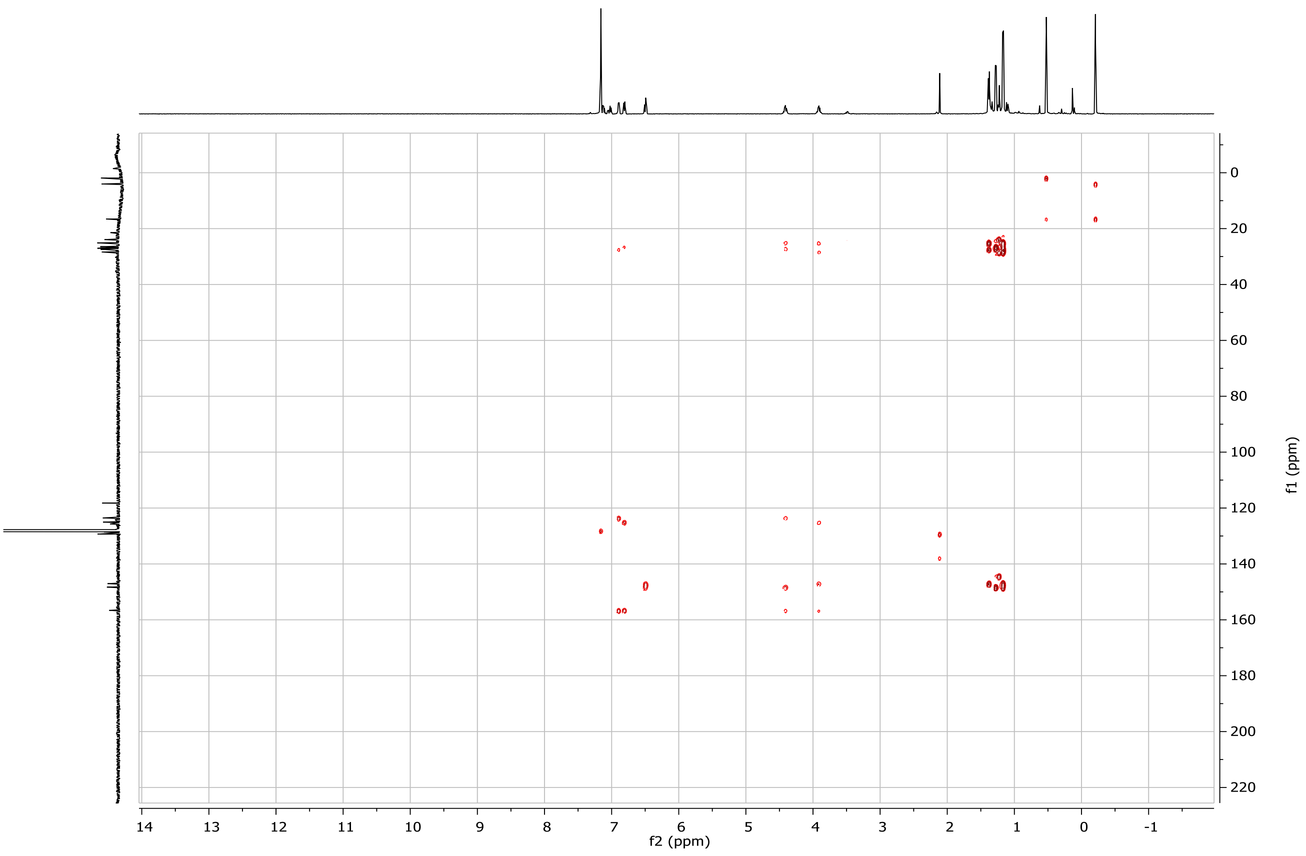

Synthesis of [{SiN^{Dipp}}Mg(CO)Na]$_2$ (**10**)

[{SiN^{Dipp}}$_2$MgNa]$_2$ (**9**) (10.8 mg, 0.02 mmol) was dissolved in 0.4 mL of C_6D_6 inside a J Youngs NMR tube. The solution was then degassed by three cycles of freeze-pump-thaw before the tube was charged with 2 atm of ^{13}CO. A diagnostic signal at 50 ppm in the ^{13}C{^{1}H} NMR spectrum was observed forming within an hour after the gas was added to the reaction mixture. Quantitative conversion to compound **10** (determined by ^{1}H NMR) was observed after the reaction mixture was left at room temperature for 3 days. All volatiles were then removed under vacuum, giving **10** as colorless solid. Crystals suitable for X-ray diffraction analysis were obtained by slow evaporation of a hexane solution at room temperature. Yield 9.0 mg, 79%. No meaningful result was obtained for elemental analysis, even after several attempts. ^{1}H NMR (500 MHz, 298K, Benzene-d$_6$) δ 6.88 (d, J = 7.4 Hz, 4H, *m*-C$_6$*H$_3$*), 6.65 (t, J = 7.4 Hz, 2H, *p*-C$_6$*H$_3$*), 4.02 (sept, J = 6.8 Hz, 4H, C*H*Me$_2$), 1.31 (d, J = 6.8 Hz, 12H, CH*Me$_2$*), 1.11 (s, 4H, SiC*H$_2$*), 0.97 (d, J = 6.8 Hz, 12H, CH*Me$_2$*), 0.19 (s, 12H, Si*Me$_2$*). ^{13}C NMR (126 MHz, 298K, Benzene-d$_6$) δ 153.8 (*i*-C$_6$H$_3$), 147.5 (*p*-*C*$_6$H$_3$), 121.6 (*m*-*C*$_6$H3), 118.3 (*p*-*C*$_6$H$_3$), 50.2 (*C*$_2$O$_2$), 27.3 (*C*HMe$_2$), 25.1 (CH*Me$_2$*), 23.7 (CH*Me$_2$*), 11.9 (Si*C*H$_2$), 1.1 (Si*Me$_2$*). Peaks observed at 160-180 ppm, no correlation with proton observed in ^{1}H-^{13}C HSQC, HMBC, plausibly ^{13}C labelled impurities.

Figure S9. ^{1}H NMR Spectrum of **10** (500 MHz, C_6D_6); #toluene.

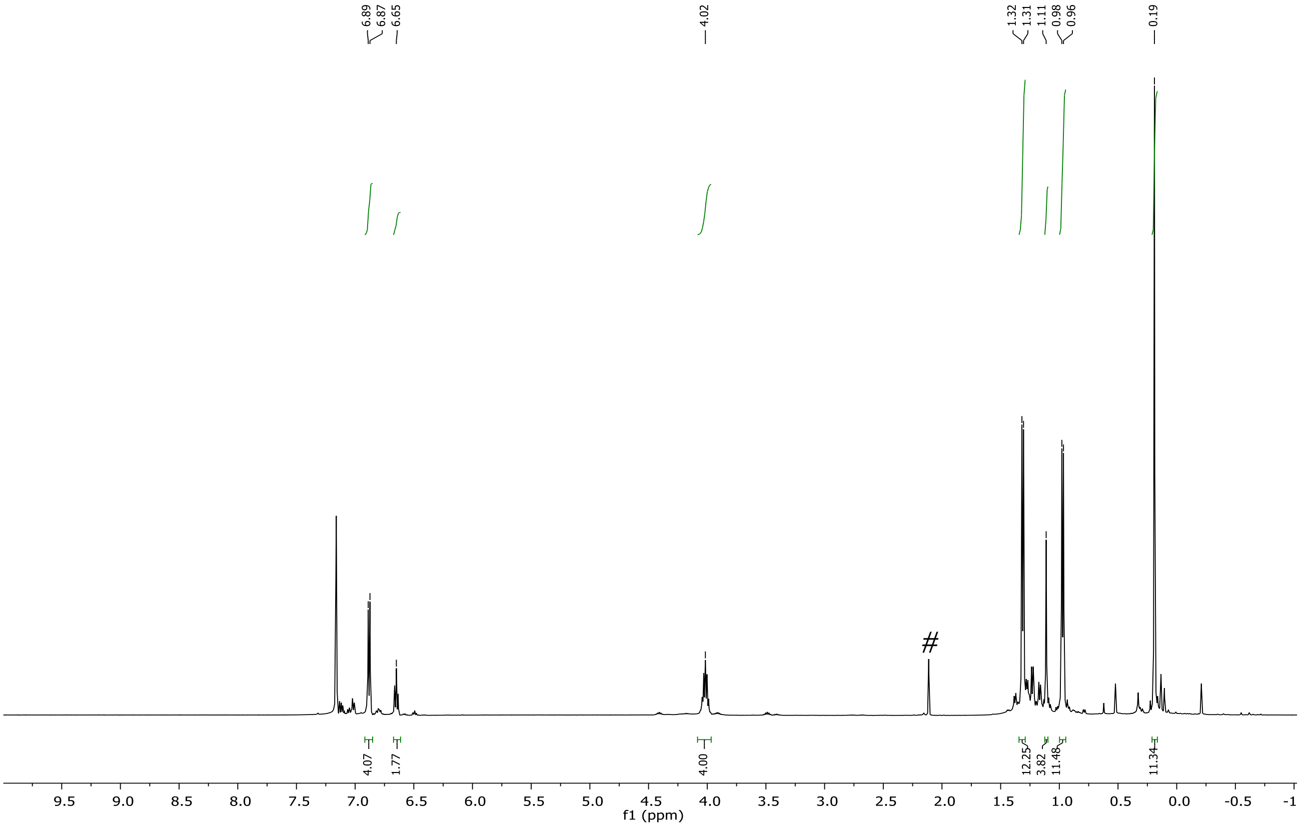

Figure S10. $^{13}C\{^1H\}$ NMR Spectrum of **10** (126 MHz, C_6D_6); #residual ^{13}CO

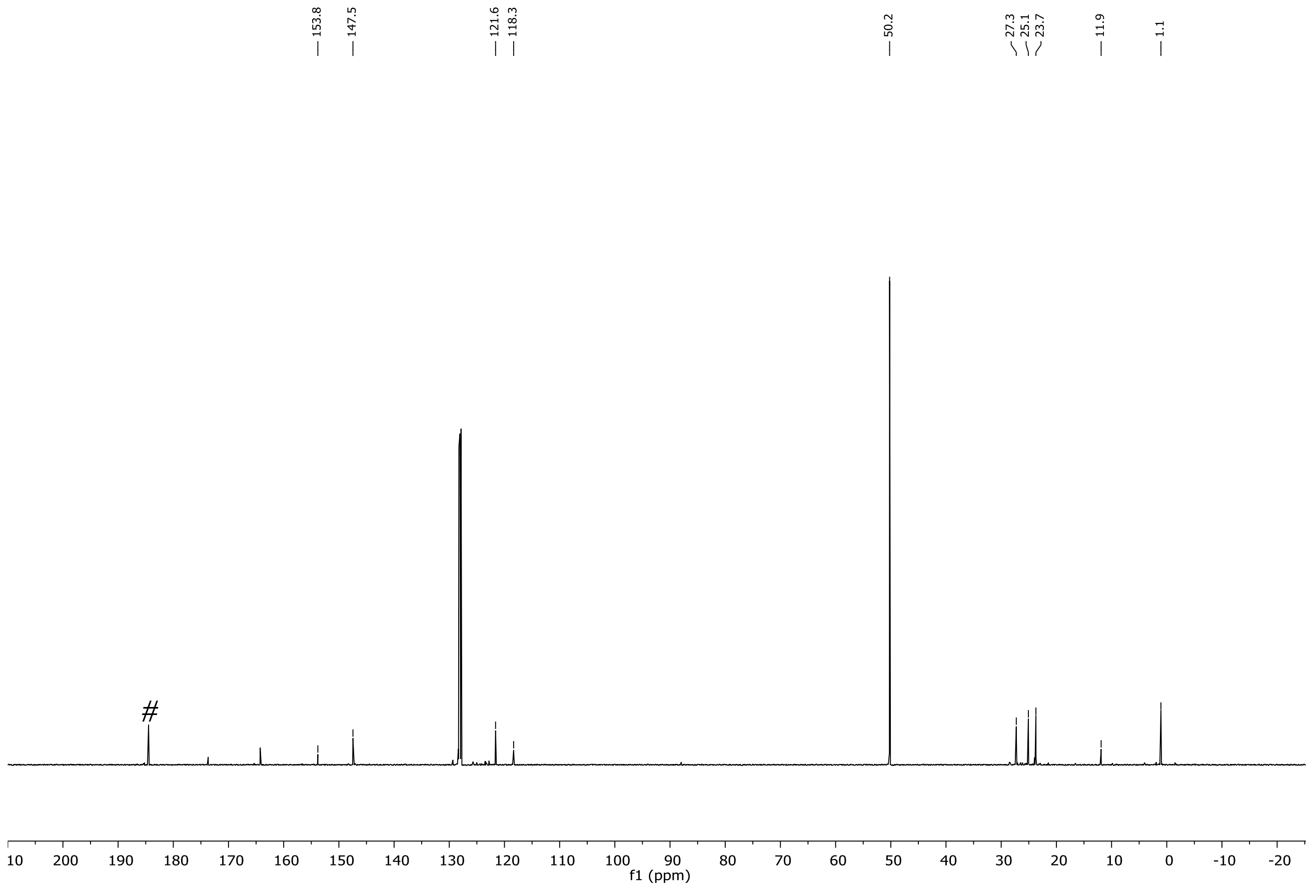

Figure S11. ^1H-^1H COSY NMR Spectrum of **10**.

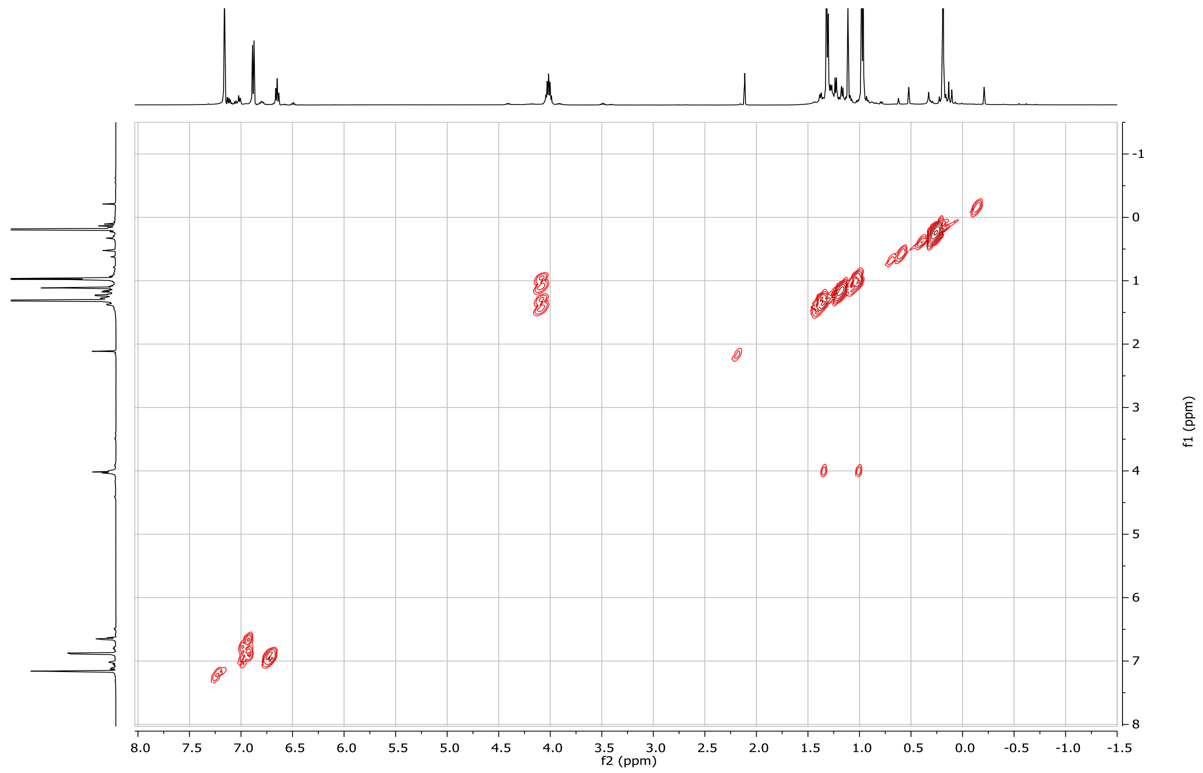

Figure S12. ^{1}H-^{13}C HSQC NMR Spectrum of **10**.

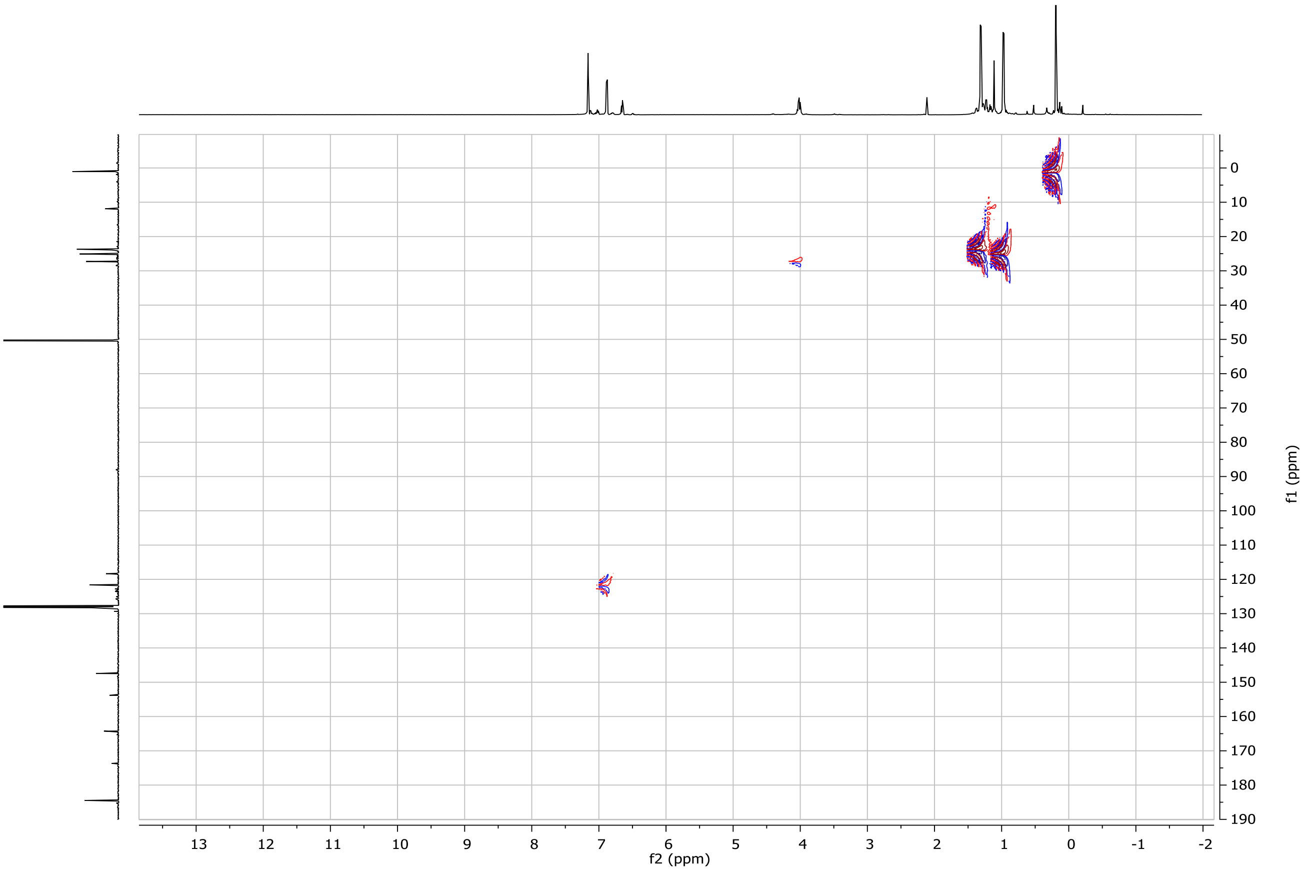

Figure S13. ^{1}H-^{13}C HMBC NMR Spectrum of **10**.

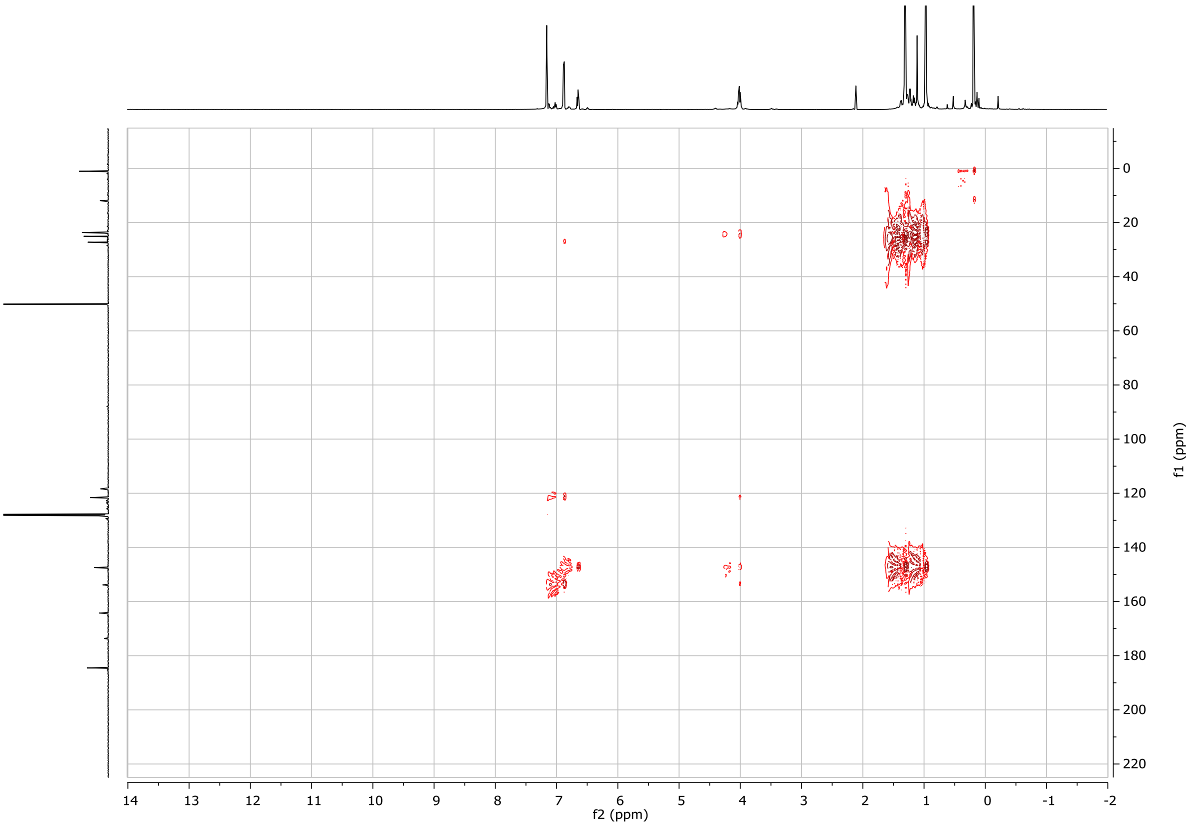

Single Crystal X-ray Diffraction Analysis

Data were collected for compounds **8**·benzene, **8**·toluene and **10** on a SuperNova, Dual Cu at zero, EosS2 diffractometer (Cu*K*α; λ = 1.54184 Å), whereas data for compound **9** were collected on a New Xcalibur, EosS2 diffractometer (MoKα; λ = 0.71073 Å). The crystals were all maintained at 150(2) K during data collection. Using Olex2,[2] the structures were solved with ShelXT[3] and refined with the ShelXL[4] using Least Squares minimization.

H31 (attached to C31) was located and refined subject to being a distance of 0.93Å from the parent atom in the structure of **8**·benzene. Additionally, atoms Si1, Si2 and C13-C18 were treated for 65:35 disorder with the inclusion of distance and some ADP restraints, to assist convergence.

H31 (attached to C31) was located and refined subject to being a distance of 0.93Å from the parent atom in the structure of **8**·toluene.

There are 2 molecules of the magnesium complex, and 4 molecules of toluene in the asymmetric unit of compound **9**. Disorder was confined to a pair of solvent molecules, each split into 2 components in a 70:30 ratio. Distance and ADP restraints were employed in the latter areas, to assist convergence.

In the structure of compound **10**, the asymmetric unit comprises one molecule of the complex and a region of solvent. The latter was identifiable as a partial-occupancy hexane moiety but, as it was also disordered, it was addressed using the solvent mask algorithm available in Olex-2. An allowance has been made, in the formula as presented, for the presence of 4 hexanes per unit cell. There was also 60:40 disorder present for Si3, Si4 and the carbons bonded to these 2 centres, as well as atoms C55, C56 and C57, which was readily modelled with the inclusion of distance and ADP restraints.

Table S1: Single crystal X-ray diffraction analysis of compounds **8**·benzene, **8**·toluene, **9** and **10**.

Compound	**8**·benzene	**8**·toluene	**9**	**10**
Empirical formula	$C_{36}H_{56}MgN_2Si_2$	$C_{37}H_{58}MgN_2Si_2$	$C_{148}H_{232}Mg_4N_8Na_4Si_8$	$C_{65}H_{107}Mg_2N_4Na_2O_2Si_4$
Formula weight	597.31	611.34	2537.32	1183.50
Crystal system	monoclinic	monoclinic	triclinic	monoclinic
Space group	$P2_1$	$P2_1$	*P*-1	*I*2/*a*
a/Å	9.2966(1)	9.2699(1)	19.7481(3)	23.4040(4)
b/Å	19.0191(1)	18.9358(2)	19.9460(3)	15.2673(3)
c/Å	10.5858(1)	10.6928(1)	20.2316(4)	40.5655(6)
α/°	90	90	77.123(2)	90
β/°	105.119(1)	102.036(1)	79.366(2)	99.641(2)
γ/°	90	90	89.5220(10)	90
U/Å^3	1806.92(3)	1835.68(3)	7630.8(2)	14290.0(4)
Z	2	2	2	8
ρ_{calc}g cm^{-3}	1.098	1.106	1.104	1.100
μ/mm^{-1}	1.236	1.227	0.147	1.377
F(000)	652.0	668.0	2760.0	5144.0
Crystal size/mm^3	0.301 × 0.187 × 0.161	0.222 × 0.164 × 0.121	0.548 × 0.373 × 0.27	0.232 × 0.115 × 0.085
2θ range for data collection/°	8.652 to 146.24	8.456 to 146.094	5.838 to 56.564	7.664 to 145.994
Index ranges	-11 ≤ h ≤ 11, -20 ≤ k ≤ 23, -12 ≤ l ≤ 13	-11 ≤ h ≤ 10, -23 ≤ k ≤ 21, -13 ≤ l ≤ 13	-26 ≤ h ≤ 26, -22 ≤ k ≤ 26, -26 ≤ l ≤ 26	-28 ≤ h ≤ 25, -18 ≤ k ≤ 18, -46 ≤ l ≤ 50
Reflections collected	20760	21713	84001	38609
Independent reflections, R_{int}	6080, 0.0354	6441, 0.0433	37247, 0.0439	14060, 0.0290
Data/restraints/parameters	6080/67/462	6441/2/396	37247/462/2279	14060/113/814
Goodness−of−fit on F^2	1.011	1.029	1.003	1.025
Final R1, wR_2[I>=2σ (I)]	0.0415, 0.1108	0.0468, 0.1222	0.0575, 0.1240	0.0380, 0.0998
Final R1, wR_2[all data]	0.0420, 0.1117	0.0477, 0.1237	0.1134, 0.1510	0.0456, 0.1053
Largest diff. peak/hole / e Å^{-3}	0.76/-0.29	0.52/-0.28	0.78/-0.40	0.25/-0.23
Flack parameter	0.040(15)	0.02(2)	-	-

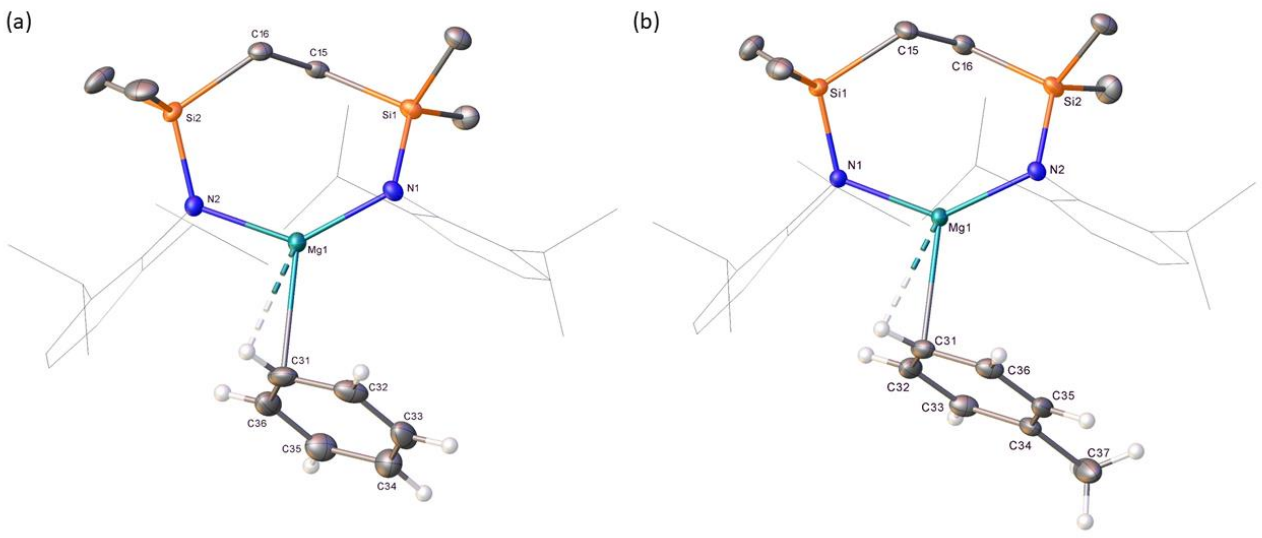

Figure S14: Displacement ellipsoid plot of (a) compound **8**·benzene and (b) compound **8**·toluene (30% probability ellipsoids)**.** Dipp substituents are shown as wireframe and hydrogen atoms, except those of the coordinated arenes, are omitted for clarity. Selected bond lengths (Å) and angles (°); **8**·benzene: Mg1-N1 1.947(2), Mg1-N2 1.948(2), Mg1-C31 2.501(3), N1-Mg1-N2 132.52(10), N1-Mg1-C31 120.17(11), N2-Mg1-C31 104.32(11); **8**·toluene: Mg1-N1 1.962(2), Mg1-N2 1.960(3), Mg1-C31 2.469(3), N2-Mg1-N1 133.92(11), N1-Mg1-C31 104.49(12), N2-Mg1-C31 118.82(12).

Computational Details

DFT calculations were run with Gaussian 16 (A.03).[5] The Na, Mg, Al, Si and K centres were described with the Stuttgart RECPs and associated basis sets,[6] and the 6-31G** basis set was used for all other atoms (BS1).[7] A polarization function was also added to Al (ζ_d = 0.190), Si (ζ_d = 0.284) and K (ζ_d = 1.000). Initial BP86 optimizations were performed using the 'grid = ultrafine' option,[8] with all stationary points being fully characterized via analytical frequency calculations as minima or transition states (all positive eigenvalues or one imaginary eigenvalue respectively). All energies were recomputed with a larger basis set featuring 6-311++G** basis sets on all atoms (BS2). Corrections for the effect of benzene (ε = 2.2706) solvent were run using the polarizable continuum model and BS1.[9] Single-point dispersion corrections to the BP86 results employed Grimme's D3 parameter set with Becke-Johnson damping as implemented in Gaussian.[10] Wiberg bond indices (WBI) were calculated using NBO v3.1 employed within Gaussian 16 (A.03).[11]

The Quantum Theory of Atoms in Molecules (QTAIM, AIMALL program[12]) and Natural Bonding Orbital (NBO7[13]) analyses were performed on the BP86-optmised geometries **9^{Na}, 10** (DFT-optimized) and **9^{K}**. The QTAIM topological analyses used wavefunction files obtained with Gaussian 16 (C.01) at the BP86/ 6-311++G** level, whilst NBO analyses were carried out with NBO 7 within Gaussian (C.01) at the same methodology level as the QTAIM calculations. Contour plots were generated in the AIMStudio package, using critical point (CP) visualisation threshold values of 0.02 e·$Å^{-3}$ (solid line BCP = strong) and 0.005 e·$Å^{-3}$ (dashed line BCP = weak). The NBO energies of donor-acceptor interactions ("$\Delta E^{(2)}$") between the various molecular fragments of the structures were estimated with second-order perturbation theory analysis of the Fock matrix in the NBO basis, as calculated by NBO7, with selected donor-acceptor NBO interactions provided.

Breakdown of Energy Contributions

The following tables detail the evolution of the relative energies as the successive corrections to the initial SCF energy are included. Terms used are:

$\mathbf{\Delta E_{BSI}}$	SCF energy computed with the BP86 functional with BS1
$\mathbf{\Delta H_{BSI}}$	Enthalpy at 0 K with BS1
$\mathbf{\Delta G_{BSI}}$	Free energy at 298.15 K and 1 atm with BS1
$\mathbf{\Delta G_{BSI/\mathit{bnz}}}$	Free energy corrected for benzene solvent with BS1
$\mathbf{\Delta G_{BSI/\mathit{bnz}+D3BJ}}$	Free energy corrected for benzene and dispersion effects with BS1
$\mathbf{\Delta E_{BS2}}$	SCF energy computed with the BP86 functional with BS2
$\mathbf{\Delta G_{bnz}}$	Free energy corrected for basis set (BS2), dispersion effects and benzene solvent

In each case the final data used in the main article are highlighted in bold.

Table S2. Relative stability energies of $\mathbf{9^M}$ dimers. Data in bold are those used in the main text. Energies are quoted relative to monomer **8** and the equivalent group 1 metal (**M**), at 0.0 kcal mol^{-1}.

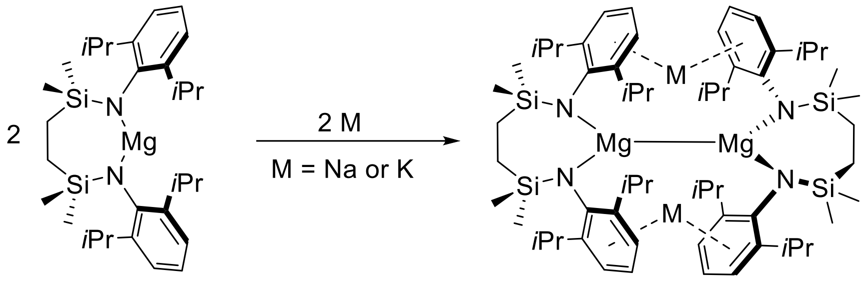

8 $\mathbf{9^M}$

9^M	M	ΔE_{BSI}	ΔH_{BSI}	ΔG_{BSI}	$\Delta G_{BSI/bnz}$	$\Delta G_{BSI/bnz+D3BJ}$	ΔE_{BS2}	ΔG_{bnz}
**9^{Na}**	**Na**	−75.0	−72.5	−32.7	−27.3	−82.4	−64.9	**−72.3**
**9^{K}**	**K**	−60.7	−59.0	−22.9	−17.3	−58.2	−66.2	**−63.7**

As seen in Table S2 above, analysis of the computed corrections for the reductive formation of a Mg—Mg bond in $\mathbf{9^M}$, sees a large stabilisation of $\mathbf{9^M}$ when the dispersion (D3BJ) component is included, compensating for the destabilisation of using free energies.

Table S3. BP86/BS1 Computed Wiberg Bond Indices (WBIs)

	Mg1-Mg2	M1-M2	Mg1-M1	Mg1-M2
**9^{Na}**	0.6562	0.0024	0.0517	0.0518
**A**	0.4957	0.0015	0.0305	0.0524
**B**	0.0047	0.0010	0.0037	0.0109
**C**	0.0011	0.0010	0.0104	0.0038
**10**	0.0000	0.0002	0.0002	0.0005
**9^{K}**	0.4209	0.0338	0.1455	0.1455

Table S4. Relative energies for computed structures. Data in bold are those used in the main text. Energies are quoted relative to complex **9** at 0.0 kcal mol^{-1}.

	ΔE_{BSI}	ΔH_{BSI}	ΔG_{BSI}	$\Delta G_{BSI/bnz}$	$\Delta G_{BSI/bnz+D3BJ}$	ΔE_{BS2}	ΔG_{bnz}
**9**	0.0	0.0	0.0	0.0	0.0	0.0	**0.0**
**9 + CO**	−1.1	−1.1	2.6	5.0	3.5	0.3	**4.9**
**TS(9-A)**	28.2	27.9	36.6	37.3	26.5	29.9	**29.2**
**A**	6.9	8.1	16.7	19.6	11.9	10.0	**15.0**
**TS(A-B)**	6.5	7.4	17.2	19.1	14.5	9.2	**17.2**
**B**	−23.9	−23.1	−15.6	−10.8	−14.0	−19.9	**−10.0**
**B + CO**	−22.5	−20.1	−0.7	5.0	−8.7	−14.1	**−0.3**
**C**	−27.7	−25.4	−7.4	−2.2	−13.0	−19.8	**−5.0**
**TS(C-10)**	−26.1	−24.0	−6.5	−1.8	−10.7	−18.1	**−2.8**
**10**	−90.9	−87.6	−75.0	−71.6	−70.8	−85.6	**−65.6**
**TS(B-D)**	−17.8	−15.9	2.0	5.7	−0.9	−10.5	**6.4**
**D**	−25.2	−23.2	−5.4	−1.2	−9.4	−18.2	**−2.4**
**TS(D-10)**	−14.7	−13.4	4.0	7.8	1.0	−7.8	**7.9**
**E**	−58.5	−55.7	−40.4	−36.5	−39.8	−49.0	**−30.3**
**TS(E-10)**	−56.3	−54.1	−35.3	−30.2	−37.0	−45.7	**−26.4**
**A + CO**	4.2	5.8	20.5	23.6	14.6	9.5	**19.9**
**TS(A-10)**	11.7	14.0	32.9	34.1	27.2	18.3	**33.8**

Discounted Mechanistic Steps

Beyond the mechanistic pathway described in the main text, multiple alterative steps were thoroughly evaluated, and are included in Table S4 above and shown below. From our investigations we found the free energy preference for coordination of CO *via* C at the Mg centre in the neutral monomer **8,** was 6.4 kcal mol^{-1}.

*Concerted Symmetric C-C coupling via **D***

A second route to form **10** was computationally isolated, involving intermediate **D**, at −2.4 kcal mol^{-1}, which has both CO molecules symmetrically coordinated within the [Na_2Mg_2] unit, with bond distances of Mg—O = 2.19 Å, Mg···C = 2.30 Å, C···C = 2.96 Å and C=O = 1.22 Å. Insertion of the second CO to **B** to afford **D** has a barrier of 6.7 kcal mol^{-1}. From this adduct, C-C coupling proceeds with an overall barrier, **TS(D-10)**, of 10.3 kcal mol^{-1}, higher than **TS(C-10)** reported in the main text by 10.3 kcal mol-^{1}, and with a larger coupling barrier by 8.1 kcal mol^{-1}.

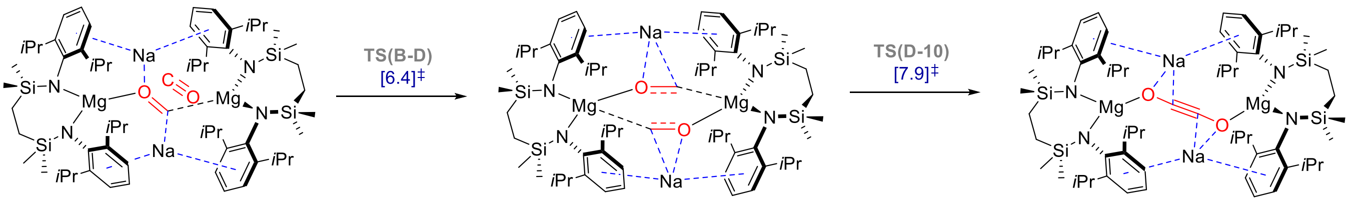

B + CO
-0.3

D
-2.4

10
-65.6

*Rotation of bridging OCCO ligand within dimer (**E**)*

An alternative O=C-C=O dimer Mg complex minimum was computationally isolated, **E**, with single bond character between the two carbon atoms (C—C = 1.55 Å). In this species the "OCCO" is bent with distinct angles of 111 ° at the carbons, and both Mg centres coordinating to one carbon each (Mg—C = 2.25 Å), whilst each terminal oxygen interacts with a Na cation (Na···O = 2.20 Å). **E** is 35.3 kcal mol^{-1} higher in free energy than the linear dimer species, **10**, and a free energy barrier of 3.9 kcal mol^{-1} will convert **E** to **10** as the OCCO fragment rotates within the [Na_2Mg_2] unit.

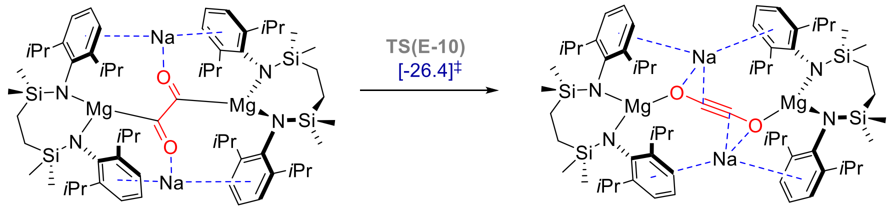

E -30.3 | **10** -65.6

*Outer-sphere 2nd CO Addition to **A***

Finally, outer-sphere C-C coupling of the second CO to **A** was also explored. Addition of the second uncoordinated CO to **A** raised the free energy of the system by 4.9 kcal mol^{-1}, the same as that seen for the first CO molecule to **9** (to form "**9 + CO**"). The C-C coupling step, **TS(A-10)**, was located at 33.8 kcal mol^{-1}, with a barrier of 13.9 kcal mol^{-1}, ~ 11 kcal mol^{-1} larger than the coupling reported in the manuscript for **TS(C-10)**, and significantly higher in free energy as well.

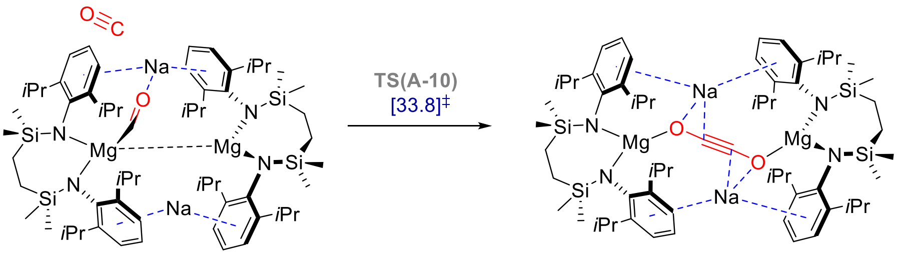

A + CO 19.9 | **10** -65.6

QTAIM Contour Plots & Tabulated Data

9^{Na}

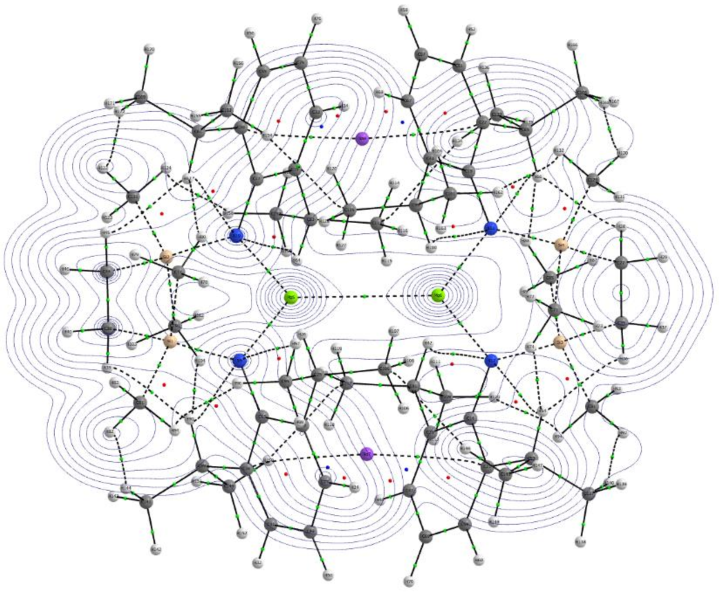

Figure S15. Contour plot of $\mathbf{9^{Na}}$

Table S5. Selected BCP data for $\mathbf{9^{Na}}$ (BCPs under the threshold of 0.005 are denoted with an asterisk).

BCP	ρ(r)	$\nabla^2\rho(r)$	ε	G(r)	V(r)	H(r)
Mg5 - Mg6	0.019377	-0.0136	0.097389	0.000217	-0.00384	-0.00362
Na7 - C16	0.010886	0.047496	4.784761	0.009973	-0.00807	0.001901
Na7 - C21	0.01088	0.047462	4.793043	0.009966	-0.00807	0.0019
Na8 - C30	0.010877	0.047456	4.761401	0.009964	-0.00807	0.0019
Na8 - C22	0.010883	0.047486	4.767484	0.009971	-0.00807	0.001901
Mg5 - Na8*	0.003418	0.005366	1.198502	0.001173	-0.001	0.000169
Mg6 - Na7*	0.003424	0.005373	1.189009	0.001175	-0.00101	0.000169

Table S6. Selected QTAIM atomic data for $\mathbf{9^{Na}}$.

Atom	q(A)	L(r)	N(r)	Vol(r)	Loc(r)
Mg5	0.973856	0.000279	11.026144	153.216513	93.211588
Mg6	0.973452	0.000118	11.026548	153.34964	93.209562
Na7	0.865465	0.000032	10.134535	75.166084	98.080039
Na8	0.865492	0.000015	10.134508	75.184244	98.080283

10

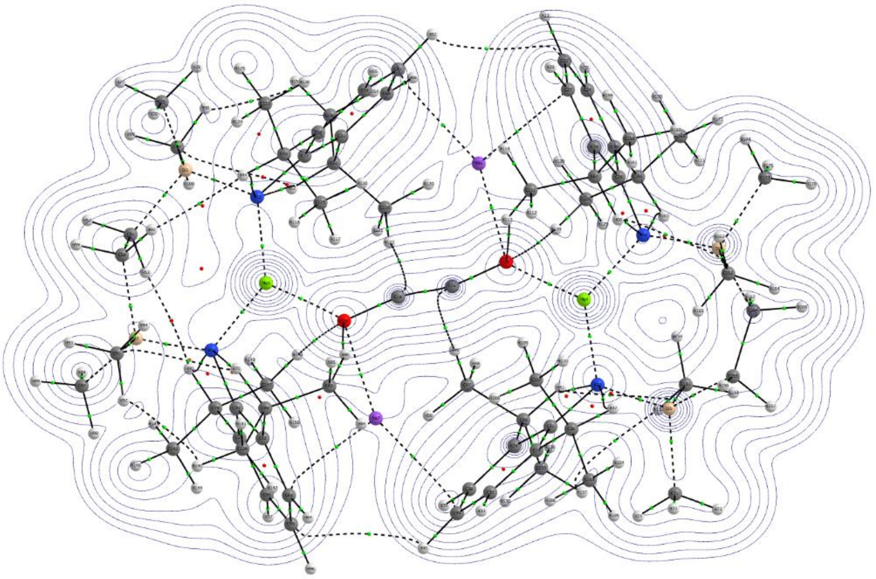

Figure S16. Contour plot of the DFT-optimized structure of **10**.

Table S7. BCP data for the DFT-optimized structure of **10**.

BCP	ρ(r)	$\nabla^2\rho(r)$	ε	G(r)	V(r)	H(r)
Mg3 - O10	0.050604	0.400409	0.072636	0.087073	-0.07404	0.013029
Na7 - O10	0.023174	0.140863	0.032748	0.029788	-0.02436	0.005427
Na7 - C63	0.007486	0.033132	2.484156	0.006741	-0.0052	0.001542
Na7 - C45	0.010088	0.042451	1.050395	0.00871	-0.00681	0.001903
H46 - C95	0.005224	0.014889	1.616252	0.002994	-0.00227	0.000729
Mg4 - O9	0.05182	0.414252	0.073537	0.090189	-0.07682	0.013374
Na6 - O9	0.023849	0.145946	0.047541	0.030866	-0.02524	0.005621
Na6 - C81	0.010411	0.044047	0.713685	0.009098	-0.00718	0.001914
Na6 - C27	0.007292	0.032428	2.754783	0.006597	-0.00509	0.00151
C22 - H82	0.005171	0.0147	1.609021	0.00295	-0.00223	0.000725

Table S8: Selected QTAIM atomic data for DFT-optimized **10**.

Atom	q(A)	L(r)	N(r)	Vol(r)	Loc(r)
Mg3	1.692665	0.000165	10.307335	49.247704	96.362809
Mg4	1.686729	0.000095	10.313271	49.442639	96.286965
Na6	0.901595	0.000165	10.098405	73.844495	98.422998
Na7	0.901716	0.0002	10.098284	73.780229	98.423702
O9	-1.282078	0.000044	9.282078	126.361535	89.240966
O10	-1.278866	0.000027	9.278866	122.053742	89.074683

9^{K}

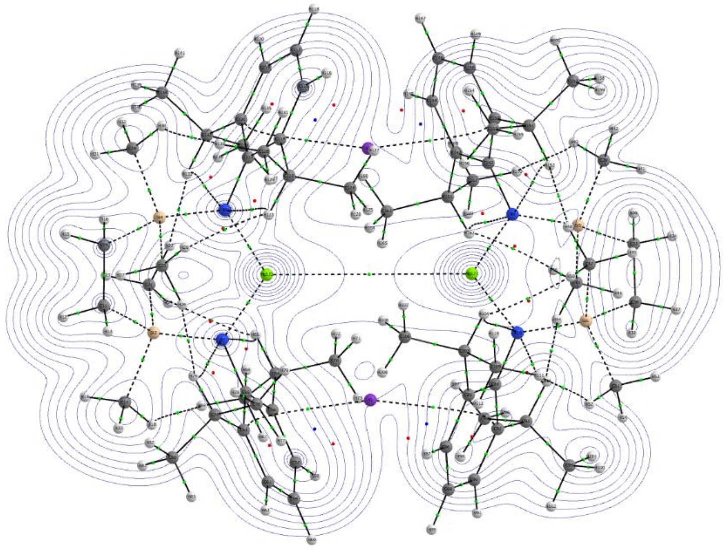

Figure S17. Contour plot of **9^{K}**.

Table S9. BCP data for **9^{K}**. (BCPs under the threshold of 0.005 are denoted with an asterisk).

BCP	ρ(r)	∇^2ρ(r)	ε	G(r)	V(r)	H(r)
Mg171 - Mg172	0.006661	-0.001	0.33906	0.000041	-0.00033	-0.000291
K2 - C121	0.00922	0.032398	18.15254	0.006706	-0.00531	0.001394
K2 - C150	0.009224	0.032415	16.5404	0.006709	-0.00532	0.001394
K1 - C63	0.009221	0.032405	16.53761	0.006707	-0.00531	0.001394
K1 - C92	0.009216	0.032384	18.47214	0.006703	-0.00531	0.001393
K2 - Mg172*	0.004782	0.002763	36.4315	0.000757	-0.00082	-0.000067
K2 - Mg171*	0.004782	0.002767	33.04265	0.000758	-0.00083	-0.000067
K1 - Mg172*	0.004781	0.002767	32.73572	0.000758	-0.00083	-0.000067
K1 - Mg171*	0.004781	0.00276	38.32154	0.000757	-0.00082	-0.000067

Table S10. Selected QTAIM atomic data for **9^{K}**.

Atom	L(r)	N(r)	Vol(r)	Loc(r)	q(r)
Mg171	0.988794	0.000108	11.011206	196.803669	93.02912
Mg172	0.988492	-0.000013	11.011508	196.874807	93.027996
K1	0.797765	0.000063	18.202235	165.479457	98.213442
K2	0.79763	-0.000022	18.20237	165.460003	98.212719

NBO Data

Figure S18. Natural Localized Molecular Orbital of Mg-Mg bond in **9^{Na}**.

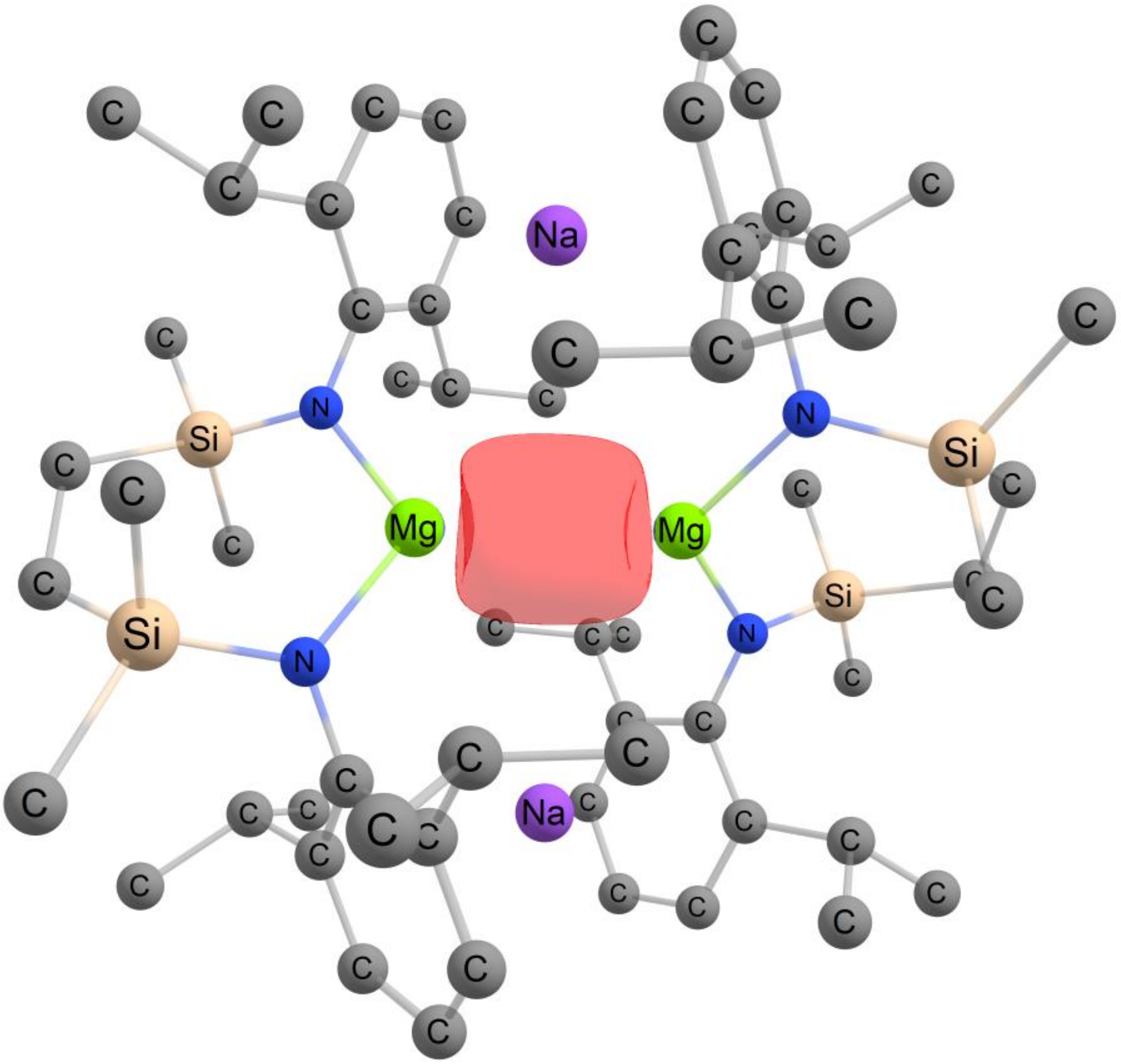

Table S11. Atomic contributions to the $\sigma_{Mg\text{-}Mg}$ bond NLMO.

Atom	Contribution to NLMO in % (contributing s, p and d character)
Mg (left)	44.06 (95.1% s character, 4.63% p)
Mg (right)	43.15 (94.8% s character, 4.96% p)

Second Order Perturbation Theory Analysis of Donor Acceptor NBO interactions between molecular fragments

Table S12. Selected donor acceptor interaction energies, $\Delta E^{(2)}$, for $\mathbf{9}^{\mathbf{Na}}$

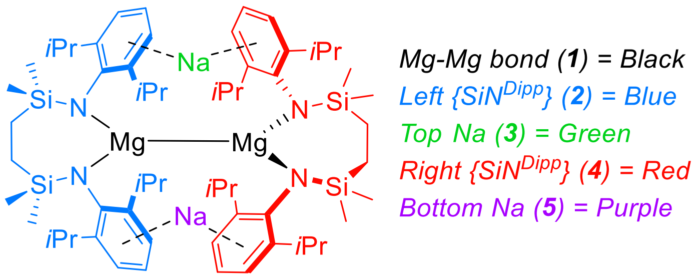

Donor NBO (Unit)	Acceptor NBO (Unit)	$\Delta E^{(2)}$ (kcal/mol)
$\Omega_{Mg5\text{-}Mg6}$ (1)	n_{Na7}* (3)	25.4
$\Omega_{Mg5\text{-}Mg6}$ (1)	r_{Na7}* (3)	4.3
$\Omega_{C16\text{-}C31}$ (2)	n_{Na7}* (3)	2.3
$\Omega_{C13\text{-}C18}$ (2)	n_{Na7}* (3)	2.3
$\Omega_{C13\text{-}C16}$ (2)	n_{Na7}* (3)	2.2
$\Omega_{C18\text{-}C23}$ (2)	n_{Na7}* (3)	2.1
$\Omega_{C49\text{-}H50}$ (2)	n_{Na7}* (3)	1.9
$\Omega_{C31\text{-}H32}$ (2)	n_{Na7}* (3)	1.7
$\Omega_{C16\text{-}C89}$ (2)	n_{Na7}* (3)	1.7
$\Omega_{C23\text{-}H24}$ (2)	n_{Na7}* (3)	1.6
$\Omega_{C18\text{-}C25}$ (2)	n_{Na7}* (3)	1.5
$\Omega_{C31\text{-}C49}$ (2)	n_{Na7}* (3)	1.4
$\Omega_{C23\text{-}C49}$ (2)	n_{Na7}* (3)	1.4
$\Omega_{C21\text{-}C59}$ (4)	n_{Na7}* (3)	2.3
$\Omega_{C14\text{-}C20}$ (4)	n_{Na7}* (3)	2.3
$\Omega_{C14\text{-}C21}$ (4)	n_{Na7}* (3)	2.2
$\Omega_{C20\text{-}C47}$ (4)	n_{Na7}* (3)	2.1
$\Omega_{C69\text{-}H70}$ (4)	n_{Na7}* (3)	1.9
$\Omega_{C59\text{-}H60}$ (4)	n_{Na7}* (3)	1.7
$\Omega_{C21\text{-}C53}$ (4)	n_{Na7}* (3)	1.7
$\Omega_{C47\text{-}H48}$ (4)	n_{Na7}* (3)	1.6
$\Omega_{C20\text{-}C41}$ (4)	n_{Na7}* (3)	1.5
$\Omega_{C59\text{-}C69}$ (4)	n_{Na7}* (3)	1.4
$\Omega_{C47\text{-}C69}$ (4)	n_{Na7}* (3)	1.4

Donor NBO (Unit)	Acceptor NBO (Unit)	$\Delta E^{(2)}$ (kcal/mol)
$\Omega_{Mg5\text{-}Mg6}$ (1)	n_{Na7}* (5)	25.4
$\Omega_{Mg5\text{-}Mg6}$ (1)	r_{Na7}* (5)	4.3
$\Omega_{C30\text{-}C55}$ (2)	n_{Na7}* (5)	2.3
$\Omega_{C15\text{-}C17}$ (2)	n_{Na7}* (5)	2.3
$\Omega_{C17\text{-}C30}$ (2)	n_{Na7}* (5)	2.2
$\Omega_{C15\text{-}C33}$ (2)	n_{Na7}* (5)	2.1
$\Omega_{C75\text{-}H76}$ (2)	n_{Na7}* (5)	1.9
$\Omega_{C55\text{-}H56}$ (2)	n_{Na7}* (5)	1.7
$\Omega_{C30\text{-}C61}$ (2)	n_{Na7}* (5)	1.7
$\Omega_{C33\text{-}H34}$ (2)	n_{Na7}* (5)	1.6
$\Omega_{C15\text{-}C63}$ (2)	n_{Na7}* (5)	1.5
$\Omega_{C55\text{-}C75}$ (2)	n_{Na7}* (5)	1.4
$\Omega_{C33\text{-}C75}$ (2)	n_{Na7}* (5)	1.4
$\Omega_{C22\text{-}C51}$ (4)	n_{Na7}* (5)	2.3
$\Omega_{C19\text{-}C43}$ (4)	n_{Na7}* (5)	2.3
$\Omega_{C19\text{-}C22}$ (4)	n_{Na7}* (5)	2.2
$\Omega_{C43\text{-}C67}$ (4)	n_{Na7}* (5)	2.1
$\Omega_{C57\text{-}H58}$ (4)	n_{Na7}* (5)	1.9
$\Omega_{C51\text{-}H52}$ (4)	n_{Na7}* (5)	1.7
$\Omega_{C22\text{-}C65}$ (4)	n_{Na7}* (5)	1.7
$\Omega_{C67\text{-}H68}$ (4)	n_{Na7}* (5)	1.6
$\Omega_{C43\text{-}C99}$ (4)	n_{Na7}* (5)	1.5
$\Omega_{C51\text{-}C57}$ (4)	n_{Na7}* (5)	1.4
$\Omega_{C57\text{-}C67}$ (4)	n_{Na7}* (5)	1.4

Table S13. Selected donor acceptor interaction energies, $\Delta E^{(2)}$, for DFT-optimized **10.**

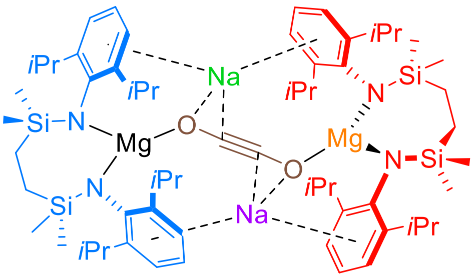

Donor NBO (Unit)	Acceptor NBO (Unit)	$\Delta E^{(2)}$ (kcal/mol)
LP_{O10} (3)	n_{Mg3}* (1)	8.4
$\Omega_{C15-C18}$ (3)	n_{Mg3}* (1)	2.4
$\Omega_{O10-C18}$ (3)	n_{Mg3}* (1)	1.8
$\Omega_{C15-C18}$ (3)	n_{Na7}* (5)	2.6
LP_{O10} (3)	n_{Na7}* (5)	2.3
$\Omega_{O10-C18}$ (3)	n_{Na7}* (5)	1.5
$\Omega_{C21-C59}$ (4)	n_{Na7}* (5)	1.8
$\Omega_{C14-C20}$ (4)	n_{Na7}* (5)	1.8
$\Omega_{C14-C21}$ (4)	n_{Na7}* (5)	1.7
$\Omega_{C20-C47}$ (4)	n_{Na7}* (5)	1.5
$\Omega_{C69-H70}$ (4)	n_{Na7}* (5)	1.4
$\Omega_{C59-H60}$ (4)	n_{Na7}* (5)	1.3
$\Omega_{C21-C53}$ (4)	n_{Na7}* (5)	1.3
$\Omega_{C47-H48}$ (4)	n_{Na7}* (5)	1.3
$\Omega_{C20-C41}$ (4)	n_{Na7}* (5)	1.3
$\Omega_{C59-C69}$ (4)	n_{Na7}* (5)	1.2
$\Omega_{C21-C59}$ (4)	n_{Na7}* (5)	1.1
$\Omega_{C21-C59}$ (6)	n_{Na7}* (5)	1.7
$\Omega_{C14-C20}$ (6)	n_{Na7}* (5)	1.6
$\Omega_{C14-C21}$ (6)	n_{Na7}* (5)	1.4
$\Omega_{C20-C47}$ (6)	n_{Na7}* (5)	1.1
$\Omega_{C69-H70}$ (6)	n_{Na7}* (5)	1.1
$\Omega_{C59-H60}$ (6)	n_{Na7}* (5)	1.1
$\Omega_{C21-C53}$ (6)	n_{Na7}* (5)	1.0
$\Omega_{C47-H48}$ (6)	n_{Na7}* (5)	1.0
$\Omega_{C20-C41}$ (6)	n_{Na7}* (5)	1.0
$\Omega_{C20-C41}$ (6)	n_{Na7}* (5)	1.0
$\Omega_{C20-C41}$ (6)	n_{Na7}* (5)	0.9

Donor NBO (Unit)	Acceptor NBO (Unit)	$\Delta E^{(2)}$ (kcal/mol)
LP_{O9} (3)	n_{Mg3}* (2)	8.7
$\Omega_{C15-C18}$ (3)	n_{Mg3}* (2)	2.3
Ω_{O9-C15} (3)	n_{Mg3}* (2)	1.6
$\Omega_{C15-C18}$ (3)	n_{Na7}* (7)	2.3
LP_{O9} (3)	n_{Na7}* (7)	2.0
Ω_{O9-C15} (3)	n_{Na7}* (7)	1.3
$\Omega_{C21-C59}$ (4)	n_{Na7}* (7)	1.7
$\Omega_{C14-C20}$ (4)	n_{Na7}* (7)	1.6
$\Omega_{C14-C21}$ (4)	n_{Na7}* (7)	1.4
$\Omega_{C20-C47}$ (4)	n_{Na7}* (7)	1.2
$\Omega_{C69-H70}$ (4)	n_{Na7}* (7)	1.2
$\Omega_{C59-H60}$ (4)	n_{Na7}* (7)	1.1
$\Omega_{C21-C53}$ (4)	n_{Na7}* (7)	1.1
$\Omega_{C47-H48}$ (4)	n_{Na7}* (7)	1.0
$\Omega_{C20-C41}$ (4)	n_{Na7}* (7)	1.0
$\Omega_{C59-C69}$ (4)	n_{Na7}* (7)	1.0
$\Omega_{C21-C59}$ (4)	n_{Na7}* (7)	1.0
$\Omega_{C21-C59}$ (6)	n_{Na7}* (7)	1.6
$\Omega_{C14-C20}$ (6)	n_{Na7}* (7)	1.6
$\Omega_{C14-C21}$ (6)	n_{Na7}* (7)	1.6
$\Omega_{C20-C47}$ (6)	n_{Na7}* (7)	1.6
$\Omega_{C69-H70}$ (6)	n_{Na7}* (7)	1.3
$\Omega_{C59-H60}$ (6)	n_{Na7}* (7)	1.3
$\Omega_{C21-C53}$ (6)	n_{Na7}* (7)	1.3
$\Omega_{C47-H48}$ (6)	n_{Na7}* (7)	1.2
$\Omega_{C20-C41}$ (6)	n_{Na7}* (7)	1.1
$\Omega_{C20-C41}$ (6)	n_{Na7}* (7)	1.1
$\Omega_{C20-C41}$ (6)	n_{Na7}* (7)	1.1

Table S14. Selected donor acceptor interaction energies, $\Delta E^{(2)}$, for $\mathbf{9^K}$

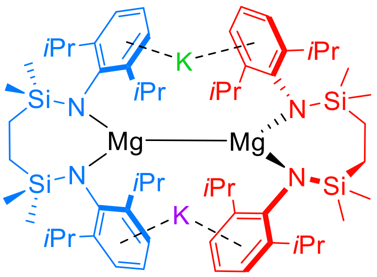

*Left Mg (**1**) = Black*
*Right Mg (**2**) = Orange*
*Left {SiN^{Dipp}} (**3**) = Blue*
*Top K (**4**) = Green*
*Right {SiN^{Dipp}} (**5**) = Red*
*Bottom K (**6**) = Purple*

Donor NBO (Unit)	Acceptor NBO (Unit)	ΔE$^{(2)}$ (kcal/mol)
n_{Mg172} (1)	r_{Mg171} (2)	2.2
n_{Mg172} (1)	n_{K1}* (4)	53.4
n_{Mg172} (1)	r_{K1}* (4)	13.0
$\Omega_{C55\text{-}C63}$ (3)	n_{K1}* (4)	1.3
$\Omega_{C59\text{-}H60}$ (3)	n_{K1}* (4)	1.3
$\Omega_{C55\text{-}C56}$ (3)	n_{K1}* (4)	1.2
$\Omega_{C61\text{-}C62}$ (3)	n_{K1}* (4)	1.2
$\Omega_{C61\text{-}H62}$ (3)	n_{K1}* (4)	1.2
$\Omega_{C63\text{-}C74}$ (3)	n_{K1}* (4)	1.1
$\Omega_{C56\text{-}C57}$ (3)	n_{K1}* (4)	1.1
$\Omega_{C57\text{-}H58}$ (3)	n_{K1}* (4)	1.0
$\Omega_{C56\text{-}C64}$ (3)	n_{K1}* (4)	1.0
$\Omega_{C76\text{-}H77}$ (3)	n_{K1}* (4)	0.9
$\Omega_{C70\text{-}H71}$ (3)	n_{K1}* (4)	0.8
$\Omega_{C90\text{-}C92}$ (5)	n_{K1}* (4)	1.3
$\Omega_{C84\text{-}C92}$ (5)	n_{K1}* (4)	1.3
$\Omega_{C88\text{-}H89}$ (5)	n_{K1}* (4)	1.3
$\Omega_{C84\text{-}C85}$ (5)	n_{K1}* (4)	1.2
$\Omega_{C85\text{-}C86}$ (5)	n_{K1}* (4)	1.2
$\Omega_{C90\text{-}H91}$ (5)	n_{K1}* (4)	1.1
$\Omega_{C92\text{-}C93}$ (5)	n_{K1}* (4)	1.1
$\Omega_{C86\text{-}H87}$ (5)	n_{K1}* (4)	1.0
$\Omega_{C85\text{-}C103}$ (5)	n_{K1}* (4)	1.0
$\Omega_{C95\text{-}H97}$ (5)	n_{K1}* (4)	0.9
$\Omega_{C95\text{-}H98}$ (5)	n_{K1}* (4)	0.9

Donor NBO (Unit)	Acceptor NBO (Unit)	ΔE$^{(2)}$ (kcal/mol)
n_{Mg172} (1)	n_{K2}* (6)	53.4
n_{Mg172} (1)	r_{K2}* (6)	12.2
$\Omega_{C119\text{-}C121}$ (3)	n_{K2}* (6)	1.3
$\Omega_{C113\text{-}C121}$ (3)	n_{K2}* (6)	1.3
$\Omega_{C117\text{-}H118}$ (3)	n_{K2}* (6)	1.3
$\Omega_{C113\text{-}C114}$ (3)	n_{K2}* (6)	1.2
$\Omega_{C114\text{-}C115}$ (3)	n_{K2}* (6)	1.2
$\Omega_{C119\text{-}H120}$ (3)	n_{K2}* (6)	1.1
$\Omega_{C121\text{-}C132}$ (3)	n_{K2}* (6)	1.1
$\Omega_{C115\text{-}H116}$ (3)	n_{K2}* (6)	1.0
$\Omega_{C114\text{-}C122}$ (3)	n_{K2}* (6)	1.0
$\Omega_{C134\text{-}H137}$ (3)	n_{K2}* (6)	0.9
$\Omega_{C134\text{-}H135}$ (3)	n_{K2}* (6)	0.9
$\Omega_{C142\text{-}C150}$ (5)	n_{K2}* (6)	1.3
$\Omega_{C146\text{-}H147}$ (5)	n_{K2}* (6)	1.3
$\Omega_{C142\text{-}C143}$ (5)	n_{K2}* (6)	1.2
$\Omega_{C148\text{-}C150}$ (5)	n_{K2}* (6)	1.2
$\Omega_{C150\text{-}H149}$ (5)	n_{K2}* (6)	1.2
$\Omega_{C150\text{-}H151}$ (5)	n_{K2}* (6)	1.1
$\Omega_{C143\text{-}C144}$ (5)	n_{K2}* (6)	1.1
$\Omega_{C144\text{-}H145}$ (5)	n_{K2}* (6)	1.0
$\Omega_{C143\text{-}C161}$ (5)	n_{K2}* (6)	1.0
$\Omega_{C153\text{-}C156}$ (5)	n_{K2}* (6)	0.9
$\Omega_{C153\text{-}C154}$ (5)	n_{K2}* (6)	0.9

Natural Atomic Charges

Table S15. Computed Natural atomic charges of $\mathbf{9^{Na}}$, **10** and $\mathbf{9^{K}}$.

s	Charge
$\mathbf{9^{Na}}$	
Mg5	1.00649
Mg6	1.02721
Na7	0.83922
Na8	0.83933
**10**	
Mg3	1.84492
Mg4	1.84495
Na6	0.90412
Na7	0.90119
O9	-1.01176
O10	-1.00573
$\mathbf{9^{K}}$	
K1	0.76553
K2	0.76542
Mg171	1.10732
Mg172	1.10635

References

1. Schwamm, R. J.; Coles, M. P.; Hill, M. S.; Mahon, M. F.; McMullin, C. L.; Rajabi, N. A.; Wilson, A. S. S. *Angew. Chem. Int. Ed.* **2020**, *59*, 3928.
2. Dolomanov, O. V.; Bourhis, L.J.; Gildea, R.J.; Howard, J. A. K.; Puschmann, H. *J. Appl. Cryst.* **2009**, *42*, 339-341.
3. Sheldrick, G. M. *Acta Cryst.* **2015**, *A71*, 3-8.
4. Sheldrick, G. M. *Acta Cryst.* **201**, *C71*, 3-8.
5. M. J. Frisch, G. W. Trucks, H. B. Schlegel, G. E. Scuseria, M. A. Robb, J. R. Cheeseman, G. Scalmani, V. Barone, G. A. Petersson, H. Nakatsuji, X. Li, M. Caricato, A. V. Marenich, J. Bloino, B. G. Janesko, R. Gomperts, B. Mennucci, H. P. Hratchian, J. V. Ortiz, A. F. Izmaylov, J. L. Sonnenberg, Williams, F. Ding, F. Lipparini, F. Egidi, J. Goings, B. Peng, A. Petrone, T. Henderson, D. Ranasinghe, V. G. Zakrzewski, J. Gao, N. Rega, G. Zheng, W. Liang, M. Hada, M. Ehara, K. Toyota, R. Fukuda, J. Hasegawa, M. Ishida, T. Nakajima, Y. Honda, O. Kitao, H. Nakai, T. Vreven, K. Throssell, J. A. Montgomery Jr., J. E. Peralta, F. Ogliaro, M. J. Bearpark, J. J. Heyd, E. N. Brothers, K. N. Kudin, V. N. Staroverov, T. A. Keith, R. Kobayashi, J. Normand, K. Raghavachari, A. P. Rendell, J. C. Burant, S. S. Iyengar, J. Tomasi, M. Cossi, J. M. Millam, M. Klene, C. Adamo, R. Cammi, J. W. Ochterski, R. L. Martin, K. Morokuma, O. Farkas, J. B. Foresman, D. J. Fox, Wallingford, CT, 2016.
6. D. Andrae, U. Häußermann, M. Dolg, H. Stoll, H. Preuß, Theor. Chim. Acta 1990, 77, 123-141.
7. a) P. C. Hariharan, J. A. Pople, Theor. Chim. Acta 1973, 28, 213-222; b) W. J. Hehre, R. Ditchfield, J. A. Pople, J. Chem. Phys. 1972, 56, 2257-2261.
8. a) A. D. Becke, Phys. Rev. A 1988, 38, 3098-3100; b) J. P. Perdew, Phys. Rev. B 1986, 33, 8822-8824.
9. J. Tomasi, B. Mennucci, R. Cammi, Chem. Rev. 2005, 105, 2999-3094.
10. S. Grimme, S. Ehrlich, L. Goerigk, J. Comp. Chem. 2011, 32, 1456-1465.
11. NBO 3.1, E. D. Glendening, A. E. Reed, J. E. Carpenter, and F. Weinhold, Theoretical Chemistry Institute, University of Wisconsin, Madison, USA, 2018.
12. AIMAll (Version 19.10.12), T. A. Keith, TK Gristmill Software, Overland Park KS, USA, 2019 (aim.tkgristmill.com).
13. NBO 7.0, E. D. Glendening, J. K. Badenhoop, A. E. Reed, J. E. Carpenter, J. A. Bohmann, C. M. Morales, P. Karafiloglou, C. R. Landis, and F. Weinhold, Theoretical Chemistry Institute, University of Wisconsin, Madison, USA, 2003.

Cartesian Coordinates and Computed Energies (in Hartrees) for Calculated Structures

Monomer to Gp1 Dimer Geometries

8

SCF (BP86) Energy = -1291.60521301
Enthalpy 0K = -1290.892021
Enthalpy 298K = -1290.845210
Free Energy 298K = -1290.973061
Lowest Frequency = 12.1095 cm^{-1}
Second Frequency = 15.8740 cm^{-1}
SCF (BP86-D3BJ) Energy = -1291.81348434
SCF (Benzene) Energy = -1291.61448887
SCF (BS2) Energy = -2062.42200384

Si -2.18872 -0.68198 2.04556
Si 2.18876 0.68155 2.04564
N -1.92507 -0.19623 0.36531
N 1.92506 0.19618 0.36530
C -0.39411 -0.67944 2.78276
H -0.51106 -1.04028 3.82079
H 0.19737 -1.49527 2.30677
C 0.39409 0.67931 2.78271
H 0.51099 1.04027 3.82070
H -0.19738 1.49514 2.30665
C -2.88985 -2.43728 2.29745
H -3.89590 -2.52922 1.85453
H -2.97884 -2.66727 3.37418
H -2.24882 -3.20705 1.83573
C -3.28930 0.52242 3.03082
H -2.79320 1.49232 3.20117
H -3.55666 0.09722 4.01375
H -4.22685 0.71769 2.48259
C 3.28886 -0.52332 3.03085
H 2.79262 -1.49323 3.20071
H 3.55592 -0.09846 4.01401
H 4.22659 -0.71849 2.48289
C 2.89051 2.43656 2.29783
H 3.89651 2.52823 1.85473
H 2.97979 2.66630 3.37459
H 2.24968 3.20666 1.83638
C -2.97824 0.14691 -0.53562
C -3.40535 1.51010 -0.66546
C -4.43437 1.83245 -1.56972
H -4.75908 2.87471 -1.66306
C -5.05078 0.85108 -2.35579
H -5.85297 1.12059 -3.05102
C -4.61784 -0.47501 -2.25089
H -5.08155 -1.24281 -2.88140
C -3.59356 -0.84887 -1.35942
C -2.69805 2.62343 0.10725
H -2.24712 2.15436 0.99921
C -3.63152 3.74979 0.59273
H -4.04747 4.33659 -0.24522
H -3.07811 4.45571 1.23664
H -4.47769 3.34711 1.17393
C -1.54219 3.20996 -0.73766
H -0.82581 2.42737 -1.05794
H -0.97517 3.97389 -0.17582
H -1.92734 3.67704 -1.66099
C -3.10675 -2.29752 -1.35155
H -2.43775 -2.40321 -0.48085
C -2.27129 -2.59381 -2.61958
H -2.88258 -2.48110 -3.53255
H -1.87278 -3.62403 -2.60282
H -1.41685 -1.89946 -2.71153
C -4.24989 -3.32368 -1.20575
H -4.86224 -3.12317 -0.31050
H -3.84361 -4.34706 -1.12049
H -4.92606 -3.31276 -2.07904
C 2.97824 -0.14680 -0.53567
C 3.40537 -1.50996 -0.66573
C 4.43447 -1.83213 -1.56998
H 4.75919 -2.87436 -1.66349
C 5.05091 -0.85060 -2.35583
H 5.85318 -1.11997 -3.05104
C 4.61794 0.47546 -2.25073
H 5.08168 1.24338 -2.88107
C 3.59359 0.84913 -1.35927
C 2.69806 -2.62346 0.10672
H 2.24685 -2.15453 0.99861
C 1.54247 -3.21008 -0.73852
H 0.82602 -2.42756 -1.05878
H 0.97550 -3.97420 -0.17689
H 1.92790 -3.67696 -1.66183
C 3.63159 -3.74972 0.59231
H 3.07814 -4.45580 1.23601
H 4.47756 -3.34697 1.17376
H 4.04784 -4.33636 -0.24560
C 3.10664 2.29774 -1.35123
H 2.43786 2.40335 -0.48035
C 2.27077 2.59389 -2.61904
H 2.88185 2.48124 -3.53216
H 1.87211 3.62405 -2.60220
H 1.41643 1.89941 -2.71075
C 4.24965 3.32408 -1.20575
H 4.86228 3.12366 -0.31067
H 3.84322 4.34738 -1.12036
H 4.92558 3.31329 -2.07922
Mg -0.00005 0.00043 0.18738

9^{Na}

SCF (BP86) Energy = -2583.71039948
Enthalpy 0K = -2582.279802
Enthalpy 298K = -2582.183208
Free Energy 298K = -2582.408854
Lowest Frequency = 25.7364 cm^{-1}
Second Frequency = 28.7482 cm^{-1}
SCF (BP86-D3BJ) Energy = -2584.21464000
SCF (Benzene) Energy = -2583.72981115
SCF (BS2) Energy = -4449.50396798

Si -4.45184 1.03190 -1.67121
Si -4.45163 -1.03146 1.67150
Si 4.45180 1.03159 1.67122
Si 4.45149 -1.03176 -1.67146
Mg -1.67056 0.00005 -0.00001
Mg 1.67047 0.00003 -0.00000
Na 0.00006 3.57971 -0.00018
Na -0.00005 -3.58080 -0.00021
N -2.87354 1.46130 -0.96293
N -2.87352 -1.46113 0.96294
N 2.87338 -1.46138 -0.96285
N 2.87361 1.46121 0.96280

C	-2.38599	2.75195	-1.23361
C	2.38620	2.75191	1.23356
C	-1.49473	-3.00862	2.35218
C	-2.74038	3.89500	-0.41100
C	-2.38612	-2.75182	1.23371
C	-1.49480	3.00877	-2.35223
C	2.38596	-2.75204	-1.23364
C	1.49501	3.00877	2.35216
C	2.74074	3.89497	0.41102
C	2.74058	-3.89514	-0.41119
C	-0.99752	4.30977	-2.58654
H	-0.34223	4.47888	-3.44896
C	-1.14036	1.90911	-3.35358
H	-1.47784	0.96016	-2.90055
C	5.83195	-0.66617	-0.38945
H	5.85852	-1.52031	0.31493
H	6.77321	-0.75224	-0.97342
C	-2.74090	-3.89495	0.41137
C	-2.21246	5.17458	-0.69148
H	-2.51012	6.01981	-0.05906
C	-0.99762	-4.30967	2.58661
H	-0.34218	-4.47873	3.44893
C	5.83207	0.66584	0.38905
H	5.85872	1.52000	-0.31530
H	6.77340	0.75176	0.97296
C	-5.83209	0.66640	-0.38892
H	-5.85854	1.52051	0.31550
H	-6.77346	0.75248	-0.97272
C	1.14047	1.90913	3.35349
H	1.47796	0.96017	2.90051
C	1.49473	-3.00886	-2.35223
C	-5.83206	-0.66567	0.38949
H	-5.85874	-1.51976	-0.31495
H	-6.77331	-0.75170	0.97349
C	0.99781	4.30980	2.58650
H	0.34250	4.47892	3.44889
C	-1.33890	5.39826	-1.76752
H	-0.94971	6.40013	-1.97579
C	2.21290	-5.17477	-0.69184
H	2.51082	-6.02006	-0.05961
C	3.72430	3.76481	-0.75086
H	3.98977	2.69557	-0.80357
C	-2.21314	-5.17458	0.69198
H	-2.51112	-6.01987	0.05978
C	1.33932	-5.39846	-1.76787
H	0.95040	-6.40041	-1.97631
C	2.21293	5.17457	0.69154
H	2.51070	6.01980	0.05916
C	-3.72460	-3.76491	-0.75040
H	-3.99020	-2.69571	-0.80309
C	-1.13984	-1.90888	3.35328
H	-1.47749	-0.95996	2.90033
C	3.72408	-3.76502	0.75074
H	3.98943	-2.69575	0.80359
C	0.99766	-4.30992	-2.58669
H	0.34235	-4.47902	-3.44910
C	1.33932	5.39828	1.76753
H	0.95025	6.40019	1.97584
C	4.26324	0.48766	-2.81657
H	3.70870	0.21706	-3.73127
H	5.24900	0.88291	-3.12151
H	3.71380	1.30452	-2.32096
C	-1.33939	-5.39824	1.76787
H	-0.95040	-6.40017	1.97626
C	-4.26396	-0.48761	-2.81625
H	-3.70910	-0.21719	-3.73081
H	-5.24981	-0.88245	-3.12145
H	-3.71496	-1.30471	-2.32053
C	-5.17458	2.45537	-2.73884
H	-5.86558	3.08610	-2.15432
H	-5.75547	2.02896	-3.57588
H	-4.40360	3.11699	-3.16460
C	4.26368	-0.48772	2.81649
H	3.70889	-0.21702	3.73101
H	5.24946	-0.88268	3.12175
H	3.71452	-1.30482	2.32096
C	-3.72394	3.76491	0.75087
H	-3.98928	2.69565	0.80372
C	5.17472	2.45508	2.73868
H	5.86546	3.08592	2.15395
H	5.75594	2.02873	3.57551
H	4.40380	3.11659	3.16470
C	-1.89971	2.10500	-4.68787
H	-2.99064	2.12758	-4.54025
H	-1.66715	1.28390	-5.38959
H	-1.60674	3.05321	-5.17419
C	1.13995	-1.90914	-3.35340
H	1.47756	-0.96022	-2.90044
C	-4.26332	0.48786	2.81673
H	-3.70844	0.21722	3.73121
H	-5.24905	0.88289	3.12206
H	-3.71418	1.30490	2.32108
C	0.37175	1.80689	-3.62903
H	0.77893	2.73105	-4.07654
H	0.58378	0.98767	-4.33702
H	0.93477	1.59551	-2.70170
C	1.89970	2.10504	4.68785
H	2.99064	2.12764	4.54033
H	1.66710	1.28393	5.38955
H	1.60667	3.05324	5.17415
C	0.37240	-1.80671	3.62804
H	0.77970	-2.73082	4.07550
H	0.58480	-0.98738	4.33579
H	0.93500	-1.59550	2.70040
C	-0.37167	1.80696	3.62883
H	-0.77885	2.73114	4.07628
H	-0.58378	0.98776	4.33682
H	-0.93462	1.59556	2.70145
C	-5.17449	-2.45484	2.73908
H	-5.86351	-3.08713	2.15392
H	-5.75752	-2.02834	3.57457
H	-4.40341	-3.11497	3.16697
C	-0.37227	-1.80698	-3.62829
H	-0.77957	-2.73117	-4.07561
H	-0.58460	-0.98778	-4.33621
H	-0.93493	-1.59559	-2.70075
C	5.17425	-2.45512	-2.73913
H	5.86331	-3.08744	-2.15405
H	5.75724	-2.02859	-3.57464
H	4.40315	-3.11523	-3.16699
C	3.10209	-4.17901	2.10181
H	2.19766	-3.59212	2.33575
H	3.82034	-4.01923	2.92494
H	2.82277	-5.24852	2.10903
C	5.01744	4.57462	-0.50291
H	4.81099	5.65986	-0.46925

```
H   5.74414  4.39925 -1.31564
H   5.49466  4.29711  0.44989
C  -5.01717  4.57452  0.50272
H  -4.81082  5.65978  0.46879
H  -5.74385  4.39928  1.31550
H  -5.49436  4.29671 -0.45000
C   3.10231  4.17855 -2.10201
H   2.19798  3.59150 -2.33592
H   3.82062  4.01875 -2.92509
H   2.82284  5.24801 -2.10938
C  -3.10209  4.17895  2.10199
H  -2.19762  3.59214  2.33600
H  -3.82040  4.01908  2.92505
H  -2.82287  5.24848  2.10927
C  -3.10264 -4.17856 -2.10159
H  -2.19840 -3.59138 -2.33554
H  -3.82101 -4.01887 -2.92463
H  -2.82303 -5.24799 -2.10896
C  -1.89853 -2.10463  4.68796
H  -2.98954 -2.12708  4.54092
H  -1.66553 -1.28352  5.38952
H  -1.60542 -3.05284  5.17419
C   1.89874 -2.10494 -4.68803
H   2.98974 -2.12729 -4.54092
H   1.66571 -1.28389 -5.38965
H   1.60573 -3.05321 -5.17420
C   5.01733 -4.57467  0.50282
H   4.81097 -5.65993  0.46897
H   5.74390 -4.39937  1.31568
H   5.49468 -4.29697 -0.44985
C  -5.01763 -4.57490 -0.50245
H  -4.81105 -5.66013 -0.46885
H  -5.74436 -4.39958 -1.31516
H  -5.49490 -4.29750  0.45036
```

9^{K}

SCF (BP86) Energy = -2639.95287279
Enthalpy 0K = -2638.523787
Enthalpy 298K = -2638.426088
Free Energy 298K = -2638.660050
Lowest Frequency = 13.6134 cm^{-1}
Second Frequency = 13.9704 cm^{-1}
SCF (BP86-D3BJ) Energy = -2640.43455728
SCF (Benzene) Energy = -2639.96789567
SCF (BS2) Energy = -5324.87647968

```
K  -0.00004 -3.09402 -0.00126
K   0.00009  3.09369 -0.00109
Si  5.22308 -1.29686  1.53822
Si  5.22376  1.29620 -1.53756
Si -5.22295  1.29713  1.53859
Si -5.22389 -1.29588 -1.53722
N   3.57884 -1.52555  0.88071
N   3.57929  1.52499 -0.88064
N  -3.57868  1.52563  0.88106
N  -3.57944 -1.52489 -0.88033
C   6.51684 -0.73489  0.23977
H   7.48784 -0.96233  0.72879
H   6.46611 -1.42582 -0.62399
C   6.51700  0.73408 -0.23866
H   7.48819  0.96142 -0.72735
H   6.46605  1.42502  0.62509
C   5.90839 -2.94742  2.21572
H   5.21547 -3.44565  2.91216
H   6.85802 -2.76653  2.74916
H   6.11577 -3.65634  1.39595
C   5.24670 -0.00421  2.94909
H   4.74615  0.93153  2.64634
H   6.28377  0.24988  3.23270
H   4.73369 -0.38212  3.84933
C   5.24797  0.00367 -2.94855
H   4.74737 -0.93214 -2.64612
H   6.28518 -0.25030 -3.23176
H   4.73530  0.38164 -3.84897
C   5.90940  2.94674 -2.21480
H   5.21675  3.44495 -2.91153
H   6.85923  2.76581 -2.74786
H   6.11646  3.65568 -1.39497
C  -6.51678  0.73528  0.24016
H  -7.48775  0.96279  0.72921
H  -6.46601  1.42622 -0.62359
C  -6.51706 -0.73369 -0.23828
H  -7.48828 -0.96095 -0.72695
H  -6.46613 -1.42464  0.62545
C  -5.90803  2.94779  2.21610
H  -5.21513  3.44578  2.91273
H  -6.85780  2.76705  2.74934
H  -6.11508  3.65683  1.39636
C  -5.24678  0.00454  2.94953
H  -4.74640 -0.93131  2.64684
H  -6.28390 -0.24934  3.23315
H  -4.73370  0.38242  3.84975
C  -5.24790 -0.00325 -2.94811
H  -4.74711  0.93244 -2.64561
H  -6.28506  0.25094 -3.23129
H  -4.73531 -0.38124 -3.84857
C  -5.90976 -2.94631 -2.21450
H  -5.21710 -3.44473 -2.91106
H  -6.85945 -2.76520 -2.74775
H  -6.11714 -3.65514 -1.39466
C   3.00734 -2.80629  1.06201
C   2.28610 -3.15501  2.26425
C   1.71493 -4.43862  2.39819
H   1.18681 -4.68963  3.32657
C   1.82226 -5.40776  1.38867
H   1.38832 -6.40453  1.52219
C   2.52231 -5.08262  0.21576
H   2.63077 -5.83948 -0.57088
C   3.11837 -3.81714  0.03599
C   2.13413 -2.16407  3.41969
H   2.63156 -1.23355  3.09504
C   2.82463 -2.66333  4.70938
H   2.36158 -3.59559  5.08019
H   2.73664 -1.90846  5.51079
H   3.89618 -2.86464  4.54878
C   0.65459 -1.83008  3.70782
H   0.17308 -1.36219  2.82983
H   0.57437 -1.11574  4.54590
H   0.07736 -2.73090  3.98555
C   3.88883 -3.53756 -1.25423
H   4.39791 -2.57086 -1.09531
C   2.93746 -3.37205 -2.46099
H   2.34970 -4.29212 -2.63808
H   3.50129 -3.15375 -3.38520
H   2.22764 -2.53674 -2.30856
C   4.96161 -4.60570 -1.55496
```

```
H   5.65000 -4.73515 -0.70334
H   5.55860 -4.31268 -2.43621
H   4.51402 -5.59171 -1.77513
C  -3.00824 -2.80570 -1.06209
C  -2.28758 -3.15432 -2.26471
C  -1.71674 -4.43803 -2.39915
H  -1.18911 -4.68898 -3.32782
C  -1.82380 -5.40732 -1.38975
H  -1.39012 -6.40416 -1.52366
C  -2.52325 -5.08226 -0.21646
H  -2.63154 -5.83927  0.57006
C  -3.11902 -3.81671 -0.03621
C  -3.88898 -3.53725  1.25433
H  -4.39810 -2.57053  1.09570
C  -2.93713 -3.37185  2.46072
H  -3.50058 -3.15354  3.38515
H  -2.22729 -2.53662  2.30801
H  -2.34937 -4.29197  2.63753
C  -4.96166 -4.60540  1.55538
H  -5.65035 -4.73482  0.70400
H  -5.55834 -4.31241  2.43685
H  -4.51400 -5.59142  1.77537
C  -2.13587 -2.16317 -3.41999
H  -2.63296 -1.23261 -3.09493
C  -0.65638 -1.82942 -3.70869
H  -0.17442 -1.36171 -2.83085
H  -0.57637 -1.11498 -4.54670
H  -0.07943 -2.73031 -3.98678
C  -2.82702 -2.66200 -4.70950
H  -2.73921 -1.90698 -5.51079
H  -3.89855 -2.86313 -4.54849
H  -2.36432 -3.59428 -5.08070
C   3.00789  2.80568 -1.06262
C   2.28701  3.15392 -2.26522
C   1.71598  4.43752 -2.39988
H   1.18818  4.68818 -3.32853
C   1.82305  5.40706 -1.39072
H   1.38922  6.40381 -1.52480
C   2.52272  5.08238 -0.21746
H   2.63101  5.83958  0.56887
C   3.11868  3.81695 -0.03699
C   2.13534  2.16250 -3.42027
H   2.63248  1.23205 -3.09501
C   0.65587  1.82864 -3.70891
H   0.17390  1.36117 -2.83096
H   0.57590  1.11397 -4.54673
H   0.07890  2.72943 -3.98727
C   2.82650  2.66110 -4.70987
H   2.73875  1.90591 -5.51101
H   3.89802  2.86230 -4.54886
H   2.36378  3.59329 -5.08126
C   3.88878  3.53783  1.25354
H   4.39829  2.57131  1.09494
C   2.93697  3.37203  2.45992
H   2.34879  4.29189  2.63669
H   3.50049  3.15399  3.38438
H   2.22752  2.53647  2.30721
C   4.96101  4.60642  1.55463
H   5.64962  4.73619  0.70324
H   5.55783  4.31362  2.43607
H   4.51294  5.59223  1.77472
C  -3.00700  2.80625  1.06262
C  -2.28559  3.15457  2.26488
C  -1.71425  4.43808  2.39908
H  -1.18601  4.68878  3.32747
C  -1.82157  5.40748  1.38982
H  -1.38750  6.40417  1.52355
C  -2.52178  5.08273  0.21690
H  -2.63022  5.83981 -0.56953
C  -3.11802  3.81737  0.03687
C  -3.88857  3.53815 -1.25337
H  -4.39804  2.57164 -1.09451
C  -2.93721  3.37227 -2.46009
H  -2.34904  4.29209 -2.63710
H  -3.50107  3.15425 -3.38434
H  -2.22776  2.53664 -2.30766
C  -4.96090  4.60672 -1.55415
H  -5.64924  4.73650 -0.70254
H  -5.55801  4.31390 -2.43539
H  -4.51291  5.59253 -1.77438
C  -2.13366  2.16334  3.42006
H  -2.63115  1.23293  3.09519
C  -0.65414  1.82921  3.70812
H  -0.17264  1.36155  2.83001
H  -0.57396  1.11463  4.54600
H  -0.07688  2.72993  3.98611
C  -2.82415  2.66233  4.70986
H  -2.36107  3.59449  5.08089
H  -2.73622  1.90727  5.51110
H  -3.89570  2.86373  4.54928
Mg -2.51798  0.00019  0.00023
Mg  2.51798 -0.00017 -0.00003
```

Na(0)

SCF (BP86) Energy = -0.190251935461
Enthalpy 0K = -0.190252
Enthalpy 298K = -0.187891
Free Energy 298K = -0.205335
SCF (BP86-D3BJ) Energy = -0.19025193546
SCF (Benzene) Energy = -0.194926402159
SCF (BS2) Energy = -162.278274773

```
Na  0.10000  0.10000  0.10000
```

K(0)

SCF (BP86) Energy = -28.3228762473
Enthalpy 0K = -28.322876
Enthalpy 298K = -28.320516
Free Energy 298K = -28.338707
SCF (BP86-D3BJ) Energy = -28.3228762473
SCF (Benzene) Energy = -28.3255799042
SCF (BS2) Energy = -599.963524099

```
K  0.10000  0.10000  0.10000
```

Mechanism Geometries

CO

SCF (BP86) Energy = -113.305193181
Enthalpy 0K = -113.300371
Enthalpy 298K = -113.297066
Free Energy 298K = -113.319529
Lowest Frequency = 2116.8425 cm^{-1}
SCF (BP86-D3BJ) Energy = -113.305815826
SCF (Benzene) Energy = -113.305476544

SCF (BS2) Energy = -113.346138184

O	0.00000	0.00000	0.49281
C	-0.00000	-0.00000	-0.65708

9 + CO

SCF (BP86) Energy = -2697.01734616
Enthalpy 0K = -2695.581923
Enthalpy 298K = -2695.480688
Free Energy 298K = -2695.724203
Lowest Frequency = 6.3351 cm^{-1}
Second Frequency = 6.9745 cm^{-1}
SCF (BP86-D3BJ) Energy = -2697.52457539
SCF (Benzene) Energy = -2697.03333877
SCF (BS2) Energy = -4562.84957490

Si	2.17975	-4.04077	-1.64232
Si	3.93489	-2.64077	1.58023
Si	-3.35522	2.88441	1.80587
Si	-1.92854	4.12602	-1.63199
Mg	-0.85739	1.36863	0.05675
Na	-2.56431	-2.22028	0.20304
Na	2.95726	2.36347	-0.18754
N	0.87628	-3.08627	-0.88932
N	3.21665	-1.17162	0.87116
N	-0.55481	3.20222	-0.97071
N	-2.70572	1.38295	1.09898
C	-0.43680	-3.54799	-1.08926
C	-3.36865	0.18806	1.43230
C	3.58866	0.91451	2.18712
C	-1.04708	-4.53142	-0.21205
C	3.90680	0.03594	1.07437
C	-1.25485	-3.06191	-2.18723
C	0.73123	3.64959	-1.32085
C	-2.93649	-0.63277	2.55038
C	-4.51357	-0.29197	0.67920
C	1.42368	4.67203	-0.55649
C	-2.58072	-3.52075	-2.34957
H	-3.16905	-3.15013	-3.19721
C	-0.68751	-2.11243	-3.24309
H	0.28913	-1.77069	-2.85772
C	-3.02480	4.98657	-0.31237
H	-2.34974	5.55987	0.35239
H	-3.58261	5.75903	-0.88372
C	4.96892	0.47306	0.18581
C	-2.37797	-4.95483	-0.42162
H	-2.80554	-5.70792	0.25146
C	4.27642	2.13680	2.35290
H	4.02861	2.77167	3.21148
C	-4.01602	4.15276	0.52641
H	-4.72046	3.61445	-0.13712
H	-4.65677	4.83417	1.12603
C	3.40871	-4.83647	-0.40131
H	2.80620	-5.38264	0.35031
H	3.91298	-5.63139	-0.99137
C	-1.81697	-0.17489	3.48569
H	-1.33749	0.68929	2.99229
C	1.43910	3.10530	-2.46714
C	4.47165	-3.96617	0.30018
H	5.10569	-3.45727	-0.45172
H	5.17091	-4.62012	0.86395
C	-3.60142	-1.84250	2.84843
H	-3.26641	-2.43308	3.70923
C	-3.15837	-4.45772	-1.47760
H	-4.18460	-4.80708	-1.62993
C	2.72668	5.08032	-0.91667
H	3.21764	5.86333	-0.32606
C	-5.10377	0.51812	-0.47413
H	-4.46416	1.41067	-0.57747
C	5.62392	1.70603	0.39980
H	6.43119	2.00142	-0.28140
C	3.39970	4.53041	-2.01925
H	4.40500	4.86943	-2.28954
C	-5.14166	-1.51087	1.02028
H	-6.01002	-1.84447	0.43967
C	5.44573	-0.39953	-0.97423
H	4.78987	-1.28635	-0.97559
C	2.57550	0.50393	3.25566
H	2.03977	-0.37300	2.85085
C	0.75880	5.36633	0.63145
H	-0.23107	4.89307	0.74385
C	2.74114	3.55280	-2.78252
H	3.24448	3.13887	-3.66414
C	-4.69551	-2.29755	2.09460
H	-5.20169	-3.23380	2.35158
C	-3.03547	2.98427	-2.69234
H	-2.52205	2.70500	-3.62824
H	-3.98421	3.48289	-2.96086
H	-3.28574	2.05455	-2.15583
C	5.28740	2.55010	1.46998
H	5.81459	3.49695	1.62538
C	3.16644	-2.95627	-2.86977
H	2.56082	-2.73442	-3.76503
H	4.08830	-3.46682	-3.20214
H	3.46051	-1.99500	-2.41783
C	1.49615	-5.53360	-2.63660
H	1.42877	-6.43939	-2.01069
H	2.18460	-5.76503	-3.46870
H	0.49697	-5.35327	-3.06378
C	-2.01784	3.74044	2.87087
H	-1.82709	3.16407	3.79215
H	-2.33253	4.75773	3.16592
H	-1.06280	3.82993	2.32792
C	-0.26488	-5.17105	0.93425
H	0.73128	-4.69787	0.92199
C	-4.85836	2.54820	2.95190
H	-5.81142	2.64131	2.40435
H	-4.87673	3.29662	3.76391
H	-4.83891	1.54801	3.41321
C	-0.44857	-2.84535	-4.58505
H	0.22865	-3.70586	-4.46842
H	-0.00228	-2.15833	-5.32633
H	-1.39862	-3.22018	-5.00730
C	0.77593	2.09782	-3.40693
H	-0.15026	1.76956	-2.90305
C	2.70593	-3.44088	2.80696
H	2.60792	-2.82290	3.71581
H	3.04548	-4.44629	3.11494
H	1.70208	-3.54730	2.36447
C	-1.57644	-0.87658	-3.47847
H	-2.58142	-1.14915	-3.84690
H	-1.12698	-0.21058	-4.23465
H	-1.69520	-0.28898	-2.54978
C	-2.38558	0.29879	4.84499
H	-3.11643	1.11341	4.72305
H	-1.57293	0.66444	5.49814

```
H  -2.89130 -0.52995  5.37305
C   1.54406  1.60424  3.56846
H   2.01617  2.51893  3.96920
H   0.82330  1.25700  4.32822
H   0.96665  1.88012  2.66725
C  -0.74704 -1.25772  3.72086
H  -1.16562 -2.16230  4.19677
H   0.04722 -0.88267  4.38848
H  -0.26615 -1.55709  2.77139
C   5.54275 -2.25648  2.56006
H   6.44084 -2.47231  1.95669
H   5.59368 -2.89965  3.45644
H   5.61116 -1.20817  2.89173
C   1.65165  0.85801 -3.66964
H   2.60562  1.11693 -4.16253
H   1.13126  0.14524 -4.33174
H   1.88145  0.32636 -2.72797
C  -1.34895  5.56195 -2.76534
H  -1.18590  6.48977 -2.19173
H  -2.13264  5.77632 -3.51357
H  -0.41518  5.34122 -3.30653
C   1.54391  5.16605  1.94576
H   1.67897  4.09771  2.18515
H   1.00765  5.62841  2.79291
H   2.54564  5.63067  1.89429
C  -6.54553  0.98575 -0.17092
H  -7.23513  0.12639 -0.08671
H  -6.91895  1.63515 -0.98237
H  -6.60058  1.55043  0.77296
C  -0.08252 -6.69246  0.73086
H  -1.05242 -7.22139  0.76281
H   0.55252 -7.11436  1.52966
H   0.38888 -6.92054 -0.23791
C  -5.07888 -0.25469 -1.81061
H  -4.05729 -0.56781 -2.08572
H  -5.46119  0.37675 -2.63185
H  -5.71090 -1.16023 -1.76485
C  -0.91214 -4.90458  2.31043
H  -1.02113 -3.82542  2.51165
H  -0.29324 -5.32984  3.11985
H  -1.91444 -5.36532  2.38271
C   5.31245  0.30575 -2.34117
H   4.27535  0.62518 -2.54029
H   5.61049 -0.37366 -3.15880
H   5.95958  1.19989 -2.40172
C   3.28993  0.07330  4.55939
H   4.00150 -0.74869  4.38476
H   2.55381 -0.26518  5.31057
H   3.85249  0.91697  4.99892
C   0.38701  2.75603 -4.75231
H  -0.29245  3.61075 -4.60955
H  -0.11868  2.02496 -5.40843
H   1.28132  3.12283 -5.28808
C   0.54954  6.87638  0.37612
H   1.51528  7.40552  0.28219
H  -0.00114  7.33615  1.21560
H  -0.02085  7.05877 -0.54814
C   6.90299 -0.87419 -0.77197
H   7.60768 -0.02295 -0.79022
H   7.19834 -1.56821 -1.57854
H   7.03175 -1.39218  0.19120
Mg  1.27227 -1.20535  0.01530
C  -8.24188 -3.14142 -0.82917
O  -9.22861 -3.50979 -1.28736
```

TS(9-A)

SCF (BP86) Energy = -2696.97073157
Enthalpy 0K = -2695.535776
Enthalpy 298K = -2695.436216
Free Energy 298K = -2695.670109
Lowest Frequency = -56.7379 cm^{-1}
Second Frequency = 18.3382 cm^{-1}
SCF (BP86-D3BJ) Energy = -2697.49286289
SCF (Benzene) Energy = -2696.98925909
SCF (BS2) Energy = -4562.80245684

```
Si  4.45380 -1.00689 -1.68040
Si  4.56011  0.89764  1.74490
Si -4.62482 -0.73294  1.46020
Si -4.38252  1.39844 -1.86987
Mg -1.72327  0.24624  0.02035
Na -0.39375 -5.02503 -0.24881
Na  0.17665  3.43626  0.07974
N   2.87671 -1.47443 -0.98257
N   3.00224  1.40328  1.05171
N  -2.82558  1.69922 -1.06635
N  -3.00143 -1.26824  0.91668
C   2.45411 -2.77706 -1.30436
C  -2.72764 -2.60154  1.27801
C   1.64426  2.92395  2.49273
C   2.85539 -3.93630 -0.52873
C   2.53981  2.69453  1.37368
C   1.58360 -3.02646 -2.43825
C  -2.23321  2.95837 -1.29454
C  -2.11672 -2.94612  2.55000
C  -3.06232 -3.71039  0.40493
C  -2.50461  4.09195 -0.43292
C   1.15904 -4.33367 -2.74712
H   0.53627 -4.49167 -3.63711
C   1.14278 -1.88891 -3.36090
H   1.55502 -0.96328 -2.92245
C  -5.82216  1.03667 -0.65935
H  -5.85375  1.86159  0.07875
H  -6.73900  1.16693 -1.27299
C   2.90775  3.85628  0.58749
C   2.40887 -5.22777 -0.88386
H   2.77366 -6.08808 -0.30682
C   1.14629  4.21912  2.75370
H   0.48137  4.36819  3.61216
C  -5.87189 -0.32780  0.05603
H  -5.81952 -1.14878 -0.68448
H  -6.86031 -0.45235  0.54762
C   5.86704 -0.76244 -0.40303
H   5.87191 -1.64953  0.25837
H   6.79846 -0.85042 -1.00195
C  -1.71188 -1.87231  3.56651
H  -1.84553 -0.89918  3.06128
C  -1.30652  3.17926 -2.38865
C   5.92225  0.52677  0.44351
H   5.98416  1.41367 -0.21710
H   6.86775  0.54337  1.02683
C  -1.91627 -4.29571  2.90666
H  -1.47549 -4.52222  3.88471
C   1.56502 -5.45058 -1.98931
H   1.30144 -6.46928 -2.30223
C  -1.85187  5.32650 -0.64184
```

H	-2.08059	6.16626	0.02558
C	-3.71314	-3.48077	-0.95888
H	-3.71823	-2.38680	-1.10853
C	2.37324	5.12774	0.88933
H	2.67320	5.98688	0.27709
C	-0.93995	5.51362	-1.69173
H	-0.45033	6.48097	-1.84385
C	-2.85172	-5.04602	0.81805
H	-3.21027	-5.86252	0.17342
C	3.90525	3.75726	-0.56576
H	4.19792	2.69577	-0.62494
C	1.27202	1.80079	3.46211
H	1.58604	0.85790	2.97858
C	-3.52674	4.00376	0.69920
H	-3.92589	2.97614	0.66573
C	-0.68837	4.43736	-2.55669
H	-0.00032	4.58137	-3.39779
C	-2.27718	-5.35686	2.06453
H	-2.16534	-6.39734	2.39048
C	-4.25539	-0.07673	-3.08404
H	-3.64615	0.19012	-3.96418
H	-5.25401	-0.38417	-3.44344
H	-3.78534	-0.95402	-2.60824
C	1.48970	5.32490	1.96093
H	1.09232	6.32017	2.18509
C	4.25504	0.59136	-2.70838
H	3.70028	0.38201	-3.63912
H	5.23772	1.01371	-2.98531
H	3.70352	1.36931	-2.15629
C	5.12910	-2.33259	-2.88687
H	5.49050	-3.23630	-2.36996
H	5.98212	-1.90643	-3.44435
H	4.37566	-2.65904	-3.62088
C	-4.47318	0.78412	2.61104
H	-4.05176	0.49070	3.58729
H	-5.45919	1.24885	2.79095
H	-3.81427	1.55527	2.18081
C	3.81853	-3.81553	0.65444
H	3.99828	-2.73525	0.78559
C	-5.54395	-2.09968	2.44468
H	-5.96575	-2.86999	1.77741
H	-6.38982	-1.63610	2.98342
H	-4.91597	-2.62100	3.18279
C	1.71141	-2.04420	-4.79088
H	2.81208	-2.08515	-4.79514
H	1.40274	-1.19194	-5.42228
H	1.33936	-2.96549	-5.27506
C	-1.02198	2.09154	-3.42521
H	-1.45721	1.16091	-3.01944
C	4.32003	-0.64519	2.85017
H	3.80776	-0.37095	3.78826
H	5.28672	-1.10991	3.11540
H	3.70662	-1.41002	2.34597
C	-0.39030	-1.74546	-3.43340
H	-0.86451	-2.64432	-3.86681
H	-0.67195	-0.89087	-4.07150
H	-0.82780	-1.57072	-2.43463
C	-2.61714	-1.89304	4.82181
H	-3.67785	-1.73897	4.57196
H	-2.31550	-1.09622	5.52480
H	-2.53404	-2.85673	5.35649
C	-0.24057	1.72430	3.74512
H	-0.62755	2.64827	4.21049
H	-0.46277	0.89746	4.44037
H	-0.80792	1.54135	2.81389
C	-0.23805	-1.99553	4.01452
H	-0.02930	-2.97513	4.48007
H	0.00116	-1.22202	4.76413
H	0.46579	-1.87236	3.17447
C	5.34053	2.26799	2.83908
H	6.00600	2.92000	2.24812
H	5.95686	1.80629	3.63082
H	4.59169	2.91370	3.32466
C	0.48357	1.86191	-3.65921
H	0.98582	2.75961	-4.06184
H	0.64412	1.04932	-4.38903
H	0.99028	1.57376	-2.71967
C	-4.99022	2.92106	-2.86513
H	-5.56712	3.60621	-2.22076
H	-5.66324	2.59220	-3.67677
H	-4.17239	3.50473	-3.31600
C	-2.88723	4.23030	2.08610
H	-2.08287	3.50215	2.29055
H	-3.64022	4.12327	2.88666
H	-2.45365	5.24362	2.17012
C	-5.17283	-3.98655	-1.00954
H	-5.22225	-5.08095	-0.86137
H	-5.62772	-3.76048	-1.99005
H	-5.79363	-3.51667	-0.23144
C	5.17522	-4.50092	0.36740
H	5.05164	-5.59062	0.22891
H	5.86797	-4.35061	1.21427
H	5.65739	-4.10207	-0.53857
C	-2.90982	-4.13045	-2.10713
H	-1.86968	-3.76030	-2.14592
H	-3.36131	-3.89453	-3.08658
H	-2.89879	-5.23522	-2.02277
C	3.24076	-4.37406	1.97446
H	2.29179	-3.88654	2.24857
H	3.95652	-4.21674	2.80001
H	3.05481	-5.46270	1.90659
C	3.28262	4.16347	-1.91907
H	2.40121	3.54702	-2.16759
H	4.01307	4.03934	-2.73775
H	2.96310	5.22161	-1.91534
C	2.04405	1.94064	4.79628
H	3.13442	1.94393	4.64197
H	1.79783	1.10501	5.47576
H	1.77541	2.88145	5.31032
C	-1.71224	2.40551	-4.77449
H	-2.80204	2.51847	-4.66461
H	-1.52517	1.59550	-5.50243
H	-1.32017	3.34241	-5.21026
C	-4.70170	4.98654	0.49391
H	-4.36490	6.03751	0.55286
H	-5.46817	4.83964	1.27522
H	-5.18135	4.84602	-0.48803
C	5.17636	4.59586	-0.30045
H	4.94685	5.67648	-0.26800
H	5.91775	4.43795	-1.10359
H	5.64662	4.32716	0.65879
Mg	1.73738	0.10365	-0.04163
C	0.30742	-2.96275	1.02533
O	0.04178	-2.13226	0.25373

A

SCF (BP86) Energy = -2697.00466261
Enthalpy 0K = -2695.567347
Enthalpy 298K = -2695.467564
Free Energy 298K = -2695.701713
Lowest Frequency = 18.1060 cm^{-1}
Second Frequency = 21.5004 cm^{-1}
SCF (BP86-D3BJ) Energy = -2697.52170398
SCF (Benzene) Energy = -2697.01983454
SCF (BS2) Energy = -4562.83424098

```
Si -4.96938  0.77035 -1.13725
Si -4.21156 -1.48325  2.06553
Si  4.45066  1.18616  1.69453
Si  4.61951 -0.76690 -1.70899
Mg  1.75348  0.13603 -0.06105
Na -0.17933  3.43101  0.06288
Na  0.25888 -3.58148 -0.18335
N  -3.26131  1.20140 -0.83704
N  -2.73036 -1.71815  1.09887
N   3.01938 -1.23855 -1.06115
N   2.86997  1.60366  0.97613
C  -2.83952  2.50446 -1.18963
C   2.28725  2.83697  1.33235
C  -1.14549 -3.26029  2.26174
C  -2.95199  3.60849 -0.25694
C  -2.11990 -2.97998  1.22255
C  -2.28234  2.80817 -2.49694
C   2.58588 -2.54103 -1.37574
C   1.40138  2.95272  2.47478
C   2.53168  4.04944  0.56974
C   3.02740 -3.70775 -0.63036
C  -1.72486  4.07923 -2.74513
H  -1.28088  4.28094 -3.72584
C  -2.40514  1.82067 -3.66428
H  -2.47807  0.81001 -3.22280
C   5.94322 -0.41838 -0.36257
H   5.97469 -1.27357  0.33851
H   6.90908 -0.45991 -0.90923
C  -2.44588 -4.06835  0.32048
C  -2.40199  4.87345 -0.57051
H  -2.50900  5.69404  0.15014
C  -0.56943 -4.54575  2.36906
H   0.14393 -4.74049  3.17855
C   5.86068  0.91086  0.42077
H   5.85425  1.76849 -0.27904
H   6.78660  1.03857  1.02092
C  -5.98401  0.23071  0.40109
H  -5.88259  1.01878  1.17296
H  -7.03060  0.32547  0.03974
C   1.20730  1.79109  3.45070
H   1.65267  0.90341  2.96841
C   1.67438 -2.78366 -2.47934
C  -5.78365 -1.16363  1.02351
H  -5.84286 -1.94912  0.24566
H  -6.62124 -1.38521  1.71877
C   0.77907  4.18461  2.77531
H   0.12367  4.24675  3.65170
C  -1.75167  5.11013 -1.79131
H  -1.32302  6.09219 -2.01749
C   2.56931 -4.99775 -0.98050
H   2.93510 -5.86040 -0.41086
C   3.53507  4.07532 -0.58283
H   3.69576  3.02052 -0.86852
C  -1.83432 -5.33246  0.47011
H  -2.11677 -6.14214 -0.21366
C   1.67816 -5.20871 -2.04446
H   1.34552 -6.21843 -2.30616
C   1.88405  5.25440  0.91793
H   2.09231  6.15607  0.33064
C  -3.49789 -3.89640 -0.77333
H  -3.80994 -2.83908 -0.72215
C  -0.75466 -2.20258  3.29482
H  -1.21007 -1.25749  2.95142
C   4.02798 -3.59392  0.51969
H   4.16707 -2.51412  0.69569
C   1.24487 -4.09456 -2.78175
H   0.57539 -4.24860 -3.63602
C   0.99821  5.33562  2.00447
H   0.51206  6.28275  2.26068
C   4.54110  0.79932 -2.79844
H   3.88670  0.64693 -3.67224
H   5.55705  1.02676 -3.17043
H   4.18226  1.68742 -2.25581
C  -0.89948 -5.58747  1.48666
H  -0.44934 -6.57915  1.59832
C  -5.10830 -0.64799 -2.41385
H  -4.79360 -0.31931 -3.41784
H  -6.15015 -1.00915 -2.48527
H  -4.47651 -1.50616 -2.13098
C  -5.99479  2.27085 -1.77658
H  -6.68188  2.61535 -0.98457
H  -6.61973  1.97453 -2.63730
H  -5.38192  3.13247 -2.08508
C   4.30998 -0.39768  2.75941
H   3.74592 -0.19596  3.68593
H   5.31183 -0.76458  3.04809
H   3.79758 -1.21382  2.22449
C  -3.73731  3.47419  1.04980
H  -4.07100  2.42348  1.10082
C   5.09689  2.56951  2.86931
H   4.39313  3.41065  2.97682
H   6.05682  2.98005  2.51166
H   5.28164  2.15401  3.87540
C  -3.71004  2.10174 -4.45367
H  -4.60703  2.05207 -3.82296
H  -3.82611  1.37064 -5.27398
H  -3.67233  3.11011 -4.90430
C   1.23921 -1.64972 -3.40443
H   1.54424 -0.71128 -2.91115
C  -4.00289 -0.00648  3.26176
H  -3.25520 -0.22427  4.04275
H  -4.95817  0.23331  3.76250
H  -3.67307  0.90028  2.72626
C  -1.24391  1.84436 -4.67894
H  -1.18292  2.81113 -5.21075
H  -1.41948  1.07748 -5.45364
H  -0.26543  1.64709 -4.22269
C   1.96682  2.05701  4.77330
H   3.03538  2.25559  4.59984
H   1.88328  1.18747  5.44996
H   1.54472  2.93170  5.30055
C   0.77029 -2.00187  3.38585
H   1.28832 -2.91176  3.73700
H   1.01610 -1.19674  4.09851
H   1.19983 -1.71659  2.40741
C  -0.26889  1.48250  3.76024
```

```
H  -0.78632  2.33956  4.22613
H  -0.34732  0.63880  4.46742
H  -0.82103  1.19995  2.84565
C  -4.65533 -3.04232  3.09057
H  -5.26368 -3.74267  2.49305
H  -5.26425 -2.74616  3.96322
H  -3.78165 -3.60124  3.45932
C  -0.28205 -1.60793 -3.63979
H  -0.65554 -2.52470 -4.13053
H  -0.54760 -0.75990 -4.29089
H  -0.82808 -1.47716 -2.68633
C   5.34369 -2.13014 -2.85964
H   4.87248 -3.11710 -2.73073
H   6.42739 -2.24686 -2.68297
H   5.22009 -1.84062 -3.91754
C   3.51236 -4.23837  1.82449
H   2.53086 -3.83480  2.12317
H   4.21685 -4.04624  2.65244
H   3.41114 -5.33458  1.72681
C   4.89482  4.65292 -0.12076
H   4.78056  5.70577  0.19532
H   5.62772  4.62529 -0.94665
H   5.31311  4.09287  0.72811
C  -4.98573  4.38732  1.05213
H  -4.69871  5.45459  1.05358
H  -5.59310  4.20577  1.95649
H  -5.62057  4.21343  0.17020
C   3.05430  4.86735 -1.81899
H   2.04830  4.56206 -2.15163
H   3.74827  4.70955 -2.66258
H   3.02795  5.95447 -1.62250
C  -2.88473  3.76714  2.30014
H  -2.01118  3.09785  2.37012
H  -3.47819  3.62374  3.22036
H  -2.51497  4.80885  2.30564
C  -2.93978 -4.17455 -2.18551
H  -2.07787 -3.52724 -2.42234
H  -3.71291 -3.98692 -2.95120
H  -2.61473 -5.22529 -2.29608
C  -1.30898 -2.53072  4.70169
H  -2.40699 -2.60461  4.70794
H  -1.01797 -1.74496  5.42167
H  -0.90421 -3.48896  5.07503
C   1.98226 -1.73613 -4.75900
H   3.07492 -1.73915 -4.62032
H   1.71792 -0.87760 -5.40101
H   1.71112 -2.66029 -5.30111
C   5.40177 -4.20399  0.15514
H   5.31278 -5.28695 -0.04718
H   6.11168 -4.07791  0.99173
H   5.83908 -3.73098 -0.73698
C  -4.74136 -4.77719 -0.51474
H  -4.48890 -5.85216 -0.56112
H  -5.51680 -4.58272 -1.27635
H  -5.17901 -4.57990  0.47685
Mg -1.85512 -0.13922  0.01507
C   1.04560  2.18558 -1.79019
O   1.50186  1.83645 -2.80301
```

TS(A-B)

SCF (BP86) Energy = -2697.00531528
Enthalpy 0K = -2695.568354
Enthalpy 298K = -2695.469607
Free Energy 298K = -2695.700932
Lowest Frequency = -72.9075 cm^{-1}
Second Frequency = 20.4058 cm^{-1}
SCF (BP86-D3BJ) Energy = -2697.51744233
SCF (Benzene) Energy = -2697.02206068
SCF (BS2) Energy = -4562.83548686

```
Si  4.42543 -1.80082 -1.81086
Si  4.99336  0.25400  1.57910
Si -4.99228 -0.11679  1.26935
Si -4.13239  2.15741 -1.91095
Mg -1.89986  0.38938  0.00475
Na -0.97003 -3.97281  0.13962
Na  0.74037  3.34000  0.21661
N   2.81149 -1.90837 -1.07540
N   3.45962  0.93902  0.97565
N  -2.58414  2.08724 -1.03188
N  -3.42107 -0.85886  0.83383
C   2.02636 -3.06174 -1.26693
C  -3.33161 -2.24944  1.07749
C   2.25193  2.47342  2.52124
C   2.11739 -4.20428 -0.38332
C   3.10689  2.24413  1.37543
C   1.08794 -3.15173 -2.36504
C  -1.76127  3.22249 -1.16043
C  -2.91668 -2.78244  2.36397
C  -3.68654 -3.21626  0.05610
C  -1.85111  4.32713 -0.22614
C   0.32240 -4.32210 -2.55462
H  -0.35219 -4.38087 -3.41715
C   0.95752 -2.01944 -3.38421
H   1.52270 -1.16692 -2.96799
C  -5.66591  1.99184 -0.77964
H  -5.57643  2.74689  0.02535
H  -6.50874  2.34612 -1.41068
C   3.57277  3.40974  0.65415
C   1.33367 -5.35516 -0.61907
H   1.45795 -6.22499  0.03877
C   1.84615  3.78380  2.85492
H   1.20031  3.93337  3.72811
C  -6.01212  0.61135 -0.18857
H  -6.05417 -0.15268 -0.98858
H  -7.04169  0.63996  0.22815
C   5.86324 -1.70290 -0.53803
H   5.71748 -2.51846  0.19659
H   6.75868 -2.00446 -1.12230
C  -2.58186 -1.86703  3.54645
H  -2.39295 -0.86510  3.11885
C  -0.78354  3.34409 -2.22509
C   6.16007 -0.37020  0.18879
H   6.27720  0.44937 -0.54675
H   7.14798 -0.44154  0.69209
C  -2.83654 -4.17526  2.56153
H  -2.52318 -4.55255  3.54144
C   0.43680 -5.43340 -1.70036
H  -0.12870 -6.35168 -1.89597
C  -1.01824  5.45824 -0.36740
H  -1.12088  6.28536  0.34550
C  -4.26080 -2.77701 -1.29039
H  -4.11411 -1.68363 -1.34175
C   3.13333  4.69823  1.02770
H   3.49212  5.56587  0.46143
C  -0.08378  5.56269 -1.41036
```

H	0.54084	6.45562	-1.51596
C	-3.59625	-4.60513	0.31219
H	-3.90647	-5.31064	-0.46882
C	4.54587	3.28172	-0.51787
H	4.85831	2.22370	-0.54357
C	1.83492	1.32398	3.43903
H	2.17102	0.39358	2.94797
C	-2.86365	4.31024	0.91751
H	-3.46488	3.39743	0.76906
C	0.01877	4.50235	-2.32493
H	0.73346	4.58135	-3.15245
C	-3.16174	-5.09942	1.55251
H	-3.11655	-6.17723	1.74353
C	-4.24012	0.78805	-3.24324
H	-3.52562	0.97304	-4.06270
H	-5.25375	0.74252	-3.68054
H	-4.01387	-0.20689	-2.82360
C	2.26623	4.90038	2.11401
H	1.94379	5.90919	2.39139
C	4.52765	-0.24059	-2.90872
H	3.85926	-0.32322	-3.78232
H	5.55598	-0.07881	-3.27893
H	4.23339	0.66034	-2.34258
C	4.82097	-3.34421	-2.87501
H	5.32545	-4.12212	-2.27709
H	5.50503	-3.06880	-3.69674
H	3.92207	-3.80093	-3.31851
C	-4.73534	1.28576	2.54264
H	-4.41877	0.88496	3.51959
H	-5.66859	1.85773	2.69372
H	-3.96090	1.99418	2.20662
C	3.09840	-4.22647	0.78684
H	3.54973	-3.22001	0.81891
C	-6.22245	-1.39729	2.01818
H	-7.01066	-1.64382	1.28611
H	-6.73034	-0.95963	2.89601
H	-5.75565	-2.34269	2.33384
C	1.59481	-2.40008	-4.74215
H	2.66078	-2.65639	-4.63917
H	1.51467	-1.55987	-5.45506
H	1.08152	-3.26917	-5.19235
C	-0.63645	2.26726	-3.30108
H	-1.23619	1.40571	-2.95697
C	4.66686	-1.19693	2.78218
H	4.30538	-0.82223	3.75501
H	5.58928	-1.77693	2.96480
H	3.90482	-1.89165	2.39130
C	-0.50147	-1.57650	-3.60538
H	-1.11858	-2.38303	-4.03864
H	-0.54653	-0.72668	-4.30751
H	-0.97076	-1.24964	-2.65880
C	-3.77148	-1.75663	4.53260
H	-4.68323	-1.37245	4.05545
H	-3.51277	-1.08336	5.36956
H	-4.00976	-2.74718	4.96123
C	0.31011	1.23119	3.64754
H	-0.09534	2.12966	4.14716
H	0.05984	0.36300	4.28059
H	-0.21839	1.10650	2.68208
C	-1.34067	-2.31117	4.35353
H	-1.52697	-3.25007	4.90601
H	-1.09785	-1.54626	5.11212
H	-0.45556	-2.46000	3.71838
C	5.99876	1.56391	2.54191
H	6.51017	2.26759	1.86486
H	6.77515	1.05942	3.14339
H	5.37053	2.15553	3.22797
C	0.82187	1.80277	-3.48139
H	1.48297	2.62577	-3.80574
H	0.88813	1.01418	-4.25022
H	1.22536	1.38335	-2.53993
C	-4.37765	3.84806	-2.77710
H	-4.66677	4.62506	-2.04894
H	-5.19510	3.76850	-3.51556
H	-3.47935	4.20963	-3.30155
C	-2.16990	4.20787	2.29374
H	-1.54888	3.29650	2.36822
H	-2.91393	4.16600	3.10872
H	-1.51702	5.08028	2.48120
C	-5.77877	-3.06253	-1.36881
H	-5.97555	-4.14945	-1.32975
H	-6.19887	-2.67758	-2.31496
H	-6.32409	-2.59561	-0.53479
C	4.23238	-5.25269	0.56018
H	3.83748	-6.28451	0.52812
H	4.96952	-5.20285	1.38098
H	4.76239	-5.07001	-0.38821
C	-3.55533	-3.42982	-2.49647
H	-2.46433	-3.27096	-2.47330
H	-3.93099	-3.00144	-3.44227
H	-3.73839	-4.51918	-2.53833
C	2.40773	-4.49563	2.14227
H	1.60795	-3.76433	2.34307
H	3.13976	-4.43285	2.96642
H	1.96500	-5.50862	2.17823
C	3.86358	3.59999	-1.86590
H	3.00408	2.93434	-2.05202
H	4.56975	3.47075	-2.70505
H	3.49886	4.64338	-1.89198
C	2.55485	1.42837	4.80451
H	3.65058	1.45632	4.68581
H	2.30104	0.56412	5.44342
H	2.25376	2.34474	5.34373
C	-1.19462	2.74312	-4.66374
H	-2.25391	3.03598	-4.59732
H	-1.11068	1.93947	-5.41715
H	-0.62882	3.61416	-5.04137
C	-3.81476	5.52636	0.88701
H	-3.27592	6.47333	1.07218
H	-4.58727	5.42837	1.66969
H	-4.32420	5.61790	-0.08619
C	5.80164	4.16455	-0.34387
H	5.55563	5.24017	-0.40450
H	6.53246	3.95105	-1.14331
H	6.29389	3.99134	0.62677
Mg	2.14503	-0.33140	0.06523
C	0.55536	-1.35537	1.06349
O	-0.19402	-2.22244	1.40419

B

SCF (BP86) Energy = -2697.05369445

Enthalpy 0K = -2695.616952

Enthalpy 298K = -2695.517392

Free Energy 298K = -2695.753252

Lowest Frequency = 13.6192 cm^{-1}

Second Frequency = 18.1068 cm^{-1}

SCF (BP86-D3BJ) Energy = -2697.56355598
SCF (Benzene) Energy = -2697.06576272
SCF (BS2) Energy = -4562.88187986

```
Si -5.08411  1.27209 -1.47796
Si -5.05656 -1.43434  1.53921
Si  5.01417  1.24206  1.58821
Si  5.02102 -1.28221 -1.54363
Mg  2.28209 -0.02430  0.03074
Na  0.06118  3.22253 -0.15636
Na  0.12972 -2.84879  0.12464
N  -3.42531  1.47986 -0.85828
N  -3.41048 -1.58023  0.88278
N   3.37457 -1.48232 -0.89984
N   3.37247  1.46279  0.94330
C  -2.82688  2.75121 -0.98402
C   2.78620  2.73940  1.04158
C  -1.93077 -3.10906  2.18665
C  -2.92135  3.72520  0.08163
C  -2.72874 -2.80786  1.01979
C  -2.09932  3.13903 -2.17286
C   2.75719 -2.74071 -1.06253
C   2.00834  3.12512  2.19842
C   2.91773  3.71617 -0.01803
C   2.88467 -3.77627 -0.06124
C  -1.48870  4.40978 -2.24675
H  -0.95737  4.69064 -3.16415
C  -1.99744  2.21136 -3.38379
H  -2.45345  1.25324 -3.07844
C   6.32095 -0.74303 -0.24013
H   6.27929 -1.45857  0.60412
H   7.29089 -0.95143 -0.74010
C  -2.78749 -3.80899 -0.02147
C  -2.27670  4.97455 -0.03438
H  -2.36363  5.69676  0.78610
C  -1.22115 -4.32714  2.26409
H  -0.63253 -4.54282  3.16398
C   6.31800  0.71091  0.28403
H   6.27159  1.42703 -0.55948
H   7.28776  0.92343  0.78261
C  -6.34963  0.64980 -0.17589
H  -6.29162  1.31042  0.71094
H  -7.32745  0.89260 -0.64401
C   1.86491  2.18951  3.39883
H   2.38980  1.25737  3.12539
C   1.95163 -3.03958 -2.22473
C  -6.34541 -0.83609  0.25183
H  -6.28650 -1.49627 -0.63518
H  -7.31927 -1.08343  0.72527
C   1.38845  4.39144  2.25112
H   0.81141  4.66389  3.14338
C  -1.56013  5.33146 -1.18882
H  -1.08590  6.31474 -1.27109
C   2.20364 -5.00153 -0.21580
H   2.31442 -5.77197  0.55671
C   3.76176  3.42317 -1.25720
H   4.19557  2.42116 -1.09441
C  -2.04712 -5.00253  0.09790
H  -2.10225 -5.74579 -0.70607
C   1.40556 -5.26724 -1.33918
H   0.89101 -6.22763 -1.44390
C   2.28286  4.97251  0.08505
H   2.41059  5.70062 -0.72556
C  -3.67445 -3.60601 -1.24948
H  -4.28116 -2.70788 -1.03871
C  -1.85770 -2.14432  3.36940
H  -2.40610 -1.23656  3.06218
C   3.76055 -3.57168  1.17472
H   4.29455 -2.61907  1.01337
C   1.29342 -4.28295 -2.33240
H   0.69288 -4.49051 -3.22650
C   1.51435  5.32325  1.20772
H   1.04001  6.30753  1.27601
C   5.07178  0.00090 -2.96284
H   4.55685 -0.37920 -3.86150
H   6.11380  0.23570 -3.24497
H   4.58398  0.94672 -2.67325
C  -1.26341 -5.27513  1.23013
H  -0.70644 -6.21377  1.31046
C  -5.12905  0.02871 -2.93120
H  -4.61106  0.42549 -3.82037
H  -6.16972 -0.20300 -3.22066
H  -4.64174 -0.92294 -2.65675
C  -5.79650  2.94634 -2.06577
H  -6.07437  3.57733 -1.20400
H  -6.71057  2.77822 -2.66159
H  -5.08954  3.52431 -2.68193
C   5.03086 -0.07094  2.97895
H   4.50327  0.29290  3.87712
H   6.06466 -0.32554  3.27349
H   4.53733 -1.00314  2.65555
C  -3.75817  3.43940  1.32821
H  -4.29382  2.49646  1.12125
C   5.69450  2.88263  2.29811
H   5.96737  3.58404  1.49131
H   6.60648  2.68596  2.88846
H   4.97205  3.39913  2.94998
C  -2.78805  2.76154 -4.59386
H  -3.85219  2.91620 -4.35395
H  -2.72945  2.05935 -5.44444
H  -2.37667  3.72981 -4.93204
C   1.82567 -2.04015 -3.37379
H   2.36746 -1.13315 -3.05304
C  -5.12575 -0.20220  3.00238
H  -4.59411 -0.59283  3.88624
H  -6.17054  0.00002  3.29918
H  -4.66368  0.76353  2.73370
C  -0.53475  1.93982 -3.79234
H  -0.01499  2.86669 -4.09485
H  -0.49720  1.25115 -4.65392
H   0.03279  1.48006 -2.96583
C   2.52998  2.76930  4.66850
H   3.59915  2.98287  4.51004
H   2.44709  2.05542  5.50722
H   2.04336  3.71024  4.98311
C  -0.40731 -1.74085  3.70436
H   0.20072 -2.61232  4.00612
H  -0.38812 -1.02374  4.54259
H   0.09072 -1.26112  2.84100
C   0.39193  1.84408  3.69348
H  -0.19629  2.74510  3.94166
H   0.31831  1.15649  4.55327
H  -0.08819  1.35045  2.82995
C  -5.69487 -3.13223  2.14133
H  -5.92813 -3.79306  1.28892
H  -6.62246 -3.00310  2.72583
```

H -4.96375 -3.65882 2.77616
C   0.36101 -1.65213 -3.65822
H -0.24377 -2.52707 -3.95509
H   0.30622 -0.91949 -4.48129
H -0.11522 -1.19271 -2.77275
C   5.68068 -2.94029 -2.23142
H   5.90336 -3.65137 -1.41785
H   6.61803 -2.76856 -2.78900
H   4.96598 -3.43191 -2.91126
C   2.91047 -3.42156 2.45547
H   2.22075 -2.56032 2.38381
H   3.54971 -3.25705 3.34102
H   2.30391 -4.32694 2.64247
C   4.92107 4.42976 -1.42691
H   4.54778 5.45168 -1.62108
H   5.55927 4.14210 -2.28076
H   5.55471 4.47247 -0.52591
C -4.80540 4.54099 1.60149
H -4.33189 5.50025 1.87827
H -5.46160 4.24560 2.43871
H -5.43891 4.72489 0.71803
C   2.90549 3.36994 -2.54135
H   2.11677 2.60034 -2.47268
H   3.52760 3.12613 -3.42062
H   2.41699 4.34162 -2.74153
C -2.87667 3.21207 2.57353
H -2.18480 2.36375 2.42892
H -3.49311 2.98891 3.46231
H -2.26752 4.10568 2.80312
C -2.83985 -3.31831 -2.51527
H -2.21901 -2.41249 -2.39287
H -3.48991 -3.15759 -3.39362
H -2.16037 -4.15876 -2.74709
C -2.54898 -2.72719 4.62370
H -3.60299 -2.98224 4.42726
H -2.52316 -1.99939 5.45419
H -2.04120 -3.64595 4.96874
C   2.49159 -2.56927 -4.66523
H   3.55470 -2.81149 -4.50505
H   2.43149 -1.81437 -5.46935
H   1.99090 -3.48483 -5.02870
C   4.80690 -4.69278 1.35404
H   4.33275 -5.66549 1.57804
H   5.48305 -4.45575 2.19405
H   5.42035 -4.82294 0.44725
C -4.63220 -4.79264 -1.49128
H -4.08731 -5.71175 -1.77282
H -5.32974 -4.56009 -2.31482
H -5.22772 -5.02071 -0.59174
Mg -2.38007 -0.06528 -0.01196
C -0.03574 -0.17128 -0.01504
O   0.51854 0.95910 -0.38069

B + CO

SCF (BP86) Energy = -2810.35657819
Enthalpy 0K = -2808.912536
Enthalpy 298K = -2808.810590
Free Energy 298K = -2809.049027
Lowest Frequency = 14.6258 cm^{-1}
Second Frequency = 18.0775 cm^{-1}
SCF (BP86-D3BJ) Energy = -2810.88376317
SCF (Benzene) Energy = -2810.36755754
SCF (BS2) Energy = -4676.21863641

Si -5.32531 -0.66392 1.31271
Si -4.68541 1.80102 -1.81701
Si 4.79567 -1.33997 -1.78559
Si 5.23447 0.96275 1.46754
Mg 2.39666 -0.14438 0.14885
Na -0.60782 -3.41139 -0.07188
Na 0.23924 2.87164 0.20623
N -3.66900 -1.12818 0.84047
N -3.09546 1.78465 -1.01347
N   3.58897 1.33064 0.90889
N   3.24343 -1.66225 -0.97858
C -3.28487 -2.47907 0.99801
C   2.57524 -2.88997 -1.12764
C -1.43049 3.23905 -2.17030
C -3.47795 -3.44431 -0.06359
C -2.33982 2.97651 -1.07812
C -2.68231 -2.95844 2.22421
C   2.98574 2.56340 1.22520
C   1.61756 -3.10160 -2.19495
C   2.79000 -3.99265 -0.20807
C   3.14970 3.72569 0.37882
C -2.25522 -4.29868 2.32662
H -1.79913 -4.64064 3.26236
C -2.56894 -2.05519 3.45413
H -2.69978 -1.01922 3.09156
C   6.40808 0.41522 0.05262
H   6.37897 1.19267 -0.73530
H   7.42145 0.50145 0.49955
C -2.43542 3.98043 -0.04172
C -3.03455 -4.77707 0.09218
H -3.20958 -5.49421 -0.71947
C -0.68419 4.43693 -2.19923
H -0.01267 4.62388 -3.04559
C   6.25076 -0.99101 -0.57531
H   6.23407 -1.76668 0.21424
H   7.15492 -1.21868 -1.17886
C -6.40593 0.03792 -0.11011
H -6.39318 -0.69395 -0.94149
H -7.43424 -0.02694 0.30597
C   1.40603 -2.05283 -3.28771
H   1.88201 -1.12426 -2.92462
C   2.14742 2.71530 2.39567
C -6.16143 1.46672 -0.64090
H -6.09707 2.18617 0.19859
H -7.04434 1.79641 -1.22895
C   0.91318 -4.32021 -2.28950
H   0.22086 -4.47262 -3.12678
C -2.41115 -5.21354 1.27282
H -2.08189 -6.25231 1.38124
C   2.45483 4.91848 0.66624
H   2.58864 5.78222 0.00421
C   3.88388 -3.92674 0.85867
H   4.10836 -2.85456 1.00367
C -1.65997 5.15614 -0.11370
H -1.75549 5.90510 0.68146
C   1.61331 5.03683 1.78335
H   1.08895 5.97481 1.99152
C   2.04911 -5.18595 -0.34251
H   2.23163 -6.00496 0.36223
C -3.38115 3.79871 1.14442
H -3.95704 2.88151 0.93122
C -1.26058 2.24970 -3.32373

H	-1.85942	1.35984	-3.06040
C	4.08981	3.70002	-0.82570
H	4.60664	2.72539	-0.79023
C	1.47890	3.93425	2.64111
H	0.85568	4.02665	3.53882
C	1.10602	-5.36326	-1.36488
H	0.56339	-6.30932	-1.46867
C	5.23005	-0.43827	2.77307
H	4.77471	-0.10020	3.71955
H	6.26061	-0.76963	2.99452
H	4.66470	-1.32096	2.42842
C	-0.78774	5.40032	-1.18503
H	-0.20397	6.32495	-1.23110
C	-5.29145	0.65544	2.69642
H	-4.89137	0.23745	3.63528
H	-6.30902	1.03471	2.90001
H	-4.66462	1.51957	2.42056
C	-6.33512	-2.17372	1.94361
H	-7.10982	-2.45238	1.20855
H	-6.85852	-1.92239	2.88264
H	-5.71642	-3.06581	2.13040
C	4.63528	0.19036	-2.91929
H	3.95010	-0.00772	-3.76106
H	5.61662	0.47485	-3.34042
H	4.24939	1.06074	-2.36226
C	-4.23544	-3.07790	-1.34004
H	-4.42720	-1.99209	-1.27918
C	5.35920	-2.82339	-2.87255
H	6.32872	-3.22390	-2.52985
H	5.49514	-2.50312	-3.92022
H	4.63355	-3.65307	-2.86520
C	-3.70554	-2.35682	4.46211
H	-4.70253	-2.24516	4.01122
H	-3.64220	-1.67617	5.33002
H	-3.62273	-3.39202	4.84006
C	2.01389	1.59200	3.42247
H	2.54044	0.71932	3.00012
C	-4.75908	0.50892	-3.22536
H	-4.09636	0.79360	-4.06010
H	-5.78496	0.41592	-3.62441
H	-4.44641	-0.49019	-2.87692
C	-1.21713	-2.15694	4.18788
H	-1.06694	-3.15283	4.64206
H	-1.17800	-1.42077	5.00953
H	-0.36614	-1.96518	3.51650
C	2.10707	-2.47087	-4.60261
H	3.18218	-2.65165	-4.45343
H	1.99359	-1.68489	-5.37064
H	1.66357	-3.40003	-5.00452
C	0.21010	1.81630	-3.49629
H	0.86440	2.67653	-3.72058
H	0.31430	1.10494	-4.33272
H	0.59786	1.31825	-2.58804
C	-0.08101	-1.76581	-3.57018
H	-0.61034	-2.66329	-3.93561
H	-0.18740	-0.99626	-4.35275
H	-0.60129	-1.39819	-2.66866
C	-5.05607	3.52449	-2.55755
H	-5.26189	4.26089	-1.76196
H	-5.95252	3.46975	-3.19980
H	-4.22655	3.92168	-3.16374
C	0.54803	1.19094	3.68220
H	-0.03365	2.02074	4.12302
H	0.49556	0.34295	4.38564
H	0.04161	0.88177	2.74861
C	6.04800	2.49101	2.27690
H	6.37859	3.22439	1.52275
H	6.93801	2.18221	2.85245
H	5.36151	3.00867	2.96759
C	3.31808	3.77457	-2.16036
H	2.60699	2.93724	-2.26261
H	4.00987	3.72840	-3.01987
H	2.74508	4.71661	-2.23870
C	5.17559	-4.61185	0.35019
H	4.99975	-5.68902	0.17697
H	5.98476	-4.51665	1.09609
H	5.52824	-4.17476	-0.59585
C	-5.59916	-3.80369	-1.40839
H	-5.46319	-4.89734	-1.49108
H	-6.17191	-3.46957	-2.29150
H	-6.20634	-3.61025	-0.51047
C	3.48991	-4.54275	2.21820
H	2.53705	-4.13916	2.59904
H	4.27429	-4.33474	2.96641
H	3.38834	-5.64150	2.15698
C	-3.42869	-3.35609	-2.62477
H	-2.46948	-2.81193	-2.63264
H	-3.99508	-3.03609	-3.51699
H	-3.20907	-4.43274	-2.74569
C	-2.60565	3.57902	2.46259
H	-1.94991	2.68971	2.40629
H	-3.29744	3.42306	3.30920
H	-1.96934	4.44972	2.70447
C	-1.79598	2.81743	-4.65885
H	-2.86088	3.09099	-4.58974
H	-1.68831	2.07226	-5.46700
H	-1.23701	3.72114	-4.96196
C	2.71078	1.96784	4.75116
H	3.77360	2.21530	4.59350
H	2.65725	1.12983	5.46867
H	2.22972	2.84435	5.22172
C	5.15081	4.82144	-0.76489
H	4.69352	5.82165	-0.87292
H	5.88091	4.70403	-1.58479
H	5.70140	4.81003	0.18983
C	-4.37868	4.96760	1.29429
H	-3.86736	5.91407	1.54665
H	-5.09951	4.75605	2.10337
H	-4.94769	5.13373	0.36463
Mg	-2.32650	0.20714	0.02681
C	0.99818	-2.45111	1.54981
O	1.71943	-1.53807	1.73661
C	0.03498	0.21453	0.10761
O	-0.55468	-0.93184	0.05078

C

SCF (BP86) Energy = -2810.36497287
Enthalpy 0K = -2808.921006
Enthalpy 298K = -2808.818703
Free Energy 298K = -2809.059646
Lowest Frequency = 15.2879 cm^{-1}
Second Frequency = 15.5214 cm^{-1}
SCF (BP86-D3BJ) Energy = -2810.88768071
SCF (Benzene) Energy = -2810.37664354
SCF (BS2) Energy = -4676.22777765

Si	-4.77306	1.66770	-1.96316
Si	-5.43499	-1.02703	1.01388
Si	5.22560	1.02651	1.50383
Si	5.00286	-1.44576	-1.68101
Mg	2.51203	-0.22007	0.15999
Na	0.13868	3.17440	0.34869
Na	-0.29375	-2.94454	-0.17275
N	-3.22467	1.70209	-1.08492
N	-3.68988	-1.27220	0.73042
N	3.41193	-1.69642	-0.93343
N	3.58085	1.35573	0.90117
C	-2.54258	2.93370	-1.01907
C	2.99043	2.60358	1.17887
C	-2.70061	-2.91914	2.33410
C	-2.69316	3.81174	0.12023
C	-3.16577	-2.56003	1.01672
C	-1.65764	3.36658	-2.07735
C	2.66361	-2.87294	-1.13606
C	2.20950	2.82847	2.37771
C	3.14514	3.72199	0.27326
C	2.65912	-3.93670	-0.15112
C	-0.98412	4.60282	-1.97761
H	-0.33001	4.91920	-2.79920
C	-1.44084	2.51115	-3.32646
H	-2.02820	1.58928	-3.17320
C	6.36131	-1.00821	-0.39659
H	6.29422	-1.73445	0.43613
H	7.30964	-1.25646	-0.91875
C	-3.08352	-3.56338	-0.01883
C	-1.99787	5.03879	0.17144
H	-2.14506	5.69813	1.03565
C	-2.07488	-4.16432	2.53951
H	-1.70689	-4.41391	3.54159
C	6.44850	0.43812	0.14735
H	6.42164	1.16695	-0.68574
H	7.44148	0.58847	0.62269
C	-6.26776	1.14218	-0.88845
H	-6.30060	1.79397	0.00612
H	-7.14567	1.44861	-1.49613
C	2.09070	1.75530	3.45986
H	2.55356	0.84187	3.04601
C	1.84381	-3.05766	-2.31349
C	-6.40938	-0.34268	-0.49006
H	-6.22149	-1.00118	-1.36055
H	-7.46328	-0.54872	-0.20519
C	1.59008	4.07842	2.59891
H	1.01800	4.23111	3.52221
C	-1.14606	5.44873	-0.86799
H	-0.62804	6.41173	-0.81930
C	1.84935	-5.07536	-0.34217
H	1.86277	-5.86937	0.41251
C	4.04996	3.61279	-0.95333
H	4.58506	2.65306	-0.84891
C	-2.44076	-4.79447	0.23703
H	-2.38354	-5.54496	-0.56082
C	1.05005	-5.23423	-1.48642
H	0.44644	-6.13736	-1.62411
C	2.49280	4.94523	0.53291
H	2.62284	5.77650	-0.17020
C	-3.73214	-3.33315	-1.38619
H	-4.34978	-2.42430	-1.27903
C	-2.93027	-2.00047	3.53716
H	-3.29202	-1.04043	3.12903
C	3.59444	-3.88517	1.05766
H	3.82474	-2.81705	1.22701
C	1.06599	-4.22607	-2.46276
H	0.47240	-4.35519	-3.37598
C	1.71199	5.13844	1.68498
H	1.23051	6.10230	1.87919
C	4.97359	-0.00616	-2.94363
H	4.40736	-0.27137	-3.85232
H	6.00083	0.25829	-3.25420
H	4.51452	0.89906	-2.51054
C	-1.91308	-5.09529	1.50333
H	-1.41978	-6.05536	1.68744
C	-4.69523	0.45367	-3.43953
H	-4.02263	0.82100	-4.23278
H	-5.69543	0.30746	-3.88491
H	-4.32619	-0.53695	-3.12088
C	-5.24305	3.41043	-2.59417
H	-5.59866	4.04207	-1.76178
H	-6.06641	3.33357	-3.32563
H	-4.40853	3.94407	-3.07486
C	5.21596	-0.32215	2.86261
H	4.78402	0.05718	3.80386
H	6.24301	-0.66846	3.07687
H	4.62348	-1.20280	2.55913
C	-3.63106	3.44983	1.27077
H	-4.09204	2.48596	0.99061
C	6.00736	2.60977	2.23620
H	6.33871	3.29726	1.43965
H	6.89511	2.34572	2.83695
H	5.31089	3.16466	2.88583
C	-1.94461	3.20485	-4.61305
H	-3.01628	3.45200	-4.55577
H	-1.79503	2.54695	-5.48741
H	-1.39335	4.14290	-4.80586
C	1.83233	-2.02647	-3.44175
H	2.41014	-1.16053	-3.07516
C	-5.78033	0.17212	2.46642
H	-5.49865	-0.26874	3.43679
H	-6.85457	0.42663	2.51029
H	-5.22029	1.11623	2.35296
C	0.03923	2.11566	-3.50150
H	0.68107	3.00263	-3.64103
H	0.16839	1.47387	-4.38931
H	0.41706	1.55246	-2.62919
C	2.87511	2.15658	4.73125
H	3.93655	2.35520	4.51136
H	2.82696	1.35122	5.48550
H	2.45175	3.06941	5.18827
C	-1.66295	-1.71810	4.37049
H	-1.24682	-2.64105	4.81288
H	-1.90637	-1.03790	5.20591
H	-0.87166	-1.25151	3.76743
C	0.62795	1.42963	3.82614
H	0.11120	2.30049	4.26906
H	0.58864	0.61626	4.57094
H	0.05151	1.10617	2.94262
C	-6.29606	-2.69880	1.37011
H	-6.48319	-3.24076	0.42693
H	-7.27478	-2.52476	1.85049
H	-5.70867	-3.36442	2.02144
C	0.41157	-1.53835	-3.78814
H	-0.08004	-1.07363	-2.91457
H	-0.23242	-2.36192	-4.14419

```
H   0.44937 -0.78476 -4.59344
C   5.61139 -3.02198 -2.59485
H   6.43978 -3.50659 -2.05034
H   5.98985 -2.76294 -3.59898
H   4.81094 -3.77006 -2.71737
C   3.00493 -4.47918  2.35389
H   2.02641 -4.04366  2.61072
H   3.69188 -4.28988  3.19684
H   2.88139 -5.57519  2.28437
C   5.09185  4.75056 -1.02291
H   4.61883  5.73154 -1.21061
H   5.80214  4.56737 -1.84795
H   5.66757  4.83243 -0.08628
C  -4.76435  4.48526  1.44342
H  -4.37036  5.47344  1.74235
H  -5.47038  4.15782  2.22657
H  -5.33090  4.62105  0.50743
C   3.23807  3.54818 -2.26315
H   2.55674  2.68082 -2.26847
H   3.90432  3.45244 -3.13862
H   2.62847  4.46038 -2.40204
C  -2.87225  3.25168  2.60175
H  -2.10098  2.46471  2.52153
H  -3.56487  2.95224  3.40790
H  -2.37211  4.18326  2.92476
C  -2.68980 -3.04882 -2.48695
H  -2.08607 -2.15099 -2.25299
H  -3.17290 -2.86643 -3.46346
H  -1.99537 -3.90035 -2.61422
C  -4.02686 -2.57908  4.46491
H  -4.97778 -2.74296  3.93415
H  -4.22127 -1.89259  5.30858
H  -3.70971 -3.54860  4.88981
C   2.52701 -2.57068 -4.71194
H   3.56042 -2.89129 -4.50659
H   2.55776 -1.79650 -5.49942
H   1.98134 -3.44055 -5.12049
C   4.92935 -4.59766  0.73062
H   4.75708 -5.67241  0.53962
H   5.63357 -4.51126  1.57715
H   5.41038 -4.17129 -0.16226
C  -4.65198 -4.49925 -1.80800
H  -4.08056 -5.42232 -2.01383
H  -5.19924 -4.24072 -2.73147
H  -5.39067 -4.73264 -1.02368
Mg -2.43677  0.10494 -0.08828
C  -0.03420 -0.20742 -0.00125
O  -0.54817  0.89174  0.41034
C   1.47713 -1.37474  1.78517
O   0.51906 -2.03795  1.97949
```

TS(C-10)

SCF (BP86) Energy = -2810.36229814

Enthalpy 0K = -2808.918788

Enthalpy 298K = -2808.817032

Free Energy 298K = -2809.058232

Lowest Frequency = -181.1697 cm^{-1}

Second Frequency = 12.4625 cm^{-1}

SCF (BP86-D3BJ) Energy = -2810.88190458

SCF (Benzene) Energy = -2810.37484898

SCF (BS2) Energy = -4676.22512586

```
Si -4.86252  1.73033 -1.89476
Si -5.51633 -1.00727  1.04513
Si  5.37900  1.12355  1.43230
Si  5.15116 -1.49390 -1.70156
Mg  2.74515 -0.25564  0.19997
Na  0.11481  3.11802  0.35936
Na -0.42578 -2.98476 -0.17507
N  -3.29802  1.72213 -1.04583
N  -3.78475 -1.27973  0.71896
N   3.56519 -1.70284 -0.93866
N   3.69899  1.37601  0.89224
C  -2.58585  2.93608 -0.98583
C   3.05275  2.59769  1.16753
C  -2.79024 -3.00118  2.24002
C  -2.69707  3.81234  0.15925
C  -3.27471 -2.58344  0.94548
C  -1.70754  3.35189 -2.05621
C   2.78472 -2.87131 -1.06361
C   2.26920  2.79113  2.36782
C   3.15396  3.71207  0.25156
C   2.77070 -3.87256 -0.01932
C  -1.00452  4.57193 -1.96382
H  -0.35653  4.87568 -2.79512
C  -1.52937  2.49258 -3.30842
H  -2.15411  1.59575 -3.15537
C   6.49716 -0.94812 -0.44445
H   6.45890 -1.64007  0.41909
H   7.44825 -1.19282 -0.96336
C  -3.21496 -3.54326 -0.13270
C  -1.97542  5.02439  0.20188
H  -2.09423  5.68413  1.07018
C  -2.18994 -4.26720  2.38704
H  -1.80936 -4.56177  3.37184
C   6.55263  0.52445  0.03556
H   6.45876  1.21591 -0.82408
H   7.56083  0.73038  0.45439
C  -6.34468  1.22094 -0.79523
H  -6.34188  1.85646  0.11156
H  -7.22962  1.55745 -1.37617
C   2.18615  1.71093  3.44635
H   2.65435  0.80545  3.02055
C   1.93862 -3.09482 -2.21333
C  -6.50966 -0.26800 -0.42071
H  -6.35751 -0.91395 -1.30755
H  -7.56100 -0.45614 -0.11452
C   1.61407  4.02201  2.59451
H   1.04404  4.15659  3.52172
C  -1.13183  5.41893 -0.85038
H  -0.59360  6.37112 -0.80820
C   1.93396 -5.00134 -0.13424
H   1.94478 -5.75359  0.66274
C   4.02038  3.60800 -1.00300
H   4.64216  2.70626 -0.86827
C  -2.60073 -4.79918  0.06706
H  -2.56704 -5.51644 -0.76248
C   1.11224 -5.20457 -1.25509
H   0.49111 -6.10283 -1.33609
C   2.46929  4.91548  0.51825
H   2.56149  5.74866 -0.18803
C  -3.84764 -3.23819 -1.49249
H  -4.40786 -2.29578 -1.36068
C  -2.96375 -2.11821  3.47898
H  -3.26187 -1.12124  3.10858
C   3.71982 -3.77727  1.17575
```

```
H   4.08464 -2.73418  1.19715
C   1.13004 -4.24947 -2.28549
H   0.51795 -4.41505 -3.18065
C   1.69948  5.08595  1.68155
H   1.19409  6.03612  1.88234
C   5.11698 -0.15124 -3.06621
H   4.55245 -0.48257 -3.95386
H   6.14286  0.09657 -3.39402
H   4.65136  0.77994 -2.69908
C  -2.06885 -5.16363  1.31457
H  -1.59853 -6.14237  1.45596
C  -4.84133  0.53582 -3.38932
H  -4.18240  0.90385 -4.19374
H  -5.85453  0.41310 -3.81186
H  -4.48161 -0.46560 -3.09478
C  -5.30081  3.48994 -2.49969
H  -5.58384  4.13613 -1.65081
H  -6.16530  3.44516 -3.18486
H  -4.47302  3.98867 -3.02774
C   5.46642 -0.18868  2.82243
H   5.01287  0.18151  3.75684
H   6.51460 -0.46288  3.03880
H   4.93480 -1.11507  2.54134
C  -3.61459  3.45942  1.32893
H  -4.11658  2.51818  1.04248
C   6.12245  2.75568  2.08694
H   6.32681  3.46263  1.26527
H   7.07894  2.55430  2.59972
H   5.45353  3.26103  2.80290
C  -2.00777  3.20966 -4.59143
H  -3.06664  3.50640 -4.52656
H  -1.89480  2.54607 -5.46700
H  -1.41526  4.12134 -4.78845
C   1.92941 -2.11854 -3.38869
H   2.51471 -1.24073 -3.06394
C  -5.79672  0.17143  2.52748
H  -5.48339 -0.28599  3.48015
H  -6.86517  0.43862  2.61508
H  -5.22930  1.11023  2.40509
C  -0.06791  2.03266 -3.48563
H   0.61178  2.89051 -3.62957
H   0.03036  1.38184 -4.37110
H   0.28592  1.45726 -2.61154
C   2.99083  2.12091  4.70228
H   4.04296  2.34253  4.45937
H   2.97560  1.31167  5.45365
H   2.55972  3.02343  5.17226
C  -1.68562 -1.95636  4.32949
H  -1.36539 -2.91618  4.77402
H  -1.88000 -1.26322  5.16713
H  -0.84438 -1.56455  3.74096
C   0.73524  1.36108  3.83880
H   0.22001  2.21562  4.31399
H   0.72212  0.52900  4.56356
H   0.14154  1.05097  2.96219
C  -6.41026 -2.66806  1.37708
H  -6.68138 -3.14990  0.42158
H  -7.34739 -2.49140  1.93348
H  -5.80369 -3.38784  1.94815
C   0.51043 -1.63745 -3.75188
H  -0.13350 -2.46845 -4.09094
H   0.55233 -0.90359 -4.57528
H   0.01725 -1.15151 -2.89119
C   5.77602 -3.13293 -2.47377
H   6.48934 -3.64211 -1.80344
H   6.30161 -2.93825 -3.42468
H   4.95214 -3.83622 -2.67883
C   3.06317 -4.09698  2.53502
H   2.17937 -3.46832  2.72535
H   3.78683 -3.92583  3.35117
H   2.75060 -5.15499  2.60169
C   4.95336  4.82199 -1.19632
H   4.39164  5.74374 -1.43294
H   5.64580  4.63961 -2.03640
H   5.55456  5.02032 -0.29341
C  -4.70455  4.52865  1.56102
H  -4.26862  5.49526  1.87219
H  -5.39819  4.20584  2.35701
H  -5.29350  4.70716  0.64622
C   3.15956  3.38224 -2.26360
H   2.55318  2.46414 -2.17532
H   3.79045  3.27776 -3.16400
H   2.46992  4.23011 -2.43143
C  -2.82115  3.20283  2.62986
H  -2.08295  2.38988  2.50633
H  -3.49737  2.91000  3.45221
H  -2.27674  4.10843  2.95567
C  -2.78695 -2.99484 -2.58705
H  -2.12791 -2.14265 -2.33278
H  -3.25735 -2.76108 -3.55874
H  -2.14842 -3.88626 -2.73437
C  -4.09914 -2.66063  4.38234
H  -5.05937 -2.73679  3.84924
H  -4.24557 -2.00092  5.25657
H  -3.84667 -3.66723  4.76231
C   2.61766 -2.72560 -4.63358
H   3.65321 -3.03382 -4.41842
H   2.64318 -1.99278 -5.45993
H   2.07128 -3.61626 -4.99348
C   4.94792 -4.69334  0.95668
H   4.64149 -5.75445  0.91911
H   5.67171 -4.57935  1.78321
H   5.46329 -4.46054  0.01174
C  -4.83931 -4.33420 -1.93897
H  -4.32735 -5.28921 -2.15561
H  -5.36312 -4.02622 -2.86091
H  -5.59621 -4.53074 -1.16203
Mg -2.51999  0.09458 -0.09281
C  -0.18180 -0.26635 -0.04401
O  -0.59390  0.85915  0.40359
C   1.26087 -1.19583  1.40073
O   0.36166 -1.87378  1.80857
```

10

SCF (BP86) Energy = -2810.46560206
Enthalpy 0K = -2809.020122
Enthalpy 298K = -2808.917567
Free Energy 298K = -2809.167352
Lowest Frequency = 2.5114 cm^{-1}
Second Frequency = 9.3492 cm^{-1}
SCF (BP86-D3BJ) Energy = -2810.96988650
SCF (Benzene) Energy = -2810.48018996
SCF (BS2) Energy = -4676.33270634

```
Si -5.28587  2.99515 -1.00931
Si -6.55722 -0.73851  1.00543
```

Mg	-3.60518	0.36955	-0.03691
Mg	3.47619	-0.34631	0.02747
Si	5.24915	-2.85057	-1.09454
Na	1.27538	2.80413	0.04513
Na	-1.30126	-2.76689	-0.04627
Si	6.54410	0.63460	0.89220
O	1.78057	0.58077	0.03481
O	-1.90167	-0.55719	-0.13193
N	-4.89058	-1.07944	0.52278
N	-3.71461	2.30817	-0.54742
N	3.68291	-2.25258	-0.50776
N	4.87067	1.01980	0.46305
C	0.55163	0.09971	-0.02573
C	-2.26552	3.65706	0.95359
C	-2.60734	3.17136	-0.36162
C	-0.67037	-0.08450	-0.08227
C	2.26178	-3.66936	0.94378
C	-4.39833	-2.39504	0.38137
C	2.58916	-3.13345	-0.35472
C	3.30280	4.99890	0.22361
H	2.91795	6.02220	0.15998
C	1.75418	-3.50991	-1.46596
C	3.88209	3.02364	1.56954
C	4.38775	2.34297	0.40237
C	3.35853	4.32924	1.45734
H	2.99707	4.83733	2.36014
C	-4.37059	-3.04174	-0.91011
C	4.31566	3.03968	-0.86069
C	-1.77285	3.58090	-1.46381
C	1.18436	-4.56272	1.09015
H	0.96751	-4.98287	2.07930
C	3.77185	4.33852	-0.92435
H	3.73635	4.85530	-1.89108
C	3.07206	-3.25614	2.17065
H	4.01684	-2.83625	1.78018
C	0.69985	-4.42768	-1.27623
H	0.11005	-4.74777	-2.14558
C	1.97108	-2.91551	-2.85600
H	2.83451	-2.23425	-2.76710
C	-3.83477	-3.11511	1.49890
C	-3.04367	3.18779	2.18154
H	-3.98869	2.76245	1.79882
C	0.41323	-4.97226	-0.01159
H	-0.37789	-5.71998	0.10839
C	0.75262	-2.07382	-3.29992
H	0.94931	-1.57910	-4.26768
H	0.51345	-1.29231	-2.55647
H	-0.14477	-2.70676	-3.43539
C	-6.57648	1.60915	-0.74775
H	-7.52861	1.97994	-1.17105
H	-6.29451	0.76326	-1.41045
C	-1.18582	4.54486	1.12387
H	-0.96263	4.92897	2.12619
C	-3.82274	-4.33363	-1.04538
H	-3.81996	-4.81011	-2.03302
C	-6.78208	1.13665	0.72225
H	-7.79173	1.41101	1.07997
H	-6.09036	1.65756	1.41734
C	-1.98087	3.01792	-2.86892
H	-2.95177	2.49612	-2.85201
C	-3.30913	-4.41151	1.31385
H	-2.90117	-4.94857	2.17871
C	-0.71112	4.48354	-1.24625
H	-0.11944	4.82477	-2.10526
C	3.87343	2.34098	2.93622
H	4.34544	1.35485	2.78937
C	-3.73262	-2.47117	2.88152
H	-4.34004	-1.55249	2.83952
C	5.29182	-4.75636	-1.00833
H	5.24799	-5.10812	0.03672
H	6.22168	-5.14542	-1.45783
H	4.44026	-5.20753	-1.54445
C	-5.73427	4.51697	0.05353
H	-5.91999	4.24196	1.10524
H	-6.64321	5.00943	-0.33397
H	-4.91868	5.26034	0.03867
C	-4.88787	-2.32715	-2.15846
H	-5.42359	-1.43141	-1.79742
C	-0.41971	4.98966	0.03318
H	0.37258	5.73295	0.17312
C	-2.27438	-2.04749	3.17953
H	-1.60480	-2.92805	3.21753
H	-2.19905	-1.53726	4.15618
H	-1.89626	-1.35202	2.40931
C	-7.82610	-1.72202	-0.02967
H	-7.82045	-1.41274	-1.08818
H	-8.84812	-1.57578	0.36168
H	-7.60710	-2.80339	0.00671
C	-6.98500	-1.08416	2.83862
H	-6.88442	-2.15144	3.09766
H	-8.03492	-0.79767	3.03068
H	-6.34917	-0.50599	3.53090
C	-3.30424	-5.03452	0.05488
H	-2.91540	-6.05135	-0.06441
C	-5.45937	3.54047	-2.83471
H	-4.79436	4.38204	-3.08789
H	-6.49672	3.87556	-3.01637
H	-5.24587	2.71652	-3.53705
C	4.79952	2.37091	-2.14649
H	5.33161	1.45606	-1.82961
C	3.42448	-4.42787	3.10894
H	4.09933	-4.08458	3.91240
H	3.92921	-5.24243	2.56299
H	2.53020	-4.85410	3.59850
C	2.34599	-2.13424	2.95107
H	1.39710	-2.50515	3.37929
H	2.08368	-1.27702	2.30272
H	2.96803	-1.75793	3.78258
C	2.43104	2.09702	3.43689
H	2.43371	1.56659	4.40553
H	1.86265	1.47890	2.71915
H	1.88740	3.04870	3.58492
C	-2.27694	2.05663	2.90762
H	-1.33199	2.43705	3.33650
H	-2.00327	1.23294	2.22243
H	-2.87593	1.63458	3.73412
C	-0.89109	1.97074	-3.20049
H	0.10357	2.45110	-3.27269
H	-1.09183	1.48853	-4.17392
H	-0.82939	1.18538	-2.42718
C	-3.40031	4.31891	3.16696
H	-4.04454	3.93122	3.97549
H	-3.93993	5.13682	2.66185
H	-2.50390	4.74995	3.64821
C	3.60533	1.94011	-3.03069
H	2.89954	1.29385	-2.47696

```
H   3.94767  1.38426 -3.92144
H   3.03833  2.82078 -3.38569
C   5.63308 -2.30567 -2.89037
H   4.98824 -2.81917 -3.62240
H   6.68297 -2.53140 -3.15020
H   5.48690 -1.21880 -3.02179
C   2.30719 -3.98974 -3.91337
H   1.46738 -4.69402 -4.05598
H   3.19151 -4.58244 -3.62492
H   2.51367 -3.52094 -4.89193
C  -4.27512 -3.37007  4.01207
H  -5.30462 -3.70636  3.80545
H  -4.28052 -2.82182  4.97027
H  -3.65262 -4.27175  4.15614
C  -5.88059 -3.17761 -2.97822
H  -5.39849 -4.07325 -3.40970
H  -6.28506 -2.58858 -3.82005
H  -6.72766 -3.51514 -2.35905
C  -3.71539 -1.85052 -3.04906
H  -2.99888 -1.22560 -2.48477
H  -4.08223 -1.26145 -3.90833
H  -3.15265 -2.71240 -3.45327
C  -2.02210  4.10432 -3.96472
H  -2.75407  4.89596 -3.73443
H  -2.29558  3.65831 -4.93683
H  -1.03788  4.58965 -4.09705
C   6.71763 -2.21915 -0.02632
H   6.49947 -2.42994  1.03877
H   7.52805 -2.93049 -0.29085
C   4.69632  3.12027  3.98474
H   4.25871  4.11409  4.18966
H   5.73390  3.27828  3.64551
H   4.72849  2.56955  4.94143
C   6.74613  0.03441  2.70150
H   6.58434  0.84957  3.42653
H   7.76429 -0.36181  2.86634
H   6.03378 -0.77399  2.94371
C   7.24242 -0.76796 -0.21261
H   7.16763 -0.45394 -1.27166
H   8.32947 -0.74487  0.01180
C   5.78418  3.24663 -2.94920
H   5.30077  4.16021 -3.33997
H   6.17597  2.68767 -3.81694
H   6.64014  3.56075 -2.32888
C   7.65623  2.16646  0.65239
H   7.26442  3.03603  1.20703
H   7.72191  2.45176 -0.41134
H   8.68020  1.96864  1.01350
```

TS(B-D)

SCF (BP86) Energy = -2810.34915000
Enthalpy 0K = -2808.905831
Enthalpy 298K = -2808.804313
Free Energy 298K = -2809.044658
Lowest Frequency = -53.4464 cm^{-1}
Second Frequency = 14.5434 cm^{-1}
SCF (BP86-D3BJ) Energy = -2810.86527898
SCF (Benzene) Energy = -2810.36323225
SCF (BS2) Energy = -4676.21293140

```
Si -5.36389 -0.82458  1.39212
Si -4.96238  1.90727 -1.59953
Si  4.97083 -1.54940 -1.72603
Si  5.43975  0.86096  1.46512
Mg  2.53169 -0.09446  0.03737
Na -0.57351 -4.09783 -0.01815
Na  0.51918  3.51756 -0.01243
N  -3.71773 -1.24185  0.81714
N  -3.32401  1.86285 -0.90012
N   3.80809  1.26314  0.89534
N   3.36733 -1.70709 -0.97696
C  -3.35264 -2.60592  0.94712
C   2.67859 -2.92426 -1.12949
C  -1.79792  3.42607 -2.11279
C  -3.58246 -3.53840 -0.13095
C  -2.57418  3.06389 -0.95091
C  -2.76818 -3.12432  2.16236
C   3.28176  2.54045  1.16439
C   1.81938 -3.16563 -2.26692
C   2.77504 -3.98090 -0.14635
C   3.47013  3.63991  0.24386
C  -2.43927 -4.49276  2.25948
H  -2.01578 -4.87022  3.19777
C  -2.52899 -2.23168  3.38242
H  -2.70859 -1.19489  3.04614
C   6.59952  0.21109  0.08180
H   6.60264  0.95086 -0.74231
H   7.61227  0.28360  0.53257
C  -2.57179  3.98271  0.16203
C  -3.22748 -4.89805  0.01120
H  -3.44051 -5.59341 -0.81050
C  -1.10606  4.65540 -2.14628
H  -0.54905  4.92623 -3.05146
C   6.39283 -1.21781 -0.47657
H   6.29738 -1.94687  0.35117
H   7.30621 -1.52904 -1.02666
C  -6.50596 -0.04716  0.05969
H  -6.49231 -0.70281 -0.83272
H  -7.51701 -0.19119  0.49702
C   1.62935 -2.09914 -3.34503
H   2.23565 -1.23343 -3.02588
C   2.50694  2.80341  2.35568
C  -6.33538  1.43346 -0.34874
H  -6.22227  2.07522  0.54649
H  -7.26802  1.78763 -0.83665
C   1.15330 -4.40085 -2.41492
H   0.54391 -4.57184 -3.31176
C  -2.66103 -5.39091  1.20106
H  -2.43142 -6.45661  1.31303
C   2.89912  4.90189  0.51164
H   3.07238  5.72577 -0.19205
C   3.64251 -3.81612  1.09982
H   3.99186 -2.76835  1.08357
C  -1.84508  5.18965  0.09008
H  -1.87576  5.88138  0.94070
C   2.13189  5.13613  1.66724
H   1.71366  6.12796  1.86976
C   2.09380 -5.20057 -0.33775
H   2.22680 -6.00309  0.39940
C  -3.37121  3.68346  1.42866
H  -3.93374  2.75743  1.21477
C  -1.70122  2.50730 -3.33126
H  -2.24471  1.58313 -3.06615
C   4.26788  3.44825 -1.04458
H   4.74140  2.45454 -0.96047
C   1.94518  4.07833  2.57358
```

H	1.36709	4.25479	3.48916
C	1.29154	-5.43561	-1.47154
H	0.82395	-6.41386	-1.63675
C	5.39997	-0.47515	2.83717
H	4.96521	-0.07702	3.76981
H	6.41894	-0.83513	3.06730
H	4.79904	-1.35053	2.53593
C	-1.12129	5.54679	-1.06036
H	-0.59719	6.50696	-1.11633
C	-5.29348	0.39956	2.85912
H	-4.84387	-0.06454	3.75230
H	-6.30839	0.74267	3.12862
H	-4.69811	1.29554	2.61326
C	-6.31563	-2.39334	1.93155
H	-6.69106	-2.94122	1.05030
H	-7.19266	-2.10182	2.53573
H	-5.71160	-3.09784	2.52362
C	4.99059	-0.12285	-3.00144
H	4.37068	-0.36677	-3.88102
H	6.01736	0.07866	-3.35623
H	4.60126	0.81251	-2.56469
C	-4.24689	-3.08945	-1.43233
H	-4.56371	-2.04508	-1.26467
C	5.47251	-3.15368	-2.63931
H	5.70726	-3.97006	-1.93571
H	6.37478	-2.96943	-3.24850
H	4.67855	-3.52023	-3.30980
C	-3.52435	-2.54960	4.52442
H	-4.57303	-2.43477	4.20945
H	-3.35089	-1.87736	5.38357
H	-3.39377	-3.58706	4.88195
C	2.30882	1.72097	3.41583
H	2.80895	0.81727	3.02618
C	-5.11154	0.70502	-3.08076
H	-4.50404	1.04425	-3.93634
H	-6.15931	0.62177	-3.42065
H	-4.77010	-0.31020	-2.81185
C	-1.08795	-2.32534	3.92962
H	-0.87120	-3.32803	4.34059
H	-0.94660	-1.60171	4.75139
H	-0.33909	-2.11080	3.15190
C	2.13125	-2.55770	-4.73269
H	3.19610	-2.84032	-4.70919
H	2.01170	-1.74725	-5.47364
H	1.56153	-3.42999	-5.10165
C	-0.23681	2.13391	-3.64889
H	0.35817	3.02242	-3.92713
H	-0.19110	1.43227	-4.49998
H	0.24938	1.65751	-2.77991
C	0.15790	-1.64669	-3.44008
H	-0.49716	-2.47883	-3.75466
H	0.04239	-0.83885	-4.18275
H	-0.20687	-1.26781	-2.46870
C	-5.41675	3.67112	-2.17332
H	-5.57770	4.33519	-1.30678
H	-6.35567	3.64622	-2.75344
H	-4.63951	4.13520	-2.80063
C	0.82110	1.38003	3.64177
H	0.25825	2.24784	4.03069
H	0.71693	0.56198	4.37615
H	0.34107	1.05340	2.70338
C	6.30224	2.40481	2.19117
H	6.54549	3.14116	1.40652
H	7.24737	2.11619	2.68328
H	5.67243	2.91494	2.93905
C	3.33244	3.41671	-2.27471
H	2.56873	2.62349	-2.18079
H	3.89879	3.22347	-3.20298
H	2.80821	4.38311	-2.40035
C	4.88721	-4.73198	1.05883
H	4.59873	-5.79901	1.05235
H	5.52318	-4.56233	1.94574
H	5.49916	-4.54615	0.16138
C	-5.50085	-3.92027	-1.78108
H	-5.24693	-4.97030	-2.01337
H	-6.00361	-3.50010	-2.66957
H	-6.22516	-3.92875	-0.95011
C	2.85565	-4.04910	2.40825
H	1.97480	-3.38959	2.46970
H	3.49622	-3.84462	3.28438
H	2.51069	-5.09619	2.49575
C	-3.25118	-3.09613	-2.61164
H	-2.37600	-2.45549	-2.40693
H	-3.72649	-2.72384	-3.53636
H	-2.88050	-4.11765	-2.81837
C	-2.44791	3.41589	2.63767
H	-1.74166	2.59039	2.43814
H	-3.03589	3.14579	3.53278
H	-1.84875	4.30985	2.89009
C	-2.36948	3.11927	-4.58411
H	-3.43452	3.34525	-4.41511
H	-2.30131	2.42135	-5.43753
H	-1.87153	4.05909	-4.88374
C	2.98493	2.10343	4.75218
H	4.05985	2.30948	4.61730
H	2.88352	1.28499	5.48706
H	2.52494	3.00633	5.19332
C	5.37672	4.50415	-1.23564
H	4.96103	5.51739	-1.38479
H	5.98454	4.26595	-2.12605
H	6.04985	4.54574	-0.36327
C	-4.39361	4.79168	1.76176
H	-3.89602	5.74611	2.01131
H	-5.00636	4.50118	2.63313
H	-5.07308	4.98154	0.91429
Mg	-2.57326	0.20275	-0.02408
C	0.00799	-1.81444	0.65254
O	1.09910	-1.41867	0.95164
C	0.48415	1.00956	-0.15222
O	-0.58963	0.35657	-0.03970

D

SCF (BP86) Energy = -2810.36095510
Enthalpy 0K = -2808.917522
Enthalpy 298K = -2808.815248
Free Energy 298K = -2809.056537
Lowest Frequency = 17.4575 cm^{-1}
Second Frequency = 18.1821 cm^{-1}
SCF (BP86-D3BJ) Energy = -2810.87962153
SCF (Benzene) Energy = -2810.37417782
SCF (BS2) Energy = -4676.22522483

Si	5.30654	-0.80348	-1.53895
Si	4.93009	1.67237	1.63629
Si	-4.93160	-1.67125	1.63748
Si	-5.30740	0.80239	-1.53997

Mg	-2.44568	-0.09637	0.03916
Na	0.47654	-3.84129	0.01142
Na	-0.47446	3.84095	0.01263
N	3.69489	-1.23522	-0.91704
N	3.27862	1.73191	0.97111
N	-3.69573	1.23425	-0.91829
N	-3.28014	-1.73121	0.97247
C	3.26347	-2.56449	-1.11757
C	-2.59054	-2.95664	1.09896
C	1.78931	3.26170	2.26185
C	3.52159	-3.59247	-0.13447
C	2.58986	2.95785	1.09712
C	2.53986	-2.95565	-2.30524
C	-3.26296	2.56289	-1.11978
C	-1.78919	-3.25898	2.26347
C	-2.65493	-3.96612	0.06628
C	-3.52113	3.59221	-0.13809
C	2.08642	-4.28246	-2.45882
H	1.54893	-4.55788	-3.37462
C	2.26550	-1.95643	-3.42922
H	2.64679	-0.98296	-3.07369
C	-6.49839	0.11468	-0.20239
H	-6.53232	0.84126	0.63270
H	-7.49702	0.18957	-0.68312
C	2.65470	3.96667	0.06377
C	3.05297	-4.90749	-0.33424
H	3.27808	-5.67368	0.41847
C	1.14698	4.51263	2.37882
H	0.57497	4.73187	3.28896
C	-6.30001	-1.32050	0.33697
H	-6.16804	-2.03466	-0.49905
H	-7.23015	-1.65064	0.84664
C	6.49740	-0.11472	-0.20184
H	6.53130	-0.84073	0.63377
H	7.49606	-0.18995	-0.68248
C	-1.63301	-2.25133	3.40357
H	-2.14346	-1.32982	3.07103
C	-2.53740	2.95189	-2.30696
C	6.29881	1.32078	0.33638
H	6.16695	2.03430	-0.50020
H	7.22875	1.65142	0.84607
C	-1.14547	-4.50915	2.38090
H	-0.57277	-4.72722	3.29089
C	2.33151	-5.26909	-1.48749
H	2.00396	-6.30305	-1.64304
C	-3.05075	4.90645	-0.33874
H	-3.27590	5.67375	0.41283
C	-3.45987	-3.73206	-1.21055
H	-3.92476	-2.73755	-1.09247
C	1.99103	5.20175	0.22354
H	2.09142	5.96436	-0.55937
C	-2.32721	5.26589	-1.49138
H	-1.99822	6.29930	-1.64760
C	-1.99001	-5.20046	0.22650
H	-2.09013	-5.96370	-0.55582
C	3.45895	3.73111	-1.21322
H	3.92333	2.73644	-1.09452
C	1.63262	2.25495	3.40270
H	2.14063	1.33217	3.06988
C	-4.28756	3.27670	1.14492
H	-4.65181	2.24066	1.03117
C	-2.08201	4.27794	-2.46132
H	-1.54300	4.55174	-3.37672
C	-1.24443	-5.49498	1.38311
H	-0.77495	-6.47639	1.51424
C	-5.19630	-0.50400	-2.93454
H	-4.74227	-0.07625	-3.84450
H	-6.20001	-0.87966	-3.20283
H	-4.58621	-1.37257	-2.63369
C	1.24638	5.49775	1.38034
H	0.77783	6.47965	1.51106
C	5.19540	0.50186	-2.93449
H	4.74172	0.07338	-3.84427
H	6.19909	0.87759	-3.20276
H	4.58500	1.37049	-2.63439
C	6.20218	-2.33774	-2.24600
H	6.50514	-3.03133	-1.44327
H	7.11745	-2.02421	-2.77794
H	5.57674	-2.90723	-2.95238
C	-5.06198	-0.32490	2.98949
H	-4.46237	-0.59352	3.87575
H	-6.10830	-0.18732	3.31598
H	-4.69857	0.65072	2.62341
C	4.28638	-3.27474	1.14899
H	4.64975	-2.23850	1.03436
C	-5.43330	-3.35096	2.40217
H	-5.62267	-4.10473	1.61891
H	-6.36622	-3.23236	2.98070
H	-4.66561	-3.76577	3.07420
C	3.02476	-2.32810	-4.72382
H	4.11169	-2.39597	-4.55438
H	2.84871	-1.56996	-5.50769
H	2.68689	-3.30245	-5.12090
C	-2.26345	1.95120	-3.42972
H	-2.64646	0.97863	-3.07358
C	5.06041	0.32718	2.98947
H	4.46073	0.59647	3.87547
H	6.10672	0.18989	3.31612
H	4.69705	-0.64875	2.62415
C	0.75916	-1.80239	-3.72664
H	0.31241	-2.74988	-4.07821
H	0.59606	-1.05033	-4.51802
H	0.20250	-1.47693	-2.83183
C	-2.31270	-2.73264	4.70668
H	-3.38837	-2.92180	4.56276
H	-2.20476	-1.97375	5.50200
H	-1.85422	-3.66827	5.07498
C	0.15182	1.92254	3.68585
H	-0.40942	2.81283	4.02212
H	0.07007	1.16482	4.48415
H	-0.35188	1.52534	2.78793
C	-0.15246	-1.91555	3.68404
H	0.41130	-2.80452	4.01954
H	-0.07094	-1.15737	4.48193
H	0.34872	-1.51749	2.78508
C	5.43219	3.35275	2.39938
H	5.62402	4.10496	1.61520
H	6.36392	3.23394	2.97979
H	4.66385	3.76975	3.06929
C	-0.75711	1.79469	-3.72579
H	-0.30873	2.74119	-4.07795
H	-0.59445	1.04160	-4.51627
H	-0.20160	1.46933	-2.83022
C	-6.20234	2.33626	-2.24863
H	-6.50487	3.03105	-1.44678
H	-7.11780	2.02261	-2.78018

```
H  -5.57654  2.90444 -2.95574
C  -3.35536  3.31635  2.37653
H  -2.49839  2.63034  2.25493
H  -3.89464  3.02270  3.29452
H  -2.95339  4.33418  2.53919
C  -4.59045 -4.76754 -1.39720
H  -4.18941 -5.78811 -1.53498
H  -5.19267 -4.52470 -2.29032
H  -5.26616 -4.78979 -0.52642
C   5.50650 -4.19470  1.36723
H   5.20528 -5.24557  1.52964
H   6.07463 -3.87472  2.25826
H   6.18913 -4.17432  0.50165
C  -2.55382 -3.69318 -2.46109
H  -1.76989 -2.92145 -2.36981
H  -3.14259 -3.46581 -3.36744
H  -2.05474 -4.66591 -2.62770
C   3.35301 -3.31383  2.37970
H   2.49579 -2.62835  2.25674
H   3.89121 -3.01903  3.29795
H   2.95140 -4.33171  2.54290
C   2.55236  3.69171 -2.46335
H   1.76819  2.92032 -2.37130
H   3.14068  3.46353 -3.36980
H   2.05354  4.66450 -2.63039
C   2.31530  2.73551  4.70457
H   3.39118  2.92212  4.55907
H   2.20669  1.97736  5.50051
H   1.85942  3.67241  5.07285
C  -3.02121  2.32272 -4.72523
H  -4.10816  2.39236 -4.55660
H  -2.84574  1.56356 -5.50824
H  -2.68164  3.29619 -5.12303
C  -5.50698  4.19812  1.36094
H  -5.20483  5.24885  1.52247
H  -6.07625  3.87971  2.25181
H  -6.18882  4.17747  0.49476
C   4.59005  4.76578 -1.40124
H   4.18956  5.78644 -1.53991
H   5.19184  4.52171 -2.29429
H   5.26603  4.78849 -0.53068
Mg  2.44366  0.09609  0.03875
C   0.64158 -1.33307  0.07185
O  -0.56708 -1.22331 -0.07413
C  -0.64499  1.33328  0.07735
O   0.56372  1.22643 -0.06945
```

TS(D-10)

SCF (BP86) Energy = -2810.34424135
Enthalpy 0K = -2808.901927
Enthalpy 298K = -2808.799923
Free Energy 298K = -2809.041488
Lowest Frequency = -309.1561 cm^{-1}
Second Frequency = 14.3893 cm^{-1}
SCF (BP86-D3BJ) Energy = -2810.86064730
SCF (Benzene) Energy = -2810.35815901
SCF (BS2) Energy = -4676.20864712

```
Si  5.39282  0.71536  1.46533
Si  4.91829 -1.77046 -1.70681
Si -4.91174  1.72116 -1.57084
Si -5.32835 -0.87250  1.49450
Mg -2.47310  0.03594 -0.10696
Na  0.38822  4.12281  0.03774
Na -0.53813 -3.69076  0.05698
N   3.76850  1.17248  0.89637
N   3.28816 -1.79167 -0.99396
N  -3.72272 -1.29332  0.84572
N  -3.24999  1.76621 -0.91303
C   3.33727  2.50058  1.10444
C  -2.59498  3.01197 -1.01710
C   1.68816 -3.21615 -2.26540
C   3.52452  3.51471  0.09115
C   2.53370 -2.97896 -1.11968
C   2.67715  2.90104  2.32554
C  -3.28742 -2.62128  1.05690
C  -1.84431  3.37881 -2.19839
C  -2.67743  3.99144  0.04429
C  -3.52397 -3.64471  0.06420
C   2.23472  4.22879  2.49248
H   1.75154  4.51215  3.43566
C   2.45610  1.90860  3.46756
H   2.81522  0.93233  3.09750
C  -6.50589 -0.12133  0.18235
H  -6.54115 -0.80839 -0.68529
H  -7.50824 -0.20619  0.65320
C   2.57334 -4.00433 -0.10355
C   3.06955  4.83402  0.30601
H   3.26254  5.59505 -0.46153
C   0.97408 -4.42728 -2.38441
H   0.36405 -4.59781 -3.28006
C  -6.28375  1.33394 -0.28576
H  -6.14191  2.00558  0.58303
H  -7.20571  1.70450 -0.78243
C   6.53082  0.01804  0.08604
H   6.53392  0.74114 -0.75283
H   7.54606  0.09644  0.53016
C  -1.69686  2.42142 -3.38392
H  -2.15115  1.46643 -3.06466
C  -2.60015 -3.01696  2.26510
C   6.31700 -1.42164 -0.43915
H   6.20115 -2.12961  0.40445
H   7.23544 -1.75666 -0.96637
C  -1.26861  4.66410 -2.30087
H  -0.73789  4.93380 -3.22231
C   2.42579  5.20825  1.50082
H   2.12448  6.24817  1.67417
C  -3.04963 -4.95769  0.26498
H  -3.24702 -5.71929 -0.49944
C  -3.42362  3.68241  1.34107
H  -3.82836  2.66283  1.21581
C   1.83750 -5.19774 -0.26366
H   1.91320 -5.97696  0.50528
C  -2.35770 -5.32247  1.43425
H  -2.02102 -6.35358  1.58699
C  -2.09506  5.26744 -0.11056
H  -2.22198  6.01057  0.68729
C   3.41812 -3.82646  1.15651
H   3.97009 -2.88050  1.01537
C   1.56302 -2.18049 -3.38277
H   2.18602 -1.32181 -3.07687
C  -4.27286 -3.31875 -1.22674
H  -4.70201 -2.31200 -1.07950
C  -2.14981 -4.34403  2.42260
H  -1.63597 -4.62378  3.35051
C  -1.39778  5.62500 -1.28136
```

H -0.99611 6.63679 -1.40955
C -5.22739 0.38237 2.93724
H -4.79689 -0.07720 3.84255
H -6.23326 0.75882 3.19665
H -4.60338 1.25416 2.67618
C   1.04697 -5.43143 -1.40388
H   0.51760 -6.38188 -1.53352
C   5.30368 -0.60186 2.85078
H   4.88420 -0.17793 3.77883
H   6.30866 -0.99716 3.08374
H   4.67074 -1.45749 2.55898
C   6.32917 2.23584 2.14862
H   6.65562 2.90388 1.33347
H   7.23320 1.90685 2.69026
H   5.71750 2.83659 2.84075
C -5.03723 0.40470 -2.95224
H -4.43569 0.68954 -3.83168
H -6.08322 0.27282 -3.28219
H -4.67573 -0.57867 -2.60402
C   4.24431 3.19529 -1.21806
H   4.56300 2.14126 -1.13459
C -5.43062 3.42219 -2.27694
H -5.68820 4.12224 -1.46365
H -6.33116 3.30008 -2.90428
H -4.65137 3.90410 -2.88688
C   3.28186 2.28202 4.72053
H   4.36012 2.34153 4.49997
H   3.13798 1.52920 5.51593
H   2.96945 3.26031 5.12882
C -2.35396 -2.02310 3.40078
H -2.73007 -1.04866 3.04349
C   5.04831 -0.44198 -3.07765
H   4.43414 -0.71314 -3.95304
H   6.09208 -0.32355 -3.41961
H   4.70314 0.54392 -2.72080
C   0.96546 1.76100 3.84275
H   0.55099 2.70518 4.24091
H   0.84165 0.99365 4.62699
H   0.35857 1.46531 2.97190
C -2.45362 2.92213 -4.63698
H -3.52980 3.05724 -4.44616
H -2.34421 2.19901 -5.46467
H -2.05084 3.88990 -4.98716
C   0.11047 -1.68593 -3.55057
H -0.56715 -2.50903 -3.84026
H   0.05117 -0.91612 -4.33914
H -0.27434 -1.23855 -2.61616
C -0.21985 2.15668 -3.74285
H   0.30064 3.08324 -4.04665
H -0.15023 1.45254 -4.59061
H   0.32074 1.71986 -2.88527
C   5.34814 -3.46843 -2.46965
H   5.47638 -4.23793 -1.68943
H   6.29612 -3.39999 -3.03121
H   4.56845 -3.82926 -3.15980
C -0.85479 -1.86122 3.73260
H -0.41271 -2.80668 4.09596
H -0.71545 -1.10745 4.52732
H -0.27977 -1.53280 2.85155
C -6.23372 -2.43219 2.12908
H -6.52297 -3.09154 1.29288
H -7.15751 -2.13972 2.65830
H -5.61981 -3.03062 2.82134
C -3.30411 -3.24197 -2.42776
H -2.50068 -2.50409 -2.25253
H -3.83367 -2.94863 -3.35163
H -2.82415 -4.22100 -2.61429
C -4.61080 4.64019 1.58319
H -4.27076 5.68149 1.73017
H -5.16750 4.34254 2.48910
H -5.31419 4.63590 0.73436
C   5.50429 4.06446 -1.42503
H   5.24744 5.13267 -1.54543
H   6.04352 3.74996 -2.33573
H   6.19774 3.98323 -0.57199
C -2.47744 3.67746 2.56259
H -1.65237 2.95606 2.43478
H -3.02561 3.40087 3.48060
H -2.03596 4.67728 2.73408
C   3.30160 3.31488 -2.43532
H   2.41726 2.66334 -2.32539
H   3.81948 3.02523 -3.36673
H   2.94628 4.35451 -2.56721
C   2.53157 -3.67829 2.41350
H   1.82281 -2.83846 2.30853
H   3.14595 -3.49025 3.31190
H   1.94504 -4.59713 2.60058
C   2.10241 -2.70817 -4.73203
H   3.15551 -3.02456 -4.65526
H   2.03850 -1.92376 -5.50695
H   1.51794 -3.57542 -5.08904
C -3.13770 -2.40811 4.67686
H -4.22032 -2.48665 4.48547
H -2.98495 -1.65233 5.46789
H -2.79809 -3.38080 5.07657
C -5.42736 -4.29873 -1.52277
H -5.05987 -5.31860 -1.73742
H -5.99671 -3.96412 -2.40756
H -6.12620 -4.36822 -0.67256
C   4.45068 -4.95774 1.34988
H   3.96195 -5.93397 1.52142
H   5.08957 -4.75044 2.22625
H   5.10466 -5.06274 0.46833
Mg 2.52795 -0.17820 -0.02436
C   0.78109 1.29227 -0.26730
O   0.07116 1.90901 0.47715
C -0.55267 -1.17790 0.03476
O   0.63939 -0.98856 0.35287

E

SCF (BP86) Energy = -2810.41400165
Enthalpy 0K = -2808.969349
Enthalpy 298K = -2808.867178
Free Energy 298K = -2809.112276
Lowest Frequency = 9.8972 cm^{-1}
Second Frequency = 14.1082 cm^{-1}
SCF (BP86-D3BJ) Energy = -2810.92469382
SCF (Benzene) Energy = -2810.42774781
SCF (BS2) Energy = -4676.27437903

Si 5.49186 -0.97495 -1.42871
Si 5.11515 1.74371 1.57035
Si -5.11482 -1.74332 1.57102
Si -5.49215 0.97575 -1.42773
Mg -2.65959 -0.08446 0.06336
Na 0.41681 -3.83173 -0.12640

Na	-0.41643	3.83116	-0.12664
N	3.85747	-1.32528	-0.81688
N	3.46631	1.76567	0.89710
N	-3.85734	1.32547	-0.81660
N	-3.46623	-1.76568	0.89723
C	3.35438	-2.63117	-0.98646
C	-2.74952	-2.97944	0.97908
C	1.96409	3.31598	2.14341
C	3.48960	-3.61830	0.06152
C	2.74929	2.97918	0.97970
C	2.68552	-3.04298	-2.20005
C	-3.35405	2.63127	-0.98639
C	-1.96424	-3.31704	2.14251
C	-2.77527	-3.93513	-0.10277
C	-3.48863	3.61840	0.06166
C	2.21238	-4.36498	-2.33709
H	1.73303	-4.66178	-3.27827
C	2.49394	-2.07306	-3.36590
H	2.89019	-1.10143	-3.02259
C	-6.65296	0.21639	-0.10320
H	-6.63800	0.87147	0.78946
H	-7.66451	0.35629	-0.54024
C	2.77467	3.93545	-0.10167
C	2.99530	-4.92749	-0.11985
H	3.13964	-5.66744	0.67720
C	1.28297	4.54940	2.20865
H	0.71871	4.79718	3.11649
C	-6.47672	-1.26528	0.30548
H	-6.35862	-1.90582	-0.58996
H	-7.41041	-1.62338	0.78894
C	6.65309	-0.21535	-0.10470
H	6.63885	-0.87048	0.78792
H	7.66443	-0.35493	-0.54234
C	-1.86209	-2.36185	3.33214
H	-2.42808	-1.45779	3.04632
C	-2.68566	3.04297	-2.20028
C	6.47666	1.26626	0.30415
H	6.35790	1.90679	-0.59122
H	7.41050	1.62460	0.78712
C	-1.28345	-4.55069	2.20704
H	-0.71919	-4.79911	3.11470
C	2.36480	-5.31986	-1.31555
H	2.02736	-6.35254	-1.45832
C	-2.99415	4.92749	-0.11990
H	-3.13800	5.66746	0.67722
C	-3.55726	-3.64547	-1.38240
H	-4.09069	-2.69625	-1.19792
C	2.07943	5.15763	0.00889
H	2.14784	5.88537	-0.80992
C	-2.36410	5.31977	-1.31587
H	-2.02655	6.35239	-1.45878
C	-2.08030	-5.15753	0.00704
H	-2.14899	-5.88480	-0.81215
C	3.55634	3.64646	-1.38165
H	4.09069	2.69772	-1.19738
C	1.86242	2.36015	3.33255
H	2.42899	1.45657	3.04635
C	-4.18439	3.26339	1.37471
H	-4.65189	2.27708	1.20601
C	-2.21231	4.36487	-2.33749
H	-1.73331	4.66159	-3.27888
C	-1.34045	-5.48654	1.15857
H	-0.84914	-6.46205	1.25000
C	-5.46506	-0.24475	-2.90243
H	-5.01891	0.21347	-3.80081
H	-6.48933	-0.56777	-3.16170
H	-4.88208	-1.15151	-2.66495
C	1.33961	5.48582	1.16068
H	0.84799	6.46112	1.25265
C	5.46358	0.24579	-2.90320
H	5.01767	-0.21268	-3.80158
H	6.48754	0.56970	-3.16260
H	4.87987	1.15202	-2.66551
C	6.35669	-2.58422	-1.99085
H	6.59030	-3.23109	-1.12769
H	7.30822	-2.34666	-2.49762
H	5.73925	-3.17542	-2.68663
C	-5.24350	-0.50219	3.02268
H	-4.65402	-0.83886	3.89202
H	-6.29157	-0.38058	3.35011
H	-4.87061	0.49583	2.73256
C	4.18589	-3.26319	1.37425
H	4.65276	-2.27660	1.20551
C	-5.61974	-3.47731	2.19618
H	-5.78041	-4.17063	1.35275
H	-6.56670	-3.41283	2.76005
H	-4.86154	-3.93259	2.85314
C	3.29453	-2.50747	-4.61503
H	4.37016	-2.61032	-4.39719
H	3.17991	-1.76571	-5.42541
H	2.93769	-3.47927	-5.00180
C	-2.49476	2.07302	-3.36621
H	-2.89113	1.10149	-3.02278
C	5.24493	0.50256	3.02189
H	4.65513	0.83860	3.89124
H	6.29313	0.38204	3.34931
H	4.87310	-0.49584	2.73174
C	1.00524	-1.88119	-3.73003
H	0.55200	-2.81985	-4.09776
H	0.89756	-1.12974	-4.53200
H	0.42073	-1.54044	-2.86013
C	-2.50232	-2.94851	4.61091
H	-3.56321	-3.20443	4.45775
H	-2.44421	-2.22181	5.44080
H	-1.98045	-3.86655	4.93673
C	0.40396	1.94215	3.61713
H	-0.21735	2.80719	3.91163
H	0.36392	1.21229	4.44461
H	-0.06081	1.47671	2.73182
C	-0.40340	-1.94490	3.61710
H	0.21731	-2.81046	3.91135
H	-0.36302	-1.21543	4.44490
H	0.06178	-1.47940	2.73205
C	5.61958	3.47776	2.19566
H	5.77649	4.17228	1.35253
H	6.56841	3.41399	2.75645
H	4.86283	3.93118	2.85559
C	-1.00623	1.88081	-3.73089
H	-0.55294	2.81937	-4.09882
H	-0.89902	1.12932	-4.53289
H	-0.42146	1.53999	-2.86119
C	-6.35670	2.58538	-1.98922
H	-6.58791	3.23311	-1.12606
H	-7.30950	2.34827	-2.49382
H	-5.74022	3.17553	-2.68676
C	-3.16364	3.10725	2.52368

```
H  -2.40123  2.34347  2.28585
H  -3.66107  2.80234  3.46146
H  -2.63600  4.05927  2.72100
C  -4.60840 -4.73002 -1.70172
H  -4.13810 -5.70601 -1.92026
H  -5.20013 -4.44373 -2.58890
H  -5.30532 -4.87705 -0.85985
C   5.29618 -4.26244  1.76138
H   4.89002 -5.26228  1.99965
H   5.83563 -3.90757  2.65684
H   6.02943 -4.38539  0.94696
C  -2.60986 -3.43259 -2.58451
H  -1.87822 -2.63092 -2.38235
H  -3.17630 -3.15515 -3.49121
H  -2.04568 -4.35532 -2.81707
C   3.16572 -3.10791  2.52387
H   2.40278 -2.34447  2.28670
H   3.66356 -2.80300  3.46143
H   2.63871 -4.06027  2.72125
C   2.60842  3.43254 -2.58317
H   1.87757  2.63030 -2.38037
H   3.17452  3.15541 -3.49017
H   2.04333  4.35477 -2.81556
C   2.50214  2.94657  4.61168
H   3.56287  3.20329  4.45874
H   2.44446  2.21939  5.44118
H   1.97962  3.86408  4.93797
C  -3.29566  2.50767 -4.61507
H  -4.37120  2.61078 -4.39687
H  -3.18150  1.76588 -5.42549
H  -2.93870  3.47938 -5.00195
C  -5.29389  4.26314  1.76277
H  -4.88703  5.26267  2.00118
H  -5.83306  3.90827  2.65838
H  -6.02751  4.38681  0.94878
C   4.60630  4.73192 -1.70171
H   4.13501  5.70744 -1.92021
H   5.19780  4.44600 -2.58916
H   5.30355  4.87974 -0.86026
Mg  2.65965  0.08446  0.06330
C   0.51104 -0.58297  0.02511
O  -0.05334 -1.68129 -0.23258
C  -0.51085  0.58281  0.02439
O   0.05365  1.68076 -0.23461
```

TS(E-10)

SCF (BP86) Energy = -2810.41042060
Enthalpy 0K = -2808.966672
Enthalpy 298K = -2808.865370
Free Energy 298K = -2809.104130
Lowest Frequency = -450.1413 cm^{-1}
Second Frequency = 16.6659 cm^{-1}
SCF (BP86-D3BJ) Energy = -2810.92678077
SCF (Benzene) Energy = -2810.42224762
SCF (BS2) Energy = -4676.26905002

```
Si -4.92619  1.59967 -1.61095
Si -5.20440 -0.85305  1.61740
Si  5.20433  0.85302  1.61738
Si  4.92615 -1.59974 -1.61090
Mg  2.41682 -0.08441 -0.02014
Na  0.35850  3.60321  0.02073
Na -0.35825 -3.60291  0.02082
N  -3.26133  1.68710 -0.97528
N  -3.59265 -1.25972  0.98571
N   3.26129 -1.68712 -0.97523
N   3.59258  1.25971  0.98567
C  -2.57346  2.91238 -1.11789
C   3.13180  2.58453  1.15258
C  -2.37435 -2.97481  2.31928
C  -2.63848  3.92927 -0.09248
C  -3.13177 -2.58450  1.15265
C  -1.77887  3.20473 -2.28964
C   2.57343 -2.91241 -1.11785
C   2.37446  2.97494  2.31921
C   3.37781  3.60066  0.15436
C   2.63846 -3.92932 -0.09246
C  -1.11775  4.44586 -2.40626
H  -0.54019  4.65546 -3.31448
C  -1.66162  2.20688 -3.44339
H  -2.12751  1.26973 -3.08952
C   6.26099 -1.22169 -0.28279
H   6.13668 -1.95154  0.54091
H   7.20458 -1.52412 -0.78552
C  -3.37771 -3.60068  0.15448
C  -1.95638  5.15467 -0.25505
H  -2.04612  5.92119  0.52482
C  -1.88205 -4.29167  2.44142
H  -1.32134 -4.56796  3.34283
C   6.42291  0.20890  0.28109
H   6.44901  0.94926 -0.54209
H   7.41599  0.29617  0.77106
C  -6.26102  1.22165 -0.28283
H  -6.13671  1.95150  0.54087
H  -7.20461  1.52407 -0.78556
C   2.10673  1.98754  3.45521
H   2.51514  1.01741  3.12117
C   1.77885 -3.20476 -2.28962
C  -6.42294 -0.20894  0.28106
H  -6.44899 -0.94931 -0.54213
H  -7.41604 -0.29623  0.77097
C   1.88229  4.29185  2.44133
H   1.32163  4.56822  3.34275
C  -1.19877  5.43042 -1.40679
H  -0.70310  6.39908 -1.53296
C   1.95638 -5.15473 -0.25505
H   2.04613 -5.92126  0.52482
C   4.18774  3.29261 -1.10406
H   4.53858  2.25195 -0.99011
C  -2.86736 -4.90493  0.32300
H  -3.08025 -5.66225 -0.44180
C   1.19877 -5.43047 -1.40679
H   0.70310 -6.39912 -1.53296
C   2.86761  4.90497  0.32288
H   3.08056  5.66225 -0.44195
C  -4.18772 -3.29276 -1.10392
H  -4.53865 -2.25212 -0.99001
C  -2.10670 -1.98735  3.45524
H  -2.51520 -1.01727  3.12118
C   3.46383 -3.71823  1.17568
H   3.96553 -2.74313  1.04932
C   1.11774 -4.44590 -2.40625
H   0.54017 -4.65549 -3.31447
C   2.11807  5.26668  1.45652
H   1.75252  6.29148  1.58330
C   5.06036 -0.25918 -2.96665
```

H   4.48516 -0.54207 -3.86429
H   6.11132 -0.10533 -3.27003
H   4.67169 0.71143 -2.61389
C -2.11775 -5.26654 1.45663
H -1.75208 -6.29129 1.58342
C -5.06033 0.25902 -2.96663
H -4.48546 0.54207 -3.86443
H -6.11132 0.10482 -3.26973
H -4.67124 -0.71144 -2.61395
C -5.47253 3.28006 -2.34452
H -5.72852 3.99473 -1.54377
H -6.37629 3.13974 -2.96311
H -4.70001 3.75295 -2.97083
C   5.09604 -0.47641 2.98893
H   4.61850 -0.06933 3.89644
H   6.10084 -0.83995 3.26937
H   4.50469 -1.34938 2.66463
C -3.46387 3.71819 1.17565
H -3.96541 2.74299 1.04938
C   6.05998 2.39349 2.35816
H   6.37721 3.09667 1.56964
H   6.96340 2.08756 2.91421
H   5.40690 2.94926 3.05017
C -2.43708 2.68518 -4.69395
H -3.50611 2.84381 -4.48206
H -2.35814 1.93937 -5.50489
H -2.02559 3.63753 -5.07478
C   1.66161 -2.20693 -3.44337
H   2.12745 -1.26975 -3.08949
C -5.09617 0.47638 2.98895
H -4.61861 0.06930 3.89647
H -6.10097 0.83989 3.26938
H -4.50484 1.34936 2.66466
C -0.19639 1.91045 -3.82837
H   0.31779 2.81213 -4.20714
H -0.15898 1.15585 -4.63277
H   0.37928 1.52687 -2.97082
C   2.83785 2.39778 4.75448
H   3.92573 2.48546 4.60119
H   2.66528 1.64937 5.54830
H   2.47345 3.37134 5.12948
C -0.60011 -1.80543 3.73224
H -0.12483 -2.75130 4.04869
H -0.44184 -1.07342 4.54295
H -0.06718 -1.43636 2.83784
C   0.60012 1.80576 3.73221
H   0.12493 2.75168 4.04865
H   0.44179 1.07377 4.54293
H   0.06716 1.43672 2.83782
C -6.06004 -2.39352 2.35817
H -6.37690 -3.09690 1.56968
H -6.96367 -2.08765 2.91390
H -5.40707 -2.94906 3.05049
C   0.19639 -1.91055 -3.82843
H -0.31772 -2.81223 -4.20726
H   0.15900 -1.15593 -4.63281
H -0.37935 -1.52701 -2.97091
C   5.47248 -3.28019 -2.34436
H   5.72827 -3.99487 -1.54354
H   6.37636 -3.13995 -2.96280
H   4.70003 -3.75303 -2.97079
C   2.56821 -3.63263 2.43063
H   1.82843 -2.81772 2.34269
H   3.17138 -3.43726 3.33494
H   2.01776 -4.57721 2.59800
C   5.42489 4.20578 -1.24936
H   5.13657 5.26191 -1.40031
H   6.02803 3.90059 -2.12242
H   6.06982 4.16170 -0.35636
C -4.55226 4.79782 1.36155
H -4.11190 5.79788 1.52646
H -5.17949 4.56432 2.23966
H -5.21047 4.86522 0.47979
C   3.31590 3.36416 -2.37798
H   2.45899 2.67172 -2.32041
H   3.90494 3.09792 -3.27354
H   2.92017 4.38485 -2.53525
C -2.56832 3.63290 2.43067
H -1.82839 2.81811 2.34287
H -3.17152 3.43752 3.33495
H -2.01805 4.57759 2.59798
C -3.31593 -3.36429 -2.37789
H -2.45908 -2.67176 -2.32039
H -3.90504 -3.09818 -3.27344
H -2.92011 -4.38495 -2.53511
C -2.83779 -2.39762 4.75453
H -3.92565 -2.48544 4.60123
H -2.66532 -1.64915 5.54831
H -2.47327 -3.37112 5.12958
C   2.43714 -2.68520 -4.69389
H   3.50617 -2.84382 -4.48195
H   2.35823 -1.93940 -5.50483
H   2.02568 -3.63756 -5.07474
C   4.55204 -4.79801 1.36179
H   4.11151 -5.79796 1.52691
H   5.17931 -4.56443 2.23985
H   5.21023 -4.86570 0.48004
C -5.42479 -4.20605 -1.24913
H -5.13639 -5.26216 -1.40006
H -6.02801 -3.90095 -2.12218
H -6.06968 -4.16200 -0.35611
Mg -2.41694 0.08435 -0.02018
C -0.10907 0.71476 -0.06806
O   0.88188 1.45241 -0.45313
C   0.10892 -0.71473 -0.06823
O -0.88207 -1.45228 -0.45341

A + CO

SCF (BP86) Energy = -2810.31405780
Enthalpy 0K = -2808.871251
Enthalpy 298K = -2808.767483
Free Energy 298K = -2809.015177
Lowest Frequency = 10.0589 cm^{-1}
Second Frequency = 12.6044 cm^{-1}
SCF (BP86-D3BJ) Energy = -2810.83383767
SCF (Benzene) Energy = -2810.32916518
SCF (BS2) Energy = -4676.18105810

Si 4.27795 -0.76242 -2.21927
Si 4.69900 0.97282 1.27141
Si -4.84335 -0.98448 1.38328
Si -4.56423 1.58437 -1.64704
Mg -1.93708 0.12417 -0.01350
Na -0.17760 -3.74573 -0.39769
Na 0.10610 3.56212 0.31434
N   2.78858 -1.32777 -1.41640

N	3.05819	1.47037	0.77726
N	-2.97829	1.75525	-0.84740
N	-3.20651	-1.38116	0.78154
C	2.25373	-2.57580	-1.77701
C	-2.77744	-2.71817	0.94065
C	1.76765	2.77046	2.46821
C	2.70305	-3.81960	-1.17400
C	2.58621	2.70229	1.27101
C	1.21326	-2.67677	-2.78352
C	-2.39949	3.03700	-0.92996
C	-2.13951	-3.18356	2.15857
C	-2.98688	-3.70422	-0.10507
C	-2.64462	4.04406	0.08413
C	0.66407	-3.93234	-3.12373
H	-0.09111	-3.98372	-3.91668
C	0.77738	-1.45076	-3.58527
H	1.17867	-0.57370	-3.04771
C	-5.98563	1.09902	-0.46161
H	-5.97464	1.80972	0.38740
H	-6.90775	1.34710	-1.02929
C	2.88164	3.96182	0.61364
C	2.11111	-5.04692	-1.54608
H	2.47904	-5.97190	-1.08688
C	1.24679	4.00638	2.91025
H	0.64082	4.03027	3.82327
C	-6.07199	-0.35185	0.05010
H	-6.04646	-1.06323	-0.79798
H	-7.06222	-0.51383	0.52688
C	5.79635	-0.58485	-1.05476
H	5.85198	-1.48872	-0.41911
H	6.67688	-0.65247	-1.72786
C	-2.05262	-2.30301	3.41092
H	-2.30952	-1.27722	3.09294
C	-1.53999	3.41723	-2.03533
C	5.91650	0.68688	-0.18533
H	5.89347	1.59001	-0.82544
H	6.91697	0.70739	0.29742
C	-1.67796	-4.51243	2.25312
H	-1.19393	-4.83915	3.18075
C	1.09051	-5.12175	-2.50845
H	0.66336	-6.08751	-2.79883
C	-2.05336	5.32254	-0.01631
H	-2.26958	6.06885	0.75776
C	-3.81165	-3.37397	-1.34973
H	-3.87210	-2.27189	-1.39188
C	2.32926	5.16869	1.09622
H	2.56647	6.10398	0.57512
C	-1.22009	5.67089	-1.09163
H	-0.78282	6.67220	-1.16015
C	-2.51994	-5.02967	0.05242
H	-2.71761	-5.76111	-0.74067
C	3.80503	4.03742	-0.60228
H	4.19069	3.01654	-0.76110
C	1.53006	1.53496	3.33678
H	1.86014	0.66378	2.74252
C	-3.57733	3.76836	1.26229
H	-3.95019	2.74079	1.11337
C	-0.97718	4.71148	-2.08797
H	-0.34621	4.98027	-2.94333
C	-1.84307	-5.44067	1.21317
H	-1.49435	-6.47325	1.32294
C	-4.49652	0.28204	-3.04667
H	-3.88442	0.63824	-3.89234
H	-5.51010	0.06555	-3.42948
H	-4.06221	-0.67059	-2.69914
C	1.50558	5.20819	2.23193
H	1.09502	6.15615	2.59447
C	3.94659	0.94778	-3.00007
H	3.22977	0.86505	-3.83453
H	4.87842	1.39335	-3.39322
H	3.53030	1.64745	-2.25678
C	4.82179	-1.93412	-3.64791
H	5.86546	-2.26719	-3.51457
H	4.77649	-1.39890	-4.61244
H	4.19117	-2.83376	-3.73360
C	-4.75516	0.33363	2.76674
H	-4.33580	-0.09719	3.69178
H	-5.76049	0.72703	3.00245
H	-4.12072	1.18722	2.47736
C	3.87996	-3.85382	-0.19658
H	4.02459	-2.81423	0.14795
C	-5.75237	-2.51947	2.11425
H	-6.64761	-2.75948	1.51503
H	-6.10464	-2.30142	3.13764
H	-5.12795	-3.42594	2.15773
C	1.40001	-1.47558	-5.00201
H	2.49758	-1.54576	-4.96479
H	1.13221	-0.56048	-5.56044
H	1.02748	-2.34070	-5.58020
C	-1.27104	2.46203	-3.19850
H	-1.69492	1.48811	-2.89680
C	4.66131	-0.63339	2.30895
H	4.18668	-0.45807	3.28948
H	5.68753	-1.00283	2.48681
H	4.09664	-1.43308	1.80123
C	-0.74937	-1.29571	-3.69715
H	-1.21606	-2.12745	-4.25335
H	-1.00481	-0.36737	-4.23509
H	-1.22199	-1.23925	-2.69866
C	-3.08969	-2.76433	4.46626
H	-4.11249	-2.78937	4.06531
H	-3.07788	-2.08530	5.33757
H	-2.84830	-3.77951	4.83063
C	0.05243	1.33186	3.72275
H	-0.34820	2.18245	4.30274
H	-0.05940	0.43265	4.34947
H	-0.58041	1.19378	2.82645
C	-0.66414	-2.27225	4.08345
H	-0.31157	-3.28404	4.35220
H	-0.71984	-1.69337	5.02195
H	0.09956	-1.80695	3.44606
C	5.54802	2.31118	2.34817
H	6.03897	3.07694	1.72499
H	6.33243	1.83928	2.96563
H	4.85025	2.82981	3.02501
C	0.23242	2.27233	-3.47619
H	0.71976	3.21481	-3.78289
H	0.38921	1.54765	-4.29377
H	0.75853	1.88398	-2.58479
C	-5.15618	3.23842	-2.41368
H	-5.61535	3.88663	-1.64804
H	-5.93017	3.03191	-3.17411
H	-4.35343	3.81879	-2.89443
C	-2.83795	3.82365	2.61663
H	-2.00392	3.10154	2.65817
H	-3.52437	3.58130	3.44697

```
H  -2.42394  4.83020  2.81062
C  -5.25107 -3.92661 -1.21900
H  -5.23900 -5.03056 -1.16594
H  -5.85804 -3.63674 -2.09510
H  -5.75332 -3.55444 -0.31452
C   5.17408 -4.30302 -0.91913
H   5.07268 -5.34132 -1.28406
H   6.03412 -4.27142 -0.22682
H   5.40805 -3.66676 -1.78497
C  -3.19557 -3.89387 -2.66398
H  -2.14890 -3.57356 -2.78952
H  -3.76602 -3.51365 -3.52927
H  -3.22027 -4.99737 -2.72124
C   3.66343 -4.76058  1.03512
H   2.74686 -4.50601  1.58864
H   4.51654 -4.65747  1.72789
H   3.60884 -5.82806  0.75411
C   3.04690  4.46446 -1.87801
H   2.21607  3.77711 -2.10885
H   3.72171  4.47050 -2.75196
H   2.62783  5.48200 -1.77158
C   2.40074  1.59432  4.61562
H   3.46795  1.72620  4.37593
H   2.29535  0.66292  5.19860
H   2.09608  2.43895  5.26018
C  -1.97751  2.92293 -4.49577
H  -3.06731  3.00841 -4.36567
H  -1.78842  2.20391 -5.31297
H  -1.59979  3.90772 -4.82566
C  -4.79029  4.72506  1.28342
H  -4.47906  5.77128  1.45635
H  -5.48437  4.44712  2.09588
H  -5.34760  4.69617  0.33332
C   5.00454  4.98432 -0.37075
H   4.67967  6.03722 -0.28643
H   5.70850  4.92499 -1.21927
H   5.55420  4.73237  0.54994
Mg  1.78975  0.06501 -0.13238
C   1.47311 -1.70357  1.22381
O   1.10306 -2.80946  1.38247
C   2.87032 -2.27488  5.61961
O   3.11080 -3.39755  5.56550
```

TS(A-10)

SCF (BP86) Energy = -2810.30220475
Enthalpy 0K = -2808.858283
Enthalpy 298K = -2808.756527
Free Energy 298K = -2808.995558
Lowest Frequency = -196.0547 cm^{-1}
Second Frequency = 18.5310 cm^{-1}
SCF (BP86-D3BJ) Energy = -2810.81867940
SCF (Benzene) Energy = -2810.32021410
SCF (BS2) Energy = -4676.16708095

```
Si  4.43093 -1.37789 -2.03596
Si  5.16834  0.74282  1.24805
Si -5.28921 -0.57539  1.25618
Si -4.61465  2.00715 -1.73100
Mg -2.34770  0.29313  0.05999
Na -0.49759 -3.79855 -0.03363
Na  0.45614  3.40773  0.23796
N   2.98495 -1.74942 -1.06400
N   3.48532  1.21027  0.86949
N  -3.03538  1.98256 -0.89149
N  -3.69099 -1.14570  0.69231
C   2.28887 -2.94786 -1.31524
C  -3.37027 -2.51594  0.85789
C   2.32612  2.88295  2.30449
C   2.54726 -4.15217 -0.55116
C   3.07314  2.53536  1.10925
C   1.27574 -3.02901 -2.34613
C  -2.26244  3.16133 -0.97110
C  -2.95990 -3.05089  2.13741
C  -3.45737 -3.44577 -0.24933
C  -2.36122  4.18829  0.04408
C   0.60866 -4.24849 -2.60059
H  -0.11960 -4.29540 -3.41954
C   0.93970 -1.82318 -3.22284
H   1.48455 -0.96545 -2.79029
C  -6.10913  1.61420 -0.59724
H  -6.06110  2.28881  0.27936
H  -6.97687  1.97846 -1.18753
C   3.37243  3.61989  0.19345
C   1.84547 -5.34155 -0.83829
H   2.07812 -6.24516 -0.26274
C   1.90170  4.21196  2.52511
H   1.35949  4.44636  3.44846
C  -6.37265  0.15752 -0.15118
H  -6.33557 -0.52152 -1.02266
H  -7.41017  0.07600  0.23564
C   6.00418 -1.07576 -0.97483
H   6.06079 -1.89737 -0.23494
H   6.83825 -1.27096 -1.68204
C  -2.99610 -2.18874  3.39941
H  -3.70044 -1.36788  3.19123
C  -1.34794  3.40282 -2.06718
C   6.24162  0.28690 -0.28046
H   6.18820  1.10869 -1.01923
H   7.28299  0.31909  0.10524
C  -2.55109 -4.39495  2.24596
H  -2.22249 -4.77274  3.22005
C   0.88200 -5.41087 -1.85990
H   0.37618 -6.35471 -2.09070
C  -1.58660  5.36436 -0.05028
H  -1.69172  6.13306  0.72452
C  -4.09226 -3.03396 -1.57800
H  -3.93653 -1.94407 -1.67791
C   2.92490  4.93156  0.46482
H   3.17847  5.73140 -0.24102
C  -0.71253  5.58966 -1.12630
H  -0.13610  6.51768 -1.19467
C  -3.05713 -4.79153 -0.08132
H  -3.15230 -5.48513 -0.92460
C   4.22179  3.40032 -1.05715
H   4.37528  2.31150 -1.13689
C   2.06942  1.85480  3.40105
H   2.28767  0.86690  2.95719
C  -3.32987  4.04500  1.21654
H  -3.92532  3.14088  1.00317
C  -0.60511  4.60365 -2.12022
H   0.06167  4.77771 -2.97277
C  -2.57837 -5.27200  1.14998
H  -2.28161 -6.32051  1.26418
C  -4.66348  0.76280 -3.18235
H  -3.98139  1.05968 -3.99603
H  -5.68325  0.69647 -3.60221
```

H	-4.37410	-0.25082	-2.85587
C	2.18749	5.24420	1.61688
H	1.86189	6.27079	1.81381
C	4.14570	0.16468	-3.13267
H	3.51574	-0.08127	-4.00405
H	5.10375	0.56397	-3.51212
H	3.64338	0.97121	-2.57411
C	4.88903	-2.81696	-3.22021
H	5.61920	-3.50931	-2.76898
H	5.35317	-2.40458	-4.13360
H	4.01344	-3.41303	-3.52467
C	-5.14430	0.80269	2.58160
H	-4.86838	0.39679	3.56921
H	-6.11088	1.32644	2.69345
H	-4.39012	1.56011	2.31079
C	3.61943	-4.18287	0.53727
H	3.84954	-3.12659	0.76430
C	-6.31266	-1.98758	2.04320
H	-7.32206	-1.60681	2.27928
H	-5.86558	-2.34573	2.98427
H	-6.43048	-2.85982	1.38040
C	1.42222	-2.02117	-4.67900
H	2.50499	-2.21644	-4.72792
H	1.20895	-1.12251	-5.28531
H	0.90632	-2.87433	-5.15590
C	-1.20364	2.41174	-3.22292
H	-1.76618	1.50835	-2.92707
C	5.17200	-0.79376	2.37886
H	4.76553	-0.55632	3.37513
H	6.20483	-1.16433	2.51131
H	4.57890	-1.62766	1.96646
C	-0.56561	-1.49497	-3.21719
H	-1.16428	-2.29979	-3.67526
H	-0.76969	-0.57493	-3.78894
H	-0.93605	-1.33087	-2.18519
C	-3.51246	-2.94824	4.64055
H	-4.46036	-3.47595	4.43919
H	-3.68337	-2.23991	5.46976
H	-2.78336	-3.69454	5.00360
C	0.61504	1.83642	3.90570
H	0.33400	2.77891	4.40977
H	0.47573	1.01401	4.62779
H	-0.09231	1.65614	3.07622
C	-1.63821	-1.52887	3.70419
H	-0.85983	-2.28697	3.90067
H	-1.71210	-0.87703	4.59282
H	-1.28859	-0.91346	2.85931
C	6.08958	2.14119	2.19069
H	7.07425	2.34650	1.73650
H	6.27053	1.83959	3.23678
H	5.52234	3.08588	2.20966
C	0.26303	2.01112	-3.47989
H	0.87774	2.87853	-3.77738
H	0.32944	1.27870	-4.30287
H	0.72339	1.54911	-2.58694
C	-4.99103	3.75458	-2.41231
H	-5.28953	4.43691	-1.59817
H	-5.83306	3.70113	-3.12462
H	-4.13535	4.21553	-2.93019
C	-2.58371	3.82153	2.54990
H	-1.94575	2.91988	2.51284
H	-3.29483	3.68927	3.38423
H	-1.93591	4.68300	2.79626
C	-5.61917	-3.28869	-1.55042
H	-5.82450	-4.36785	-1.43133
H	-6.08602	-2.95559	-2.49452
H	-6.11151	-2.75918	-0.72223
C	4.91815	-4.84442	0.01840
H	4.73922	-5.90258	-0.24573
H	5.70258	-4.81810	0.79534
H	5.30546	-4.33674	-0.87775
C	-3.50323	-3.74598	-2.81267
H	-2.40164	-3.72503	-2.82412
H	-3.86221	-3.26260	-3.73750
H	-3.81768	-4.80415	-2.86274
C	3.17206	-4.88628	1.83719
H	2.23688	-4.46299	2.23503
H	3.95102	-4.77580	2.61111
H	3.02233	-5.97092	1.68500
C	3.52617	3.88334	-2.34701
H	2.54162	3.40579	-2.48149
H	4.13936	3.63582	-3.23084
H	3.37474	4.97813	-2.34639
C	3.04780	2.05826	4.58146
H	4.09649	2.04336	4.24412
H	2.91483	1.26097	5.33339
H	2.86888	3.02882	5.07895
C	-1.82145	2.96761	-4.52827
H	-2.88428	3.22794	-4.40412
H	-1.74566	2.22185	-5.33953
H	-1.29043	3.87760	-4.86146
C	-4.29896	5.24140	1.33746
H	-3.77054	6.17369	1.60698
H	-5.04655	5.04838	2.12638
H	-4.83759	5.42464	0.39311
C	5.60746	4.07542	-0.92915
H	5.50744	5.17328	-0.85027
H	6.22545	3.85908	-1.81859
H	6.15355	3.72488	-0.03991
Mg	2.24739	-0.33708	0.26827
C	1.26205	-1.75595	1.70167
O	0.57235	-2.75104	1.66397
C	1.63785	-1.35860	3.51573
O	2.46419	-1.88482	4.16654
